# Complement 5a receptor 2 attenuates diabetic kidney disease by promoting mitochondria-associated endoplasmic reticulum membrane formation mediated by PSS-MFN2 interaction

**DOI:** 10.1038/s41421-026-00873-w

**Published:** 2026-03-31

**Authors:** Yi-yang Zhao, Yi-hui Wang, Zi-han Li, Dong-yuan Chang, Lin Nie, Ming-hui Zhao, Sydney Chi Wai Tang, Min Chen

**Affiliations:** 1 Renal Division, Department of Medicine, Peking University First Hospital, Peking University Institute of Nephrology, Key Laboratory of Renal Disease, Ministry of Health of China, Key Laboratory of Chronic Kidney Disease Prevention and Treatment (Peking University), Ministry of Education, Beijing, China; 2 Research Units of Diagnosis and Treatment of Immune-mediated Kidney Diseases, Chinese Academy of Medical Sciences, Beijing, China; 3 Peking-Tsinghua Center for Life Sciences, Beijing, China; 4 Laboratory of Electron Microscopy, Pathological Center, Peking University First Hospital, Beijing, China; 5 Division of Nephrology, Department of Medicine, School of Clinical Medicine, The University of Hong Kong, Queen Mary Hospital, Hong Kong, China; 6 State Key Laboratory of Vascular Homeostasis and Remodelling, Peking University, Beijing, China

**Keywords:** Energy metabolism, Membrane fusion, Pattern recognition receptors, Mechanisms of disease

## Abstract

Complement-mediated metabolic disorders are considered important contributors to the pathogenesis of diabetic kidney disease (DKD). This study investigated the non-canonical roles of complement 5a receptor 2 (C5aR2) in metabolism and its underlying molecular mechanisms in DKD. In patients with DKD, we found that C5aR2 expression was upregulated in the tubulointerstitium and correlated with both disease severity and adverse renal outcomes. C5aR2 deficiency in diabetic mice exacerbated the DKD phenotype, including pronounced lipid accumulation, mitochondrial and endoplasmic reticulum (ER) dysfunction, and reduced phosphatidylserine (PS) levels in the kidney. Mechanistically, C5aR2 activated cellular Fos proto-oncogene (c-FOS) nuclear translocation, upregulated phosphatidylserine synthase (PSS) expression, and promoted the interaction between PSS and mitochondrial fusion protein 2 (MFN2), which facilitated mitochondria-associated ER membrane (MAM) formation and PS biosynthesis, improving mitochondrial and ER function. Treatment with the C5aR2-specific agonist P59 ameliorated the DKD phenotype, improved PS homeostasis and MAM formation, and thereby reversed lipid accumulation, ER stress, and mitochondrial dysfunction in *db/db* mice. Single-cell RNA sequencing (scRNA-seq) analysis revealed that P59 restored *Pss* expression in injured proximal tubular cells. These results highlight C5aR2 activation as a promising strategy for DKD treatment.

## Introduction

Diabetic kidney disease (DKD) represents a major microvascular complication of diabetes, with approximately 30%–50% of DKD patients progressing to end-stage kidney disease (ESKD)^1^. The pathogenesis of DKD is complex and not fully clear. Current therapeutic strategies are limited to supportive therapies, including blood pressure control, blood glucose control, and hemodynamic improvement, but these measures only partially delay DKD progression. Therefore, there is an urgent need to identify the molecular mechanisms underlying DKD and novel therapeutic targets.

The complement system, a highly conserved element of innate immunity, is closely associated with the development and progression of DKD^2,3^. Increasing preclinical evidence suggests that inhibiting complement overactivation protects against DKD by reducing inflammation and fibrosis^3–6^. It is particularly attractive to target complement 5a (C5a), as blocking it can reduce inflammation while maintaining the essential immune defense capabilities of the complement system^7^. As a terminal effector of complement activation, C5a exerts its biological effects by binding to two distinct receptors: C5aR1 (CD88) and C5aR2 (GPR77, also known as C5L2)^8,9^. Both C5aR1 and C5aR2 are seven-transmembrane domain receptors predominantly expressed on the cell surface^10,11^. Unlike the canonical G protein-coupled receptor (GPCR) C5aR1, C5aR2 does not engage G proteins; instead, it preferentially recruits the scaffold protein β-arrestin to mediate downstream signaling^12^. Owing to early conflicting findings on C5aR2 and limited knowledge of its functions, C5aR2 has been described as an “enigmatic receptor”^13–15^. Several studies have demonstrated that inhibiting the C5a-C5aR1 axis in DKD markedly alleviates proteinuria, renal inflammation, mitochondrial dysfunction, and oxidative stress^3,16^. However, simultaneous blockade of both C5aR1 and C5aR2 using the C5a inhibitor NOX-D21 in diabetic mice did not obviously improve the renal phenotype, especially proteinuria^17^, suggesting a potential renoprotective role of C5aR2 in DKD. In addition, recent studies have suggested that C5aR2 plays a role in regulating lipid metabolism. The single-nucleotide polymorphism (SNP) S323I in C5aR2 was identified as a potential molecular basis for familial mixed hyperlipidemia^18^. Furthermore, C5aR2 deficiency led to ectopic lipid deposition and inflammatory infiltration in obese mice^19^. Therefore, it is reasonable to speculate that C5aR2 can ameliorate DKD by modulating metabolic inflammation.

The progression of metabolic inflammation in DKD is driven by an interconnected network of factors, including lipotoxicity, oxidative stress, endoplasmic reticulum (ER) stress, and mitochondrial dysfunction^20,21^. Notably, mitochondrial and ER functions depend extensively on phospholipid synthesis and interorganellar phospholipid transport^22^. Previous studies have reported that one of the most important characteristics of lipid remodeling in the renal cortex in DKD patients is changes in phospholipid profiles, particularly decreases in the phosphatidylserine (PS) content^23^. In mammalian cells, the biosynthesis of phosphatidylserine in the ER is catalyzed by two phosphatidylserine synthases (PSSs), i.e., PSS1 and PSS2, which are localized to mitochondria-associated ER membranes (MAMs) and utilize phosphatidylcholine (PC) and phosphatidylethanolamine (PE) as substrates, respectively^24^. In Drosophila, *Pss* knockdown in salivary glands significantly reduces the PS content, leading to lipid accumulation and mitochondrial dyshomeostasis^25^. In DKD patients, reduced MAM formation in proximal tubular epithelial cells (PTECs) is closely associated with ectopic renal lipid deposition and renal injury^26^. However, the upstream factors that disrupt MAM formation and their relationship with PS in DKD remain unclear.

In the present study, C5aR2 expression was significantly upregulated in the tubulointerstitium of DKD patients and correlated with both disease severity and adverse renal outcomes. In animal studies, *C5ar2* knockout (*C5ar2*^−/−^) mice developed a more severe DKD phenotype in a streptozotocin (STZ)/high-fat diet (HFD)-induced diabetic model, with pronounced lipid accumulation and mitochondrial and ER dysfunction. Mechanistically, C5aR2 mediated the interaction between PSS and mitochondrial fusion protein 2 (MFN2) to facilitate MAM formation and PS biosynthesis. Furthermore, treatment with the C5aR2-specific agonist P59 markedly restored PS metabolic homeostasis and MAM formation, providing protection against kidney injury in diabetes. Overall, our findings suggest that C5aR2 represents a promising therapeutic target for DKD.

## Results

### C5aR2 expression is upregulated in the renal tubulointerstitium of patients with DKD

**Fig. 1:**
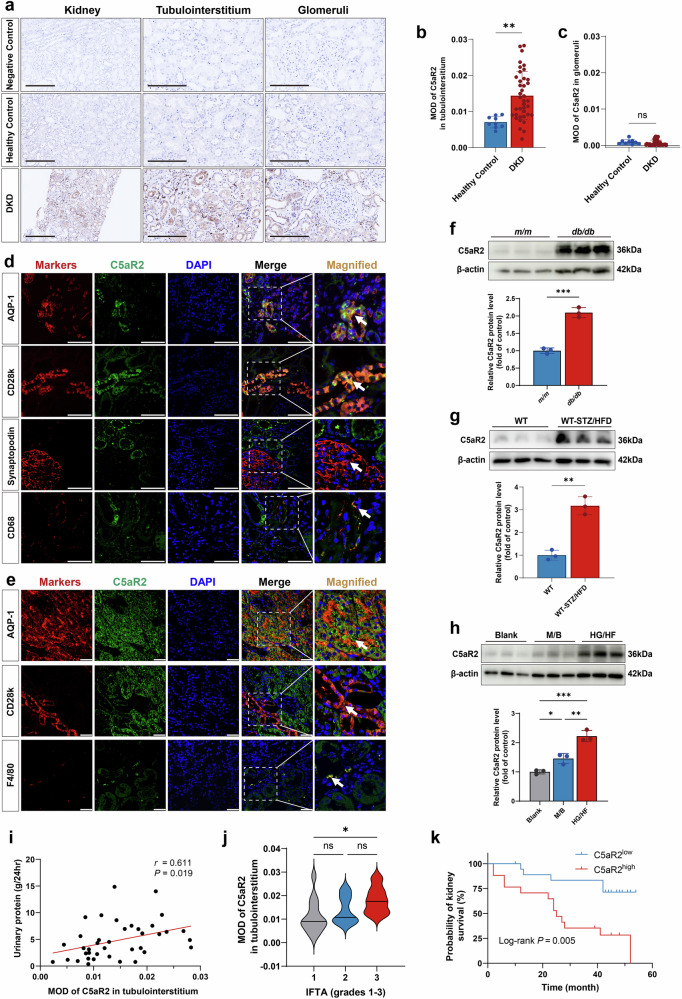
C5aR2 expression was upregulated in the renal tubulointerstitium of patients with DKD, consistent with the in vivo and in vitro DKD models. **a** Representative immunohistochemistry (IHC) images of C5aR2 staining showing glomerular and tubulointerstitial expression in kidney tissues from DKD patients and healthy control kidney tissues (scale bars = 200 μm). **b** Tubulointerstitial C5aR2 staining in kidney tissues from DKD patients (*n* = 39 each) was significantly stronger than that in kidney tissues from healthy controls (*n* = 9 each). **c** In glomeruli, C5aR2 expression was not detected in kidney tissues from DKD patients (*n* = 39 each) or healthy control kidney tissues (*n* = 9 each). **d** Representative immunofluorescence (IF) images showing the expression of C5aR2 in proximal tubular epithelial cells (PTECs) (aquaporin 1 (AQP-1)), distal tubular epithelial cells (calbindin-D-28K (CD28k)), podocytes (synaptopodin), and macrophages (CD68) in kidney tissues from DKD patients (scale bars = 100 μm). **e** Representative IF images showing the expression of C5aR2 in PTECs (AQP-1), distal tubular epithelial cells (CD28k), and macrophages (F4/80) in kidney tissues from *db/db* mice (scale bars = 100 μm). **f**, **g** Representative western blotting images and quantitative analysis of C5aR2 expression in renal cortex tissues from *db/db* mice and streptozotocin (STZ)/ high-fat diet (HFD)-induced diabetic mice compared with the respective controls (*n* = 3 per group). **h** Representative western blotting images and quantitative analysis of C5aR2 expression in TCMK-1 cells treated with mannitol and endotoxin-free BSA (M/B) or a high-glucose and high-fat (HG/HF) diet (*n* = 3 independent replicates). **i** Tubulointerstitial C5aR2 expression was positively correlated with 24-h urinary protein levels in DKD patients. **j** Violin plot showing the associations between interstitial fibrosis and tubular atrophy (IFTA) grades and tubulointerstitial C5aR2 expression levels. **k** Kaplan‒Meier survival curves for kidney survival in DKD patients (*n* = 39 each) classified by the expression levels of C5aR2 in the renal tubulointerstitium (*n* = 19, C5aR2^low^; *n* = 20, C5aR2^high^). The data were analyzed via unpaired two-tailed Student’s *t*-test (**b**, **c**, **f**, **g**), Spearman analysis (**i**), Kruskal‒Wallis analysis (**k**), log-rank test (**k**), and two-sided one-way ANOVA with Tukey’s test (**h**, **j**). The data are expressed as the means ± SD. ns not significant; *P < 0.05, **P < 0.01, and ***P < 0.001. DAPI 4’,6-diamidino-2-phenylindole, MOD mean optical density.

To investigate whether C5aR2 is dysregulated in the kidney tissues of DKD patients, we conducted immunohistochemical staining for C5aR2 in kidney biopsies from 39 DKD patients (Fig. 1a; cohort details in Supplementary Table S1). C5aR2 immunostaining in the renal tubulointerstitium of patients with DKD was significantly stronger than that in healthy controls, whereas no detectable staining was observed in the glomeruli (Fig. 1b, c). Immunofluorescence (IF) colocalization analysis revealed that C5aR2 predominantly colocalized with PTECs among resident renal cells and was also observed on cluster of differentiation 68 (CD68)-positive macrophages (Fig. 1d). Notably, this expression pattern was consistent with findings observed in *db/db* mice. IF analysis of *db/db* mouse kidneys similarly revealed predominant C5aR2 localization in PTECs, with expression also detected on EGF-like module-containing mucin-like hormone receptor-like 1 (F4/80)-positive macrophages (Fig. 1e). Western blotting analysis demonstrated that C5aR2 protein levels in the renal cortex were significantly greater in both *db/db* mice and STZ/HFD-induced diabetic mice than in their respective controls (Fig. 1f, g). In vitro, C5aR2 protein levels significantly increased in mouse renal tubular epithelial cells (TCMK-1 cells, TECs) under high-glucose and high-fat (HG/HF) conditions (Fig. 1h).

Correlation analysis between C5aR2 expression in the tubulointerstitium and clinicopathological parameters was subsequently performed in DKD patients. The expression of C5aR2 in the renal tubulointerstitium of DKD patients was positively correlated with the 24-h urinary protein level (*r* = 0.611, *P* = 0.019; Fig. 1i). Moreover, patients with high renal tubulointerstitial C5aR2 expression exhibited more severe interstitial fibrosis and tubular atrophy (IFTA) (Fig. 1j) and had a significantly greater risk of progression to ESKD (*P* = 0.005; Fig. 1k).

Collectively, these results indicate that C5aR2 expression is upregulated in the diabetic kidney and is associated with both disease severity and adverse renal outcomes in DKD patients, suggesting a potential role for C5aR2 in pathogenesis of the disease.

### C5aR2 deficiency exacerbates tubulointerstitial injury in a mouse model of diabetes induced by STZ/HFD

**Fig. 2:**
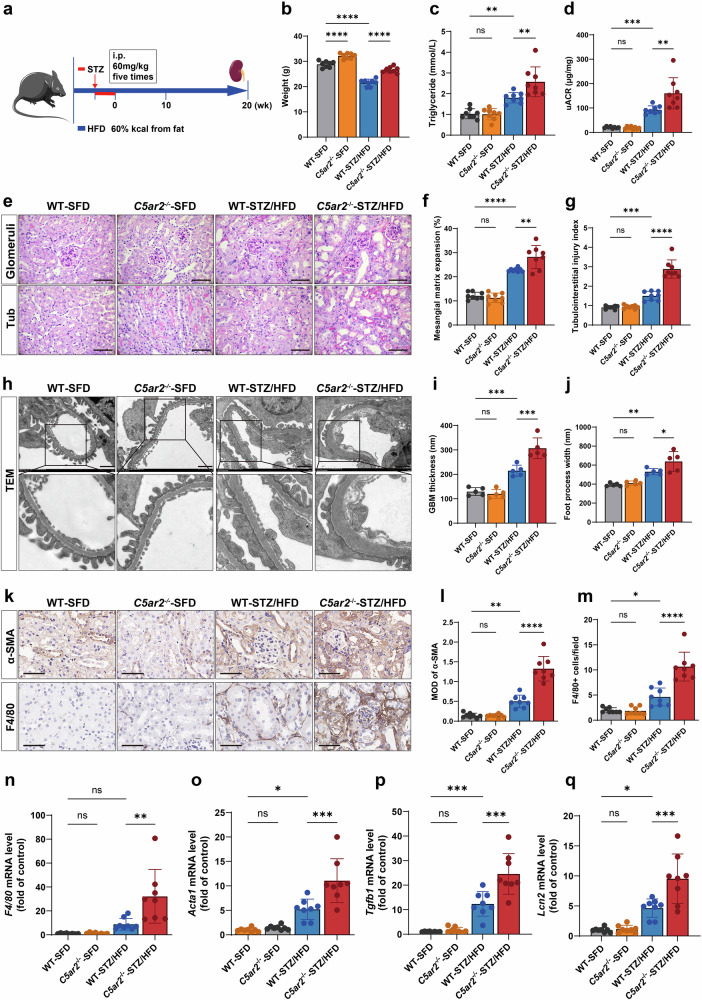
C5aR2 deficiency aggravated tubulointerstitial injury in STZ/HFD-treated mice. **a** Schematic overview of the experimental strategy for generating the STZ/HFD-induced *C5ar2*^*−/−*^ mouse model of DKD. **b** Comparison of weights in different groups of mice (*n* = 8 per group). **c** Comparison of blood triglyceride levels in different groups of mice (*n* = 8 per group). **d** Comparison of the urine albumin-to-creatinine ratio (uACR) in different groups of mice (*n* = 8 per group). **e** Representative images of periodic acid–Schiff (PAS) staining of the glomeruli and tubulointerstitium (scale bars = 50 μm). Tub, tubulointerstitium. **f**, **g** Quantitative analysis of mesangial matrix expansion (**f**) and the tubulointerstitial injury index (**g**) in different groups of mice (*n* = 8 per group). **h** Representative transmission electron microscopy (TEM) micrographs (scale bars = 1 µm). **i**, **j** Quantification of glomerular basement membrane (GBM) thickness (**i**) and foot process width (**j**) in different groups of mice (*n* = 5 per group). **k** Representative IHC images of α-SMA and F4/80 staining (scale bars = 60 µm). **l**, **m** Quantitative analysis of α-SMA (**l**) and F4/80 (**m**) in different groups of mice (*n* = 8 per group). **n**–**q** Comparison of inflammatory and fibrotic gene expression by qRT-PCR in different groups of mice (*n* = 8 per group). The data in the graphs are presented as the means ± SD. The data were analyzed by two-sided one-way ANOVA with Tukey’s test. ns not significant; *P < 0.05; **P < 0.01; ***P < 0.001; ****P < 0.0001.

We generated a *C5ar2*^−/−^ mouse model (Supplementary Fig. S1a) and verified the absence of *C5ar2* expression in the renal cortex by polymerase chain reaction (PCR) and quantitative real-time PCR (qRT-PCR) (Supplementary Fig. S1b and c). To investigate the role of C5aR2 in the development of DKD, wild-type (WT) and *C5ar2*^−/−^ mice were subjected to STZ/HFD treatment to induce DKD (Fig. 2a). As shown in Supplementary Table S2, there was no significant difference in fasting blood glucose or total blood cholesterol between diabetic WT and diabetic *C5ar2*^−/−^ mice, whereas body weight and blood triglyceride levels were significantly greater in diabetic *C5ar2*^−/−^ mice (Fig. 2b, c). Moreover, the urine albumin-to-creatinine ratio (uACR) was significantly greater in diabetic *C5ar2*^−/−^ mice than in their diabetic WT counterparts (Fig. 2d). Consistent with impaired renal function, periodic acid–Schiff (PAS) staining revealed more severe tubulointerstitial damage and mesangial matrix expansion in diabetic *C5ar2*^−/−^ mice than in diabetic WT mice (Fig. 2e–g). Notably, in diabetic mice, C5aR2 deficiency resulted in more prominent tubulointerstitial damage than glomerular injury (mesangial matrix expansion), as evidenced by a 92.6% increase in the tubulointerstitial damage index and a 22.7% increase in mesangial matrix expansion upon *C5ar2* knockout (Fig. 2f, g). Transmission electron microscopy (TEM) revealed that both the thickness of the glomerular basement membrane (GBM) and the width of the foot processes were significantly greater in diabetic *C5ar2*^−/−^ mice (Fig. 2h–j). Immunohistochemical staining revealed that tubulointerstitial α-smooth muscle actin (α-SMA) expression was significantly greater in diabetic *C5ar2*^−/−^ mice than in diabetic WT mice (Fig. 2k, l). Additionally, macrophage infiltration in the tubulointerstitium was significantly greater in diabetic *C5ar2*^−/−^ mice than in diabetic WT mice (Fig. 2k, m). qRT-PCR analysis revealed significant upregulation of inflammation- and fibrosis-related genes, including *F4/80*, actin alpha 1 (*Acta1*), transforming growth factor beta 1 (*Tgfb1*), and lipocalin 2 (*Lcn2*) (*P* < 0.05), in diabetic *C5ar2*^−/−^ mice compared with those in diabetic WT mice (Fig. 2n–q).

In vitro experiments in TCMK-1 cells were subsequently performed using a small interfering RNA (siRNA) targeting *C5ar2* (si*C5ar2*). si*C5ar2* effectively reduced the protein expression of C5aR2 by 74.3% (Supplementary Fig. S2a, b). Under HG/HF conditions, *C5ar2* silencing resulted in a significant decrease in cell viability (Supplementary Fig. S2c), and the expression of markers of damage and fibrosis, including *Lcn2*, *Acta1*, and *Tgfb1*, was significantly greater in the si*C5ar2* group than in the siRNA-negative control (siNC) group (Supplementary Fig. S2d–f).

Collectively, these findings indicate that C5aR2 deficiency exacerbates the progression of DKD, resulting in more severe tubulointerstitial injury, inflammation, and fibrosis, suggesting a protective role for C5aR2 in DKD.

### C5aR2 deficiency exacerbates renal lipid deposition, ER stress, and mitochondrial dysfunction in the context of diabetes

**Fig. 3:**
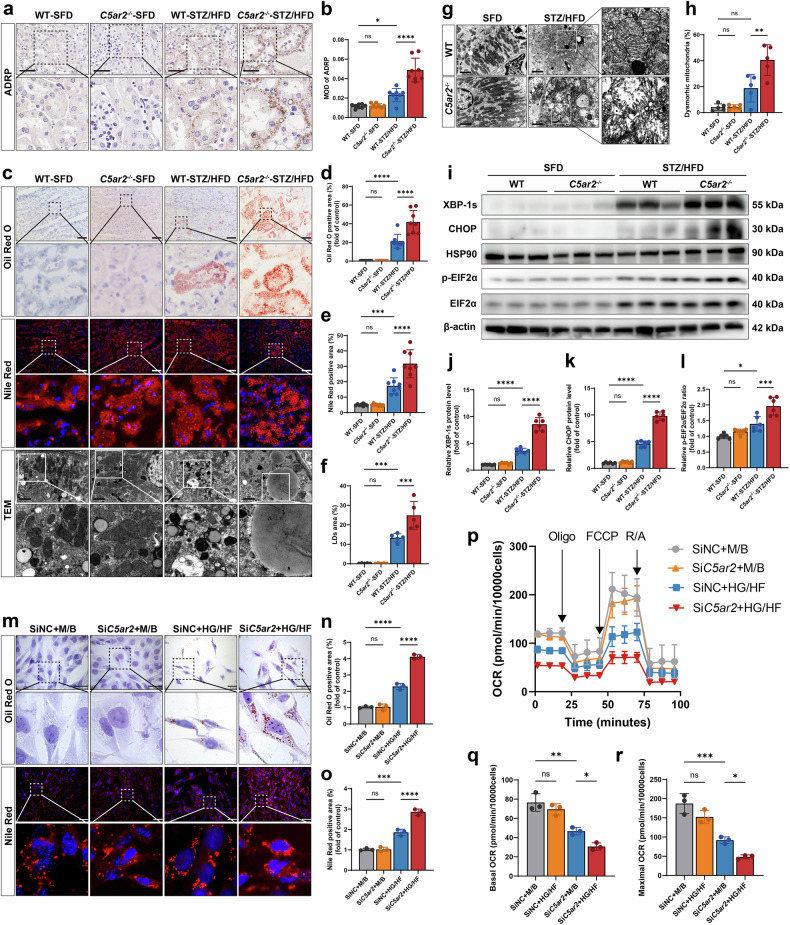
C5aR2 deficiency exacerbated renal lipid deposition, ER stress, and mitochondrial dysfunction in the context of diabetes. **a** Representative IHC images of ADRP staining (scale bars = 60 μm). **b** Quantitative analysis of ADRP staining in different groups of mice (*n* = 8 per group). **c**‒**e** Representative images and quantitative analysis of Oil Red O staining and Nile Red staining in different groups of mice (*n* = 8 per group) (scale bars = 200 μm). **c**, **f** Representative TEM micrographs and quantitative analysis of lipid droplet areas in renal tubular epithelial cells (TECs) from different groups of mice (*n* = 5 per group) (scale bars = 2 µm). **g**, **h** Representative TEM micrographs and quantification of dysmorphic mitochondria in renal TECs from different groups of mice (*n* = 5 per group) (scale bars = 2 µm). **i**–**l** Representative western blotting images and quantitative analysis of ER stress markers, including XBP-1s, p-EIF2α, EIF2α, and CHOP, in different groups of mice (*n* = 6 per group). **m**‒**o** Representative images and quantitative analysis of Oil Red O-stained and Nile Red-stained areas of TCMK-1 cells subjected to different treatments (*n* = 3 independent replicates) (scale bars = 50 µm). **p** The mitochondrial oxygen consumption rates (OCRs) in different groups of TCMK-1 cells were measured via an extracellular flux analyzer. Representative data (*n* = 3 independent replicates) are shown. **q**, **r** Quantified OCR parameters are presented as the mean ± SD. Statistical analysis was performed by one-way ANOVA (Tukey’s multiple comparisons test). ns not significant; *P < 0.05; **P < 0.01; ***P < 0.001; ****P < 0.0001.

Metabolic inflammation, driven by metabolic dysfunction in certain genetic backgrounds, is the central mechanism in the development and progression of DKD^27^. Given that renal inflammation and fibrosis are markedly exacerbated in diabetic *C5ar2*^−/−^ mice, we further investigated whether C5aR2 deficiency exacerbates renal metabolic disturbances. Immunohistochemical staining revealed that the expression of adipose differentiation-related protein (ADRP) was significantly greater in the tubulointerstitium of diabetic *C5ar2*^−/−^ mice than in that of diabetic WT mice (Fig. 3a, b). The intensity of Oil Red O and Nile Red staining in the kidneys of diabetic *C5ar2*^−/−^ mice was significantly greater than that in the kidneys of diabetic WT mice (Fig. 3c–e). TEM revealed a significantly greater number and larger area of lipid droplets in TECs from diabetic *C5ar2*^−/−^ mice than in those from diabetic WT mice (Fig. 3c, f). TEM further revealed a pronounced increase in the number of vacuoles and swollen mitochondria in the TECs of diabetic *C5ar2*^−/−^ mice (Fig. 3g, h). Moreover, western blotting analysis revealed that the expression of ER stress markers, including spliced X box protein-1 (XBP-1s), phospho-eukaryotic translation initiation factor 2α (p-EIF2α), EIF2α, and C/EBP homologous protein (CHOP), was significantly greater in the kidneys of diabetic *C5ar2*^−/−^ mice than in those of diabetic WT mice (Fig. 3i–l).

Next, we investigated the effects of *C5ar2* knockdown on cellular metabolism in vitro. Oil Red O and Nile Red staining revealed that siRNA-mediated knockdown of *C5ar2* expression significantly increased lipid accumulation in TCMK-1 cells under HG/HF conditions (Fig. 3m–o). Following the stimulation of TCMK-1 cells with HG/HF conditions, Seahorse metabolic analysis revealed that both basal and maximal mitochondrial oxygen consumption rates (OCRs) were significantly lower in cells transfected with si*C5ar2* than in those transfected with siNC (Fig. 3p–r).

Collectively, these data suggest that C5aR2 plays a key role in maintaining lipid metabolism and ER and mitochondrial functions in TECs in the context of diabetes.

### C5aR2 deficiency impairs PS biosynthesis in TECs in DKD

**Fig. 4:**
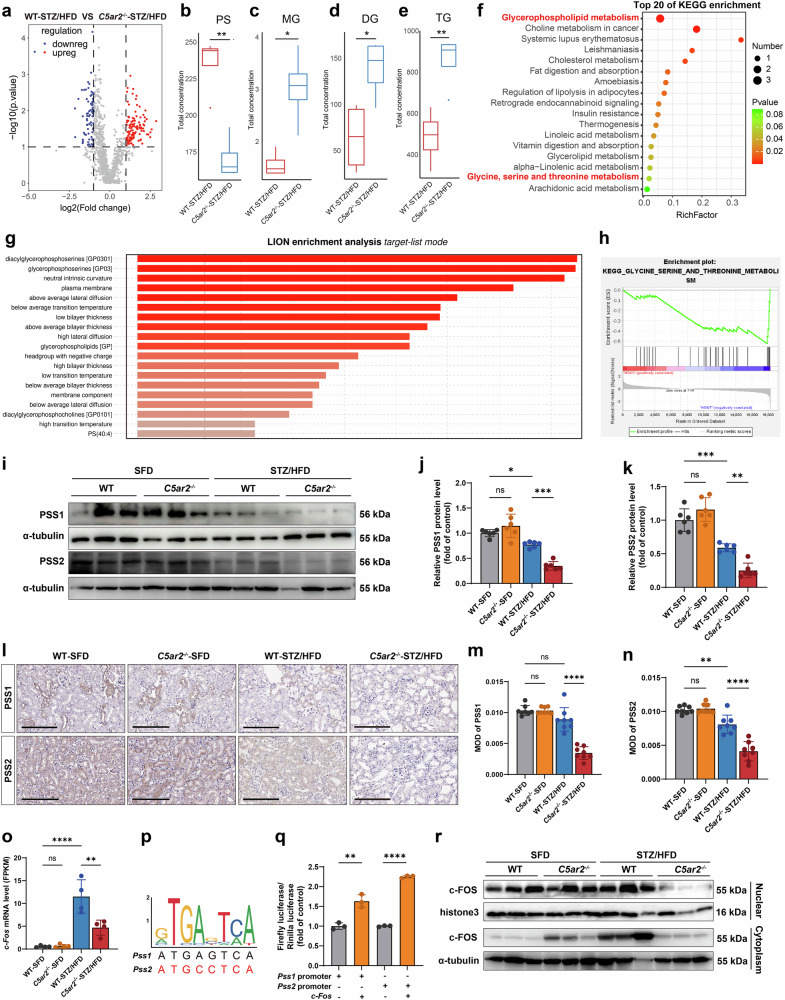
C5aR2 deficiency impaired PS biosynthesis in TECs in the context of diabetes. **a** Volcano plot showing different lipid contents in renal cortex tissues between diabetic *C5ar2*^−/−^ mice and diabetic WT mice (*n* = 4 per group). **b**‒**e** Differential lipid analysis of renal cortex tissues between diabetic *C5ar2*^−/−^ mice and diabetic WT mice (*n* = 4 per group). **f** Analysis of the top 20 enriched Kyoto Encyclopedia of Genes and Genomes (KEGG) pathways in renal cortex tissues between diabetic *C5ar2*^−/−^ mice and diabetic WT mice. **g** Lipid ontology (LION) enrichment analysis of renal cortex tissues from diabetic *C5ar2*^−/−^ mice and diabetic WT mice. **h** Gene set enrichment analysis (GSEA) was performed on transcriptomic data from diabetic *C5ar2*^−/−^ mice and diabetic WT mice. **i**‒**k** Representative western blotting images and quantitative analysis of PSS1 and PSS2 expression in renal cortex tissues from different groups of mice (*n* = 6 per group). **l**‒**n** Representative IHC images and quantitative analysis of PSS1 and PSS2 staining in different groups of mice (*n* = 8 per group) (scale bars = 200 μm). **o** Fragments per kilobase of exon model per million mapped fragments (FPKM) values of *c-Fos* in renal cortex tissue from different groups of mice. **p** Bioinformatic analysis revealed conserved AP-1 binding motifs in both the *Pss1* and *Pss2* promoter regions. **q** Luciferase reporter assays of *c-Fos*-overexpressing TCMK-1 cells demonstrated promoter activation (*n* = 3 independent experiments). **r** Nucleocytoplasmic fractionation and western blotting analysis of c-FOS in TCMK-1 cells subjected to different treatments. Representative results of three independent biological experiments are shown. The data are presented as the means ± SDs. The data were analyzed via unpaired two-tailed Student’s *t*-test (**b**‒**e**, **q**) and two-sided one-way ANOVA with Tukey’s test (**j**, **k**, **m**–**o**). ns not significant; *P < 0.05; **P < 0.01; ***P < 0.001; ****P < 0.0001.

To elucidate the metabolic changes in the kidney triggered by C5aR2 deficiency in DKD, lipidomic analyses and bulk RNA-seq were performed on renal cortex tissues from diabetic *C5ar2*^−/−^ mice and diabetic WT mice. Differential lipid analyses revealed that the relative abundance of total PS in diabetic *C5ar2*^−/−^ mice was significantly lower than that in diabetic WT mice (Fig. 4a, b), which was consistent with the results of previous studies^23^. Beyond PS alterations, we observed substantial remodeling of individual PC and PE species in diabetic *C5ar2*^−/−^ mice. While the total PC and PE levels did not significantly differ (Supplementary Fig. S3a, b), the levels of multiple molecular species markedly differed (Supplementary Table S3). Among the PS species, PS (22:6), PS (20:4), and PS (18:0) showed the most pronounced decreases. Similarly, PC (20:4) and PC (20:5) were the most substantially modified PC species, whereas PE (20:4) and PE (22:6) emerged as the most significantly altered PE species (Supplementary Table S3). We also observed a significant increase in the levels of neutral lipids, such as triacylglycerols (TGs) and diacylglycerols (DGs), in the kidneys of diabetic *C5ar2*^−/−^ mice (Fig. 4c–e). Furthermore, Kyoto Encyclopedia of Genes and Genomes (KEGG) and lipid ontology (LION) enrichment analyses revealed that the glycerophospholipid metabolism pathway and the diacylglycerophosphoserine pathway were significantly downregulated in diabetic *C5ar2*^−/−^ mice compared with those in WT controls (Fig. 4f, g). Moreover, gene set enrichment analysis (GSEA) revealed that serine metabolism-related pathways were significantly downregulated in diabetic *C5ar2*^−/−^ mice compared with those in WT control mice (Fig. 4h). PSS1 and PSS2, two key enzymes responsible for PS biosynthesis, play central roles in regulating PS metabolic homeostasis^24^. Immunohistochemical staining revealed significant downregulation of PSS1 and PSS2 expression in the kidney tissues of DKD patients (Supplementary Fig. S3c–e). Compared with those in diabetic WT mice, the expression of PSS1 and PSS2 in diabetic *C5ar2*^*−/−*^ mice was significantly lower, as confirmed by qRT-PCR, western blotting, and immunohistochemical staining (Supplementary Fig. S3f, g; Fig. 4i–n). In TCMK-1 cells under HG/HF conditions, both the mRNA and protein levels of PSS1 and PSS2 were significantly lower in the si*C5ar2* group than in the siNC group (Supplementary Fig. S3h–l).

Next, to investigate the mechanism by which C5aR2 regulates PSS expression in DKD, we analyzed bulk RNA-seq data from diabetic *C5ar2*^−/−^ mice in comparison with those from diabetic WT controls and identified significantly altered transcription factors. Notably, the expression of the activator protein-1 (AP-1) family member cellular Fos proto-oncogene (c-FOS) was most strongly altered (Supplementary Fig. S4a). c-FOS is reportedly essential for the transcriptional activation of *Pss*^28^. Our transcriptomic results revealed that *c-Fos* mRNA expression was significantly lower in the kidneys of diabetic *C5ar2*^−/−^ mice than in those of their diabetic WT counterparts (Fig. 4o). qRT-PCR analysis also confirmed a significant decrease in *c-Fos* transcript levels in the kidneys of diabetic *C5ar2*^−/−^ mice (Supplementary Fig. S4b). Given that extracellular signal-regulated kinase 1/2 (ERK1/2) signaling activation is a major canonical pathway for c-FOS induction^29^, we investigated its potential involvement in C5aR2-mediated regulation. Our results revealed that genetic deletion of *C5ar2* led to a significant increase in ERK1/2 phosphorylation (Supplementary Fig. S4c), but this change was accompanied by decreased *c-Fos* mRNA levels. This inverse relationship suggests that C5aR2 may regulate c-FOS through a pathway distinct from canonical ERK signaling.

By further analyzing the JASPAR database, we discovered that the transcription factor c-FOS had direct binding sites in the promoter regions of *Pss1* and *Pss2* (Fig. 4p). Dual-luciferase reporter assays revealed that c-FOS overexpression significantly increased the activity of either the *Pss1* or *Pss2* promoter ligated to the GSV238 vector, with the *Pss2* promoter exhibiting obviously stronger activation than the *Pss1* promoter (Fig. 4q). Furthermore, by mutating the *Pss2* promoter sequence and performing chromatin immunoprecipitation (ChIP) experiments, we confirmed that c-FOS promoted the transcription of *Pss2* by directly binding to its promoter region (Supplementary Fig. S4d, e). In addition, western blotting analysis revealed a notable reduction in the nuclear translocation of c-FOS in the kidneys of diabetic *C5ar2*^−/−^ mice compared with those of diabetic WT mice (Fig. 4r). These findings were further supported by the results of in vitro experiments. *C5ar2* knockdown in TCMK-1 cells under HG/HF conditions significantly reduced c-FOS expression at both the mRNA and protein levels (Supplementary Fig. S4f–h). To test whether c-FOS mediates the effects of C5aR2, we overexpressed c-FOS in *C5ar2*-knockdown TCMK-1 cells under HG/HF conditions (Supplementary Fig. S4h). c-FOS overexpression restored the expression of both PSS1 and PSS2 (Supplementary Fig. S4i, j) and ameliorated lipid accumulation (Supplementary Fig. S4k, l). These results confirm that c-FOS is required for the C5aR2-mediated regulation of PSS and lipid homeostasis.

Collectively, these data suggest that in the context of diabetes, C5aR2 deficiency leads to reduced PSS1 and PSS2 expression and disrupted PS biosynthesis, driven by downregulation of the transcription factor c-FOS.

### C5aR2-regulated PSS maintains MAM formation by interacting with MFN2

**Fig. 5:**
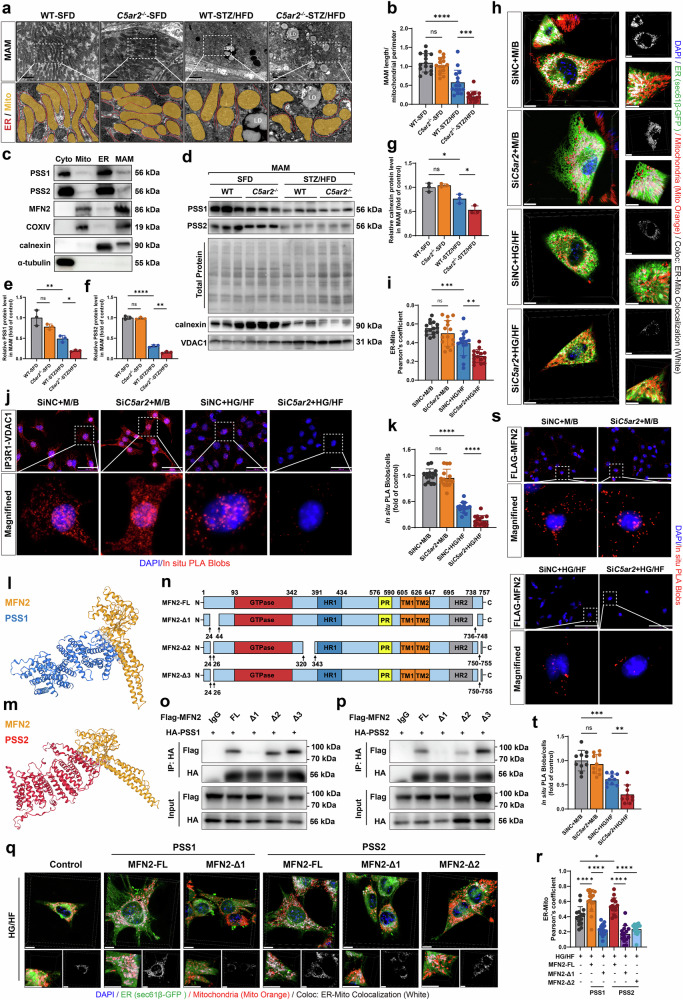
C5aR2-regulated PSS maintained MAM formation by interacting with MFN2. **a** MAM formation in TECs was analyzed via TEM imaging. Scale bars = 2 µm. The ER and mitochondria in the TEM images were graphically reconstructed to visualize MAM formation. Mito: mitochondria (orange), ER (red). **b** Quantification of the MAM length to the mitochondrial perimeter ratio in different groups of WT mice, *n* = 15 (150 mitochondria); *C5ar2*^−/−^ mice, *n* = 15 (193 mitochondria); diabetic WT mice, *n* = 15 (232 mitochondria); and diabetic *C5ar2*^−/−^ mice, *n* = 15 (175 mitochondria) microscopic fields from 5 mice/group. **c** Representative western blotting images of the specified proteins in the cytoplasm, mitochondrial fractions, ER fractions, and MAM fractions in renal cortex tissue from WT mice. **d**–**f** Representative western blotting images and quantitative analysis of PSS1 and PSS2 protein levels in the MAM fraction of renal cortex tissue from different groups of mice (*n* = 3 per group). Total proteins stained with Ponceau S served as an internal reference for quantifying MAM proteins. **d**, **g** Representative western blotting images and quantitative analysis of calnexin and VDAC1 protein levels in the MAM fraction in renal cortex tissues from different groups of mice (*n* = 3 per group). **h** Representative live-cell confocal microscopy images of ER–mitochondria contacts in TCMK-1 cells under different treatments. The ER was labeled with sec61β-GFP (green), the mitochondria were labeled with Mito Orange (red), and the nuclei were labeled with DAPI (blue). Z-stack images with orthogonal projections of the boxed regions are shown (scale bars = 10 µm). **i** Quantification of ER–mitochondria colocalization using Pearson’s correlation coefficient (*n* = 15 microscopic fields from three independent experiments). **j** Representative in situ proximity ligation assay (PLA) images of TCMK-1 cells probed with IP3R1 and VDAC1 antibodies. The red dots indicate colocalization sites between IP3R1 and VDAC1. The cells were counterstained with DAPI (blue) (scale bars = 100 μm). **k** Quantitative analysis of PLA red spot counts in TCMK-1 cells subjected to different treatments. **l** Surface representation of the molecular docking between truncated MFN2 (Protein Data Bank code 6JFK) and PSS1. MFN2 (orange), PSS1 (blue). **m** Surface representation of the molecular docking between truncated MFN2 (6JFK) and PSS2. MFN2 (orange), PSS2 (red). **n** Schematic diagram of the structure of the MFN2 domain and truncation mutants. FL full-length, GTPase GTPase domain, HR1 heptad repeat 1, PR proline-rich domain, TM1/TM2 transmembrane domains 1 and 2, HR2 heptad repeat 2, Δ1/Δ2/Δ3 truncation mutants with the indicated deletions. **o**, **p** Co-IP analysis of PSS1–MFN2 and PSS2–MFN2 interactions using full-length and truncation mutants of MFN2 in HEK293T cells. **q** Representative confocal microscopy images of ER–mitochondria contacts in human kidney cortical proximal tubule epithelial cells (HK-2 cells) transfected with full-length or truncation mutants of MFN2 under HG/HF conditions (scale bars = 10 µm). **r** Quantification of ER–mitochondria colocalization in HK-2 cells (*n* = 15 microscopic fields from three independent experiments). **s** Representative PLA images of Lv*Pss2* (*Flag*)-TCMK-1 cells probed with FLAG and MFN2 antibodies. Red spots indicate colocalization sites between FLAG and MFN2. The cells were counterstained with DAPI (blue) (scale bars = 100 μm). **t** Quantitative analysis of PLA red spot counts in Lv*Pss2*-TCMK-1 cells subjected to different treatments. The data in the graphs are presented as the means ± SD. The data were analyzed by two-sided one-way ANOVA with Tukey’s test. ns not significant; *P < 0.05; **P < 0.01; ***P < 0.001; ****P < 0.0001.

Next, we investigated the mechanism by which C5aR2 deficiency leads to impaired ER and mitochondrial function. Previous studies have shown that PSS1 and PSS2 are highly enriched in MAMs and play a role in regulating ER and mitochondrial function by modulating phospholipid metabolism at this interface^26^. Therefore, we sought to clarify whether C5aR2-regulated PSS is involved in regulating MAM formation. Recent studies have demonstrated marked impairment of MAM formation and function in PTECs from patients with DKD^26^. Consistent with these findings, the number of mitochondria enveloped by the ER was significantly reduced in the TECs of diabetic WT mice, accompanied by a marked decrease in MAM length (Fig. 5a, b). Notably, compared with diabetic WT mice, diabetic *C5ar2*^−/−^ mice presented an even greater reduction in MAM length in TECs (Fig. 5a, b).

To characterize the molecular composition of the MAM fractions, we isolated renal tubules from mice and performed subcellular organelle isolation via ultracentrifugation. The purity of the isolated fractions was validated using compartment-specific markers (Fig. 5c). The MAM fraction contained both the mitochondrial marker cytochrome c oxidase subunit IV (COX IV) and the ER marker calnexin, which is consistent with its identity as a mitochondria–ER contact site. The mitochondrial fraction was highly enriched in COX IV, while the ER fraction was enriched in calnexin. The cytosolic fraction, marked by α-tubulin, showed no contamination. Western blotting analysis revealed markedly lower levels of PSS1 and PSS2 expression in the MAMs of diabetic *C5ar2*^−/−^ mice than in those of diabetic WT mice (Fig. 5d–f). Moreover, compared with those from diabetic WT mice, the MAMs from diabetic *C5ar2*^−/−^ mice contained fewer associated ER membranes (Fig. 5d, g).

To validate these findings in vitro, we employed live-cell confocal microscopy to examine ER–mitochondria contacts in TCMK-1 cells. Using sec61β-GFP to label the ER and Mito Orange for mitochondrial staining, we observed significantly reduced colocalization between these organelles under HG/HF conditions, and *C5ar2* knockdown further exacerbated this disruption. Quantitative colocalization analysis revealed decreased Pearson’s correlation coefficients, and orthogonal projections confirmed the reduced spatial proximity between the ER and mitochondria (Fig. 5h, i). We subsequently conducted a proximity ligation assay (PLA) to evaluate the interaction between inositol 1,4,5-trisphosphate receptor type 1 (IP3R1) and voltage-dependent anion channel 1 (VDAC1), which assesses ER‒mitochondria contact by detecting protein proximity within 40 nm^30,31^. Consistent with our imaging data, the IP3R1–VDAC1 interaction was significantly reduced after HG/HF treatment, and *C5ar2* knockdown further reduced this interaction (Fig. 5j, k). The concordance between these complementary approaches highlights the critical role of C5aR2 in maintaining MAM integrity.

To further explore the mechanism of PSS-mediated MAM formation, we analyzed the subcellular distribution of PSS and MFN2 using our fractionation data in combination with published subcellular proteomic data from HEK293T cells^32^. Both approaches consistently demonstrated that PSS1 and PSS2 were primarily localized to the ER and MAM fractions (Fig. 5c; Supplementary Fig. S5a, b). In contrast, MFN2 was predominantly enriched in both the MAM and mitochondrial fractions but absent from the ER fraction (Fig. 5c; Supplementary Fig. S5c). It was recently reported that MFN2 not only promotes mitochondrial fusion and connects the ER and mitochondrial membranes but also facilitates the translocation of PS to mitochondria for PE biosynthesis^33–35^. Therefore, we further investigated the functional relationship between PSS and MFN2. Western blotting analysis revealed that MFN2 expression was significantly lower in the renal cortex of diabetic *C5ar2*^*−/−*^ mice than in that of diabetic WT mice (Supplementary Fig. S5d, e), and *C5ar2* knockdown significantly reduced MFN2 protein levels in HG/HF-treated TCMK-1 cells (Supplementary Fig. S5f, g). Given that MFN2 depletion has been reported to reduce PSS1 and PSS2 expression in the liver^35^, we tested whether MFN2 also regulates PSS expression in TCMK-1 cells. MFN2 overexpression in HG/HF-treated TCMK-1 cells effectively reversed the decreases in both PSS1 and PSS2 expression (Supplementary Fig. S5h–k), suggesting functional cooperation between MFN2 and PSS in maintaining phospholipid metabolic homeostasis. As PSS catalyzes PS biosynthesis on the ER membrane, whereas MFN2 facilitates its transport to mitochondria, the efficient coupling of these two critical steps likely relies on their direct physical interaction at membrane contact sites. We therefore hypothesized that PSS and MFN2 may directly interact to coordinate MAM formation.

To validate this hypothesis, we first performed molecular docking analyses of the PSS1–MFN2 and PSS2–MFN2 interactions using ZDOCK 3.0. Comprehensive docking revealed stable protein‒protein interaction models for both the PSS1‒MFN2 complex and the PSS2‒MFN2 complex (Fig. 5l, m). To experimentally validate these interactions, we established TCMK-1 cell lines overexpressing Flag-tagged PSS1 (OE-PSS1) and Flag-tagged PSS2 (OE PSS2). Co-immunoprecipitation (co-IP) assays demonstrated that both Flag-tagged PSS1 and PSS2 formed stable immune complexes with endogenous MFN2 (Supplementary Fig. S5l), and reciprocal co-IP experiments confirmed these interactions (Supplementary Fig. S5m), providing direct evidence for PSS1/PSS2–MFN2 complex formation.

To further identify the specific regions of MFN2 required for PSS binding, we generated three truncation mutants of MFN2 and performed co-IP experiments. MFN2-Δ1 (lacking amino acids 24–44 and 736–748) failed to bind either PSS1 or PSS2 (Fig. 5n–p). MFN2-Δ2 (lacking amino acids 24–26, 320–343, and 750–755) retained the ability to bind to PSS1 but lost the ability to interact with PSS2, indicating that the region spanning residues 320–343 is specific to PSS2 (Fig. 5n–p). MFN2-Δ3 (lacking amino acids 24–26 and 750–755) bound both PSS1 and PSS2 normally (Fig. 5n–p). Functionally, only MFN2-FL rescued MAM formation in HG/HF-treated human kidney cortical proximal tubule epithelial cells (HK-2 cells), whereas MFN2-Δ1 and MFN2-Δ2 failed to do so (Fig. 5q, r). These results demonstrate that intact interactions between PSS and MFN2 are essential for MAM formation. Furthermore, to investigate the regulatory role of C5aR2 in maintaining interactions between PSS2 and MFN2, we performed a PLA and observed significantly fewer PSS2–MFN2 interactions in HG/HF-treated TCMK-1 cells transfected with si*C5ar2* than in those transfected with siNC (Fig. 5s, t).

Collectively, these data suggest that C5aR2-regulated PSS interacts with MFN2 on the ER to mediate mitochondrial–ER contact.

### PSS2 overexpression ameliorates C5aR2 deficiency-induced impairment of MAM formation and PS biosynthesis

**Fig. 6:**
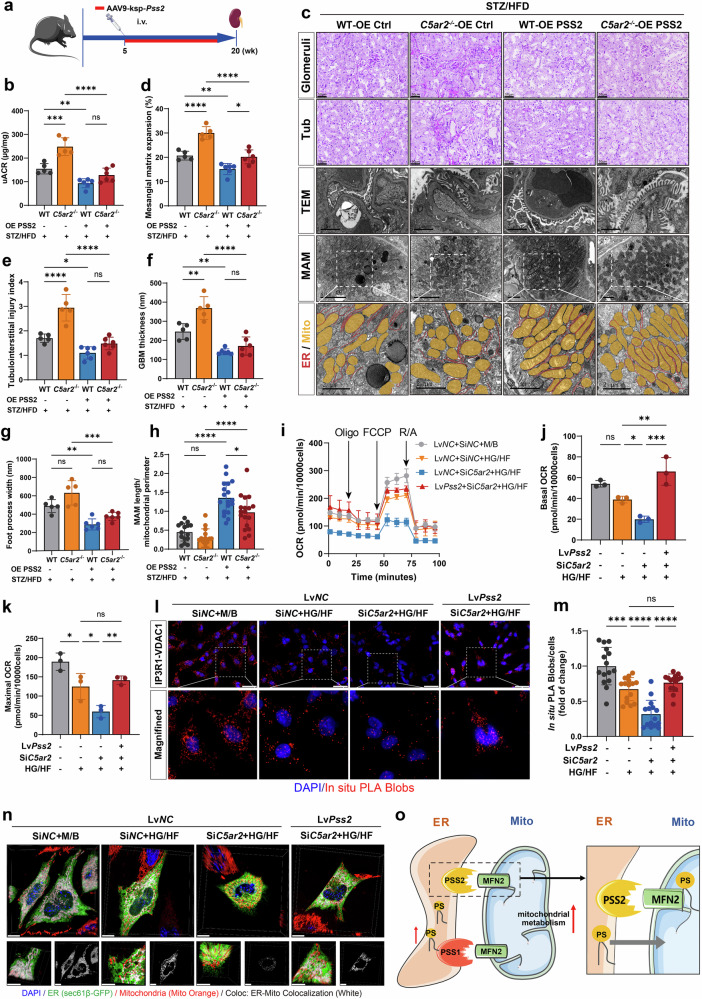
PSS2 overexpression mitigated the impairment of MAM formation and PS biosynthesis induced by C5aR2 deficiency. **a** Experimental scheme for assessing PSS2 overexpression. AAV9-Ksp-*Pss2* was administered via tail vein (i.v.) injection at week 5, and the mice were sacrificed at week 20 for analysis (*n* = 6 per group). **b** uACRs in different groups of mice (*n* = 6 per group). **c** Representative images of PAS staining of the glomeruli and tubulointerstitium (scale bars = 50 µm); representative TEM micrographs (scale bars = 2 µm). MAM formation was analyzed via TEM imaging in TECs (scale bars = 5 µm). The ER and mitochondria in the TEM images were graphically reconstructed to visualize MAM formation. Mito (orange) and ER (red). **d**, **e** Quantitative analysis of mesangial matrix expansion (**d**) and the tubulointerstitial injury index (**e**) in different groups of mice. **f**, **g** Quantification of the GBM thickness (**f**) and foot process width (**g**) in different groups of mice (*n* = 6 per group). **h** Quantification of the MAM length to mitochondrial perimeter ratio in different groups of mice: STZ/HFD-WT + OE Ctrl mice, *n* = 15 (with 167 mitochondria); STZ/HFD-*C5ar2*^−/−^ + OE Ctrl mice, *n* = 15 (with 272 mitochondria) microscopic fields from 5 mice/group; STZ/HFD-WT + OE PSS2 mice, *n* = 15 (with 193 mitochondria); STZ/HFD-*C5ar2*^−/−^ + OE PSS2 mice, *n* = 15 (with 220 mitochondria) microscopic fields from 6 mice/group. **i** The mitochondrial OCRs of LvNC and Lv*Pss2*-TCMK-1 cells were analyzed in the presence or absence of HG/HF or *C5ar2* knockdown. **j**, **k** Representative OCR tracings (from three independent experiments) and quantified OCR values are presented. **l** Representative PLA images of TCMK-1 cells probed with IP3R1 and VDAC1 antibodies. The red spots indicate sites of IP3R1 and VDAC1 colocalization. The cells were counterstained with DAPI (blue) (scale bars = 50 μm). **m** Quantitative analysis of PLA red spot counts in TCMK-1 cells subjected to different treatments. **n** Representative live-cell confocal microscopy images of ER–mitochondria contacts in TCMK-1 cells under different treatments. The ER was labeled with sec61β-GFP (green), the mitochondria were labeled with Mito Orange (red), and the nuclei were labeled with DAPI (blue). Z-stack images with orthogonal projections of the boxed regions are shown (scale bars = 5 µm). **o** Working model of how PSS interacts with MFN2 at the mitochondria–ER interface to maintain MAM formation and PS homeostasis. The data in the graphs are presented as the means ± SDs. The data were analyzed by two-sided one-way ANOVA with Tukey’s test. ns not significant; *P < 0.05; **P < 0.01; ***P < 0.001; ****P < 0.0001.

Given that c-FOS activates the *Pss2* promoter more potently than it does the *Pss1* promoter, we focused on the role of PSS2 in C5aR2-mediated mitochondria–ER contact. We administered adeno-associated virus serotype 9 (AAV9) vectors carrying *Pss2* under the kidney-specific cadherin (Ksp) promoter (AAV9-Ksp-*Pss2*, PSS2 overexpression (OE)) or control vector (AAV9-Ksp-Ctrl, OE Ctrl) to diabetic *C5ar2*^*−/−*^ mice via tail vein injection (Fig. 6a) and confirmed successful overexpression by western blotting assay (Supplementary Fig. S6a). PSS2 overexpression significantly reduced the uACR in diabetic *C5ar2*^−/−^ mice (Fig. 6b) but did not significantly affect fasting blood glucose, blood triglyceride, or blood cholesterol levels (Supplementary Table S4). PAS staining revealed that PSS2 overexpression markedly attenuated tubulointerstitial damage and mesangial matrix expansion in diabetic *C5ar2*^−/−^ mice (Fig. 6c–e). TEM revealed significant reductions in both the GBM thickness and foot process width in OE PSS2-diabetic *C5ar2*^−/−^ mice compared with those in OE Ctrl-diabetic *C5ar2*^−/−^ mice (Fig. 6c, f, g). qRT-PCR analysis further demonstrated the downregulation of proinflammatory and fibrotic genes (Supplementary Fig. S6b–d). Additionally, TEM and Western blotting revealed reduced lipid droplet accumulation and decreased ER stress marker expression in OE PSS2 mice (Supplementary Fig. S6e–j). Importantly, PSS2 overexpression significantly increased the MAM length in TECs (Fig. 6c, h).

These findings were corroborated in vitro. In HG/HF-treated TCMK-1 cells with *C5ar2* knockdown, compared with the control vector (LvCtrl), lentivirus-mediated PSS2 overexpression (Lv*Pss2*) attenuated the expression of injury and fibrosis markers (Supplementary Fig. S6k–n). Furthermore, PSS2 overexpression improved mitochondrial function, as evidenced by significantly increased basal and maximal OCRs (Fig. 6i–k) and reduced lipid deposition (Supplementary Fig. S6o, p). PLA showed an enhanced IP3R1–VDAC1 interaction (Fig. 6l, m), and live-cell confocal microscopy confirmed improved ER–mitochondria colocalization in PSS2-overexpressing cells compared with that in control cells (Fig. 6n; Supplementary Fig. S6q).

Since PS is an essential component of the MAM^36^, we investigated whether the expression of PSS affects the levels of PS at ER‒mitochondria contact sites. Enzyme-linked immunosorbent assays (ELISAs) of the MAM fractions revealed significantly lower PS levels in diabetic *C5ar2*^−/−^ mice than in diabetic WT controls (Supplementary Fig. S7a). Importantly, PSS2 overexpression restored the PS content within the MAM (Supplementary Fig. S7b).

As illustrated in Fig. 6o, these findings collectively demonstrate that PSS2 overexpression ameliorates C5aR2 deficiency-induced impairment of MAM formation and PS biosynthesis under diabetic conditions.

### The C5aR2-specific agonist P59 attenuates lipid accumulation, ER stress, mitochondrial dysfunction, and tubulointerstitial injury in DKD

**Fig. 7:**
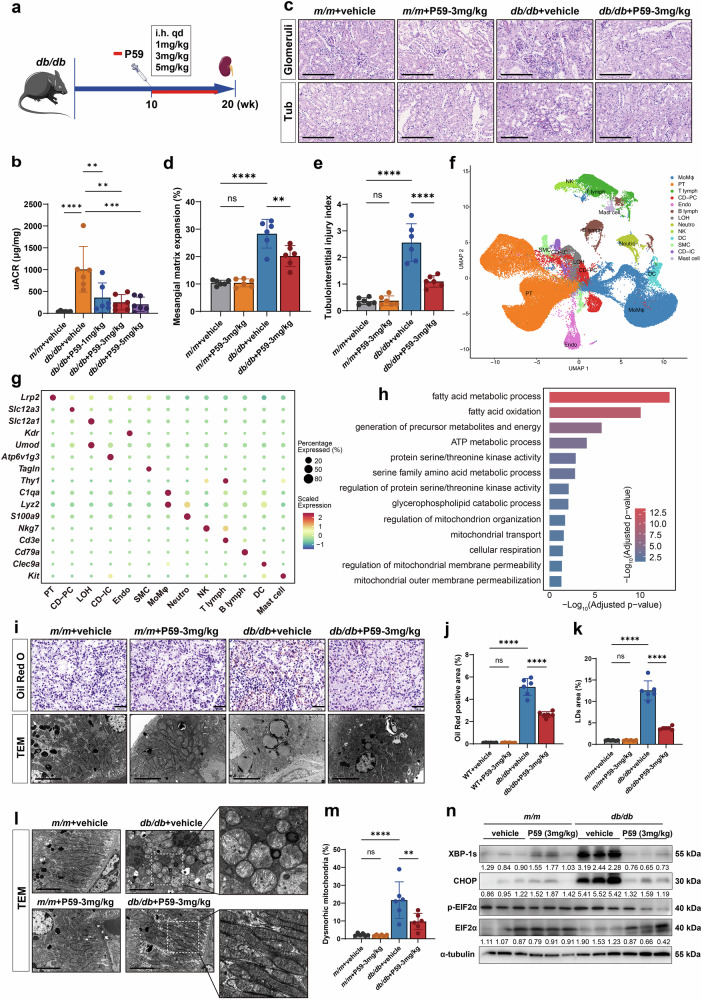
The C5aR2-specific agonist P59 alleviated lipid accumulation, ER stress, mitochondrial dysfunction, and tubulointerstitial injury in *db/db* mice. **a** Schematic illustration of the experimental design for establishing a dose gradient of P59 administered via subcutaneous injection in *db/db* mice. **b** uACR in different groups of mice (*n* = 6 per group). **c** Representative images of PAS staining of the glomeruli and tubulointerstitium (scale bars = 200 μm). **d**, **e** Quantitative analysis of mesangial matrix expansion (**d**) and the tubulointerstitial injury index (**e**) in different groups of mice (*n* = 6 per group). **f** UMAP plot showing thirteen populations of kidney cells in vehicle-treated *m/m* mice, vehicle-treated *db/db* mice, and P59-treated *db/db* mice (*n* = 3 per group). Each dot corresponds to a single cell and is colored according to the cell type. **g** Dot plot showing the expression of genes characteristic of each cell population in the scRNA-seq data. **h** GO enrichment analysis of DEGs between vehicle-treated *db/db* mice and P59-treated *db/db* mice. -Log_10_ (adjusted *P* value) > 1.3 was used as the cutoff. **i**, **j** Representative images and quantitative analysis of Oil Red O-stained areas in different groups of mice (*n* = 6 per group*)*. Scale bars = 100 µm. **i**, **k** Representative TEM micrographs and quantitative analysis of LD areas in TECs from different groups of mice (*n* = 6 per group) (scale bars = 5 µm). **l**, **m** Representative TEM micrographs and quantification of the number of dysmorphic mitochondria in TECs in different groups of mice (*n* = 6 per group) (scale bars = 5 µm). **n** Representative western blotting images and quantitative analysis of ER stress markers (XBP-1s, p-EIF2α, EIF2α, and CHOP) in different groups of mice (*n* = 6 per group*)*. The data in the graphs are presented as the means ± SD. The data were analyzed by two-sided one-way ANOVA with Tukey’s test. ns not significant; **P < 0.01; ***P < 0.001; ****P < 0.0001.

These results indicate that C5aR2 is crucial for the homeostasis of energy metabolism in TECs in DKD. Therefore, we aimed to explore the potential of C5aR2 agonism as a therapeutic strategy for DKD. The C5aR2-specific agonist P59 has been shown to inhibit C5a-induced neutrophil mobilization in a C5aR2-dependent manner without affecting C3aR or C5aR1^37^. To investigate whether P59 has a therapeutic effect on DKD, we randomly assigned *db/db* mice to treatment groups receiving different doses of P59 (1 mg/kg, 3 mg/kg, and 5 mg/kg) administered continuously for 10 consecutive weeks (Fig. 7a). First, we observed a significant reduction in body weight and blood triglyceride levels in *db/db* mice treated with varying doses of P59 compared with those in vehicle-treated *db/db* mice (Supplementary Fig. S8a, b), whereas no significant difference in fasting blood glucose or blood cholesterol levels was detected (Supplementary Tables S5 and S6). Compared with vehicle-treated *db/db* mice, those in the moderate-dose (3 mg/kg) and high-dose (5 mg/kg) P59 treatment groups presented a significant decrease in the uACR (Fig. 7b). Moreover, P59 treatment significantly improved renal histopathology, especially tubulointerstitial damage (Supplementary Fig. S8c, d). qRT-PCR analysis revealed significantly lower expression of *Lcn2*, *Acta1*, *Tgfb1*, and *F4/80* in P59-treated *db/db* mice than in vehicle-treated *db/db* mice (Supplementary Fig. S8e–h).

P59 at a dose of 3 mg/kg was selected for subsequent experiments, since it was the minimum dose used in our dose gradient to achieve significant therapeutic effects. PAS staining revealed that P59 significantly ameliorated tubulointerstitial damage and mesangial matrix expansion in *db/db* mice (Fig. 7c–e). Notably, compared with the control treatment, P59 treatment resulted in a 61.5% reduction in the tubulointerstitial damage index and a 33.4% reduction in mesangial matrix expansion, indicating a preferential therapeutic effect on the tubulointerstitium. TEM revealed that both the GBM thickness and the podocyte foot process width were significantly lower in P59-treated *db/db* mice than in vehicle-treated *db/db* mice (Supplementary Fig. S8i–k). Immunohistochemical staining revealed that P59 significantly decreased tubulointerstitial F4/80 and TGF-β expression in *db/db* mice (Supplementary Fig. S8l–n). These results indicate that the C5aR2 agonist P59 significantly ameliorated renal tubulointerstitial injury in *db/db* mice.

To better understand the effect of C5aR2 activation by P59 on PTECs, we performed scRNA-seq analysis on kidney cells isolated from vehicle-treated *m/m* mice, vehicle-treated *db/db* mice, and P59-treated *db/db* mice (*n* = 3 per group; WT_vehicle, DKD_vehicle, and DKD_P59). Our analysis included 73,763 high-quality renal cells that were segregated into thirteen distinct populations through unsupervised graph-based uniform manifold approximation and projection (UMAP) clustering (Fig. 7f). These populations were annotated via the expression of established renal markers, including proximal tubule cells (PT), collecting duct principal cells (CD_PC), collecting duct intercalated cells (CD_IC), loop of Henle cells (LOH), endothelial cells (Endo), monocytes and macrophages (MoMφ), dendritic cells (DC), neutrophils (Neutro), natural killer cells (NK), T lymphocytes (T_lymph), B lymphocytes (B_lymph), smooth muscle cells (SMC) and mast cells (Fig. 7g).

To elucidate P59-induced transcriptional reprogramming, we analyzed differentially expressed genes (DEGs) in PT cells from P59-treated *db/db* mice and vehicle-treated *db/db* mice. Gene Ontology (GO) enrichment revealed significant upregulation of pathways related to glycerophospholipid metabolism and mitochondrial membrane organization in PT cells from the kidneys of P59-treated *db/db* mice (Fig. 7h). We further verified these results through a series of experiments. Oil Red O staining revealed a significant decrease in lipid deposition in the tubulointerstitium of P59-treated *db/db* mice compared with that in the tubulointerstitium of vehicle-treated *db/db* mice (Fig. 7i, j). TEM revealed that the lipid droplet area in TECs was significantly smaller in P59-treated *db/db* mice than in vehicle-treated *db/db* mice (Fig. 7i, k). TEM further revealed a notable reduction in the number of vacuoles and swollen mitochondria in TECs from P59-treated *db/db* mice compared with those from vehicle-treated *db/db* mice (Fig. 7l, m). Moreover, western blotting analysis revealed that the ER stress markers XBP-1s, p-EIF2α, EIF2α, and CHOP were significantly downregulated in P59-treated *db/db* mice compared with those in vehicle-treated *db/db* mice (Fig. 7n).

In in vitro experiments, under HG/HF conditions, treating TCMK-1 cells with a gradient of P59 concentrations (0, 0.1 μM, 1 μM, 10 μM, 100 μM, and 1000 μM) significantly improved cell viability at concentrations of 100 μM and 1000 μM (Supplementary Fig. S8o). Therefore, a P59 concentration of 100 μM was selected for further studies. qRT-PCR analysis revealed that compared with vehicle treatment, P59 treatment significantly reduced *Tgfb1*, *Acta1*, and *Lcn2* mRNA levels (Supplementary Fig. S8p–r). Compared with those in the vehicle-treated group, both the basal and maximal mitochondrial OCRs were significantly restored in the P59-treated group (Supplementary Fig. S9a–c). Additionally, Oil Red O staining and Nile Red staining demonstrated a significant reduction in lipid deposition in the P59-treated group compared with that in the vehicle-treated group (Supplementary Fig. S9d–f). To confirm P59 specificity, we treated primary renal tubular epithelial cells (PRTECs) isolated from wild-type and *C5ar2*^−/−^ mice with P59 under HG/HF conditions. qRT-PCR analysis revealed that P59 significantly reduced the expression of injury and fibrosis markers (*Lcn2*, *Acta1*, and *Tgfb1*) in wild-type PRTECs, whereas these effects were completely abolished in *C5ar2*^−/−^ PRTECs (Supplementary Fig. S9g–k).

Collectively, these results demonstrate that the C5aR2-specific agonist P59 attenuates lipid accumulation, ER stress, and mitochondrial dysfunction, thereby ameliorating tubulointerstitial injury in DKD.

### The C5aR2 agonist P59 ameliorates DKD by promoting MAM formation and PS biosynthesis mediated by the PSS–MFN2 interaction

**Fig. 8:**
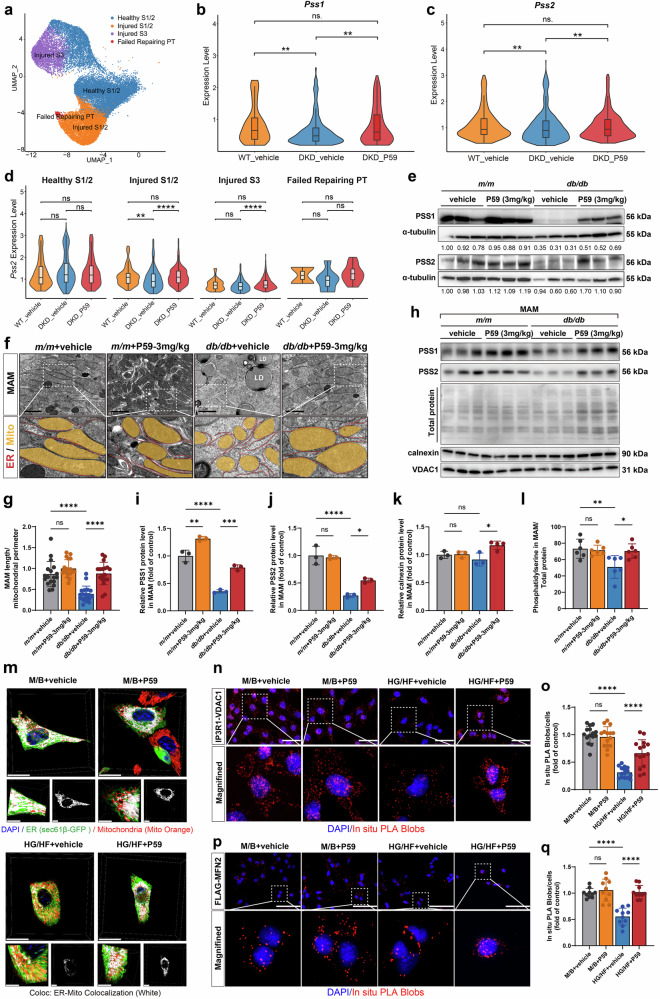
P59 alleviated tubulointerstitial injury in DKD by promoting MAM formation and PS biosynthesis mediated by the interaction between PSS and MFN2. **a** UMAP plot showing five subpopulations of PT cells in vehicle-treated *m/m* mice, vehicle-treated *db/db* mice, and P59-treated *db/db* mice (*n* = 3 per group). Each dot corresponds to a single cell and is colored according to the cell type. **b**, **c** Violin plots showing *Pss1* and *Pss2* transcript expression levels of the indicated genes in PT cells from vehicle-treated *m/m* mice, vehicle-treated *db/db* mice, and P59-treated *db/db* mice. **d** Expression levels of *Pss2* in each PT cluster revealed by scRNA-seq. **e** Representative western blotting images and quantitative analysis of PSS1 and PSS2 expression in renal cortex tissues from different groups of mice (*n* = 6 per group). **f** MAM formation in TECs was analyzed via TEM imaging (scale bars = 2 µm). The ER and mitochondria in TEM images were graphically reconstructed to visualize MAM formation. Mito (orange) and ER (yellow). **g** Quantification of the MAM length to mitochondrial perimeter ratio in different groups of mice; *m/m* + vehicle group, *n* = 15 (with 261 mitochondria); *m/m* + P59 (3 mg/kg) group, *n* = 15 (with 270 mitochondria); *db/db* + vehicle group, *n* = 15 (with 192 mitochondria); *db/db* + P59 (3 mg/kg) group, *n* = 15 (with 235 mitochondria) microscopic fields from 6 mice/group. **h**–**j** Representative western blotting images and quantitative analysis of PSS1 and PSS2 protein levels in the MAM fraction in renal cortex tissues from different groups of mice (*n* = 3 per group). Total proteins stained with Ponceau S served as an internal reference for quantifying MAM proteins. **h**, **k** Representative western blotting images and quantitative analysis of calnexin and VDAC1 protein levels in the MAM fraction in renal cortex tissues from different groups of mice (*n* = 4 in the *db/db* + P59-3 mg/kg group; *n* = 3 in the other groups). **l** ELISA analysis of PS levels in the MAM fraction of renal tubules in different groups of mice (*n* = 6 per group). **m** Representative live-cell confocal microscopy images of ER–mitochondria contacts in TCMK-1 cells under different treatments. The ER was labeled with sec61β-GFP (green), the mitochondria were labeled with Mito Orange (red), and the nuclei were labeled with DAPI (blue). Z-stack images with orthogonal projections of the boxed regions are shown (scale bars = 10 µm). **n** Representative PLA images of TCMK-1 cells probed with IP3R1 and VDAC1 antibodies. The red spots indicate sites of IP3R1 and VDAC1 colocalization. The cells were counterstained with DAPI (blue) (scale bars = 100 μm). **o** Quantitative analysis of PLA red spot counts in TCMK-1 cells subjected to different treatments. **p** Representative PLA images of Lv*Pss2* (*Flag*)-TCMK-1 cells probed with FLAG and MFN2 antibodies. Red spots indicate colocalization sites between FLAG and MFN2. The cells were counterstained with DAPI (blue) (scale bars = 100 μm). **q** Quantitative analysis of PLA red spot counts in Lv*Pss2* (*Flag*)-TCMK-1 cells subjected to different treatments. The data in the graphs are presented as the means ± SD. The data were analyzed by two-sided one-way ANOVA with Tukey’s test. ns not significant; *P < 0.05; **P < 0.01; ***P < 0.001; ****P < 0.0001.

To further validate our findings that C5aR2 improved mitochondrial and ER function by modulating PSS expression in PTECs, we further subdivided PT cells into subpopulations and identified four functionally distinct subpopulations: healthy S1/2, injured S1/2, injured S3, and failed-repair PT cells (Fig. 8a). The defined marker expression profiles for these subpopulations are schematically summarized in Supplementary Fig. S10a. Consistent with our above findings, we observed significant downregulation of both *Pss1* and *Pss2* expression in PT cells from *db/db* mice compared with PT cells from *m/m* mice, and this downregulation was markedly reversed by P59 treatment (Fig. 8b, c). Subpopulation analysis revealed that P59-induced upregulation of *Pss2* was more pronounced than that of *Pss1*, with the greatest response observed in the injured S1/2 population (Fig. 8d), which is consistent with the preferential activation of the *Pss2* promoter by c-FOS. Moreover, both the mRNA and protein levels of PSS1 and PSS2 were significantly greater in P59-treated *db/db* mice than in vehicle-treated *db/db* mice, as determined by qRT-PCR, western blotting, and IHC (Fig. 8e; Supplementary Fig. S10b–f). We detected significant increases in the expression and nuclear translocation of the transcription factor c-FOS in P59-treated *db/db* mice compared with those in vehicle-treated *db/db* mice, further confirming the transcriptional regulatory mechanism of PSS1 and PSS2 (Supplementary Fig. S10g). Notably, this P59-induced increase in c-FOS expression was accompanied by a concurrent decrease in ERK1/2 phosphorylation. This inverse relationship, which is consistent with the phenotype observed in *C5ar2* knockout mice, suggests that C5aR2 may regulate c-FOS through a pathway distinct from canonical ERK1/2 signaling (Supplementary Fig. S10h).

Next, we verified the mechanism by which P59-mediated C5aR2 activation enhances MAM integrity through the upregulation of PSS in DKD. TEM revealed significant improvements in MAM formation in the TECs of *db/db* mice treated with P59 (Fig. 8f, g). Moreover, subcellular fractionation of renal tubules revealed that the protein levels of both PSS1 and PSS2 in the MAM fraction were significantly greater in P59-treated *db/db* mice than in vehicle-treated *db/db* mice (Fig. 8h–j). Furthermore, the MAMs isolated from the renal tubules of P59-treated *db/db* mice contained greater amounts of associated ER membranes, along with a notable increase in the PS content (Fig. 8h, k, l). In HG/HF-treated TCMK-1 cells, qRT-PCR and western blotting confirmed that both PSS1 and PSS2 were significantly upregulated in the P59-treated group compared with the vehicle-treated group (Supplementary Fig. S10i–m). To further confirm P59 specificity, we treated PRTECs isolated from wild-type and *C5ar2*^−/−^ mice with P59 under HG/HF conditions. Western blotting analysis demonstrated that the P59-induced upregulation of PSS1 and PSS2 protein expression was abolished in *C5ar2*^−/−^ PRTECs (Supplementary Fig. S10n, p), confirming that P59 acts specifically through C5aR2. Moreover, live-cell confocal microscopy revealed that P59 treatment significantly enhanced ER–mitochondria colocalization in TCMK-1 cells (Fig. 8m). Additionally, in situ PLA revealed a significant increase in the IP3R1–VDAC1 interaction in the P59-treated group (Fig. 8n, o), suggesting that C5aR2 agonism promoted the formation of MAMs in TECs. Notably, compared with vehicle treatment, P59 treatment significantly increased MFN2 protein levels in the renal cortex of *db/db* mice (Supplementary Fig. S10q), and P59 treatment increased MFN2 expression in HG/HF-treated TCMK-1 cells (Supplementary Fig. S10r). Moreover, PLA revealed that P59 significantly enhanced the PSS2–MFN2 interaction in HG/HF-treated TCMK-1 cells (Fig. 8p, q). To further confirm that the protective effects of P59 depend on PSS, we knocked down both *Pss1* and *Pss2* in P59-treated TCMK-1 cells under HG/HF conditions and found that the P59-induced reduction in lipid accumulation was significantly reversed (Supplementary Fig. S10s, t).

Collectively, these findings suggest that P59 improves MAM formation and PS biosynthesis mediated by the interaction between PSS and MFN2, thereby ameliorating renal tubulointerstitial damage and providing potential therapeutic benefits for DKD.

## Discussion

Cumulating evidence has demonstrated that the complement system plays a key role in the pathogenesis of DKD, particularly *via* the anaphylatoxin C3a and C5a axes^2–4^. Given that some anaphylatoxin-targeting medications have been shown to have therapeutic effects on various inflammatory diseases, such as paroxysmal nocturnal hemoglobinuria (PNH), antineutrophil cytoplasmic antibody (ANCA)-associated vasculitis (AAV), and IgA nephropathy (IgAN), complement inhibition is emerging as an attractive therapeutic target for DKD^7^. Preclinical studies have demonstrated that C5aR1 deletion or pharmacological inhibition of C5aR1 by PMX53 can mitigate proteinuria and tubulointerstitial injury in mouse models of DKD^3^. However, the roles of C5aR2, which is historically regarded as a “decoy” receptor for C5a and is associated with considerably inconsistent findings in various inflammatory diseases^12,15,38^, in DKD remain poorly characterized. In the present study, we identified a significant role for C5aR2 in the development of DKD, elucidated its underlying molecular mechanism, and, for the first time, demonstrated the therapeutic potential of pharmacological activation of C5aR2 by P59 in DKD.

We found that C5aR2 expression was significantly increased in the renal tubulointerstitium and correlated with both disease severity and adverse renal outcomes in DKD patients. Diabetic *C5ar2*^−/−^ mice exhibited significantly aggravated renal phenotypes, particularly tubulointerstitial injuries, which may partly explain the unsatisfactory therapeutic effect of C5a blockade by NOX-D21 in DKD^17^. Conversely, the C5aR2 agonist P59 significantly ameliorated renal pathology. These findings suggest that C5aR2 plays a protective role in DKD and that its upregulation in the diabetic kidney may represent a compensatory response to mitigate disease progression. Mechanistically, our study focused primarily on the metabolic regulatory function of C5aR2 in PTECs and revealed that C5aR2 activation enhances mitochondrial function, restores ER homeostasis, and preserves MAM integrity. In addition to its function in tubular epithelial cells, C5aR2 is expressed in myeloid cells, particularly macrophages, where it has been shown to suppress proinflammatory responses^12,39^. Consistent with these findings, *C5ar2*^−/−^ mice exhibited increased tubulointerstitial macrophage infiltration, which was attenuated by P59 treatment. Therefore, C5aR2 agonism may exert renoprotective effects through dual actions in both TECs and myeloid cells.

In line with our findings, previous studies on chronic metabolic inflammatory diseases, such as obesity, revealed that C5aR2 deficiency significantly exacerbated inflammatory infiltration and metabolic disorders^40^. However, conflicting findings concerning the role of C5aR2 in different kidney diseases have been reported. In pyelonephritis and renal ischemia–reperfusion injury, deletion of C5aR2 attenuated renal inflammation and tissue injury by downregulating high-mobility group box 1 (HMGB1) and nucleotide-binding oligomerization domain-like receptor pyrin domain-containing 3 (NLRP3) inflammasome activation in macrophages^41,42^, whereas in ANCA-associated glomerulonephritis, C5aR2 deficiency aggravated renal injury by promoting neutrophil inflammatory responses^43^. These findings suggest that the role of C5aR2 in renal diseases is context-dependent and varies with the underlying pathophysiology and the predominant cell types involved.

To further elucidate the protective role of C5aR2 in DKD, we investigated its involvement in the regulation of MAMs. In a persistent hyperglycemic state, mitochondrial oxidative phosphorylation is impaired, and excessive ROS are generated, ultimately leading to mitochondrial dysfunction and apoptosis^44^. TECs, one of the most mitochondria-rich cell types, exhibit extensive MAM formation through close contact between mitochondria and the ER^24^. The MAM serves as a central hub of phospholipid transport and metabolism^24^. Here, we demonstrated that C5aR2-mediated upregulation of PSS expression in TECs enhanced phospholipid biosynthesis and transport, thereby improving both ER and mitochondrial function through the regulation of MAM formation. Our in vivo study revealed that compared with diabetic WT mice, diabetic *C5ar2*^−/−^ mice exhibited impaired MAM formation, reduced PS biosynthesis, increased lipid accumulation, and exacerbated mitochondrial dysfunction and ER stress. These findings suggest that C5aR2 is critical for MAM formation and PS biosynthesis. Moreover, PSS2 overexpression significantly reversed the phenotypic consequences of C5aR2 deficiency. Our findings are consistent with previous observations that MAM integrity is significantly reduced in TECs from DKD patients and is correlated with renal injury severity^26^. Furthermore, Yang et al. reported that *Pss* knockdown in *Drosophila* resulted in lipid accumulation and mitochondrial dysfunction^25^, supporting our finding that PSS plays a key role in maintaining MAM integrity and metabolic homeostasis. Taken together, our data indicate that C5aR2 promotes MAM formation and PS biosynthesis by upregulating PSS, thereby alleviating TEC injury in diabetes.

A previous study revealed that MFN2 in hepatocytes can bind PS and transfer it to the mitochondria as a substrate for the respiratory chain and that MFN2 deficiency leads to massive lipid accumulation in the liver and hepatocellular carcinoma^35^. The mechanisms by which MAMs regulate PS transport remain unclear. In the present study, we provide novel insights into this point by revealing that C5aR2 upregulated PSS expression, thereby promoting the PSS–MFN2 interaction. This interaction supports the maintenance of mitochondria–ER membrane contacts and facilitates the assembly of a PS transport complex, thereby enhancing phospholipid metabolic homeostasis in TECs. Given that PS is also a key structural component of MAMs^36^, our results further revealed that the downregulation of PSS expression significantly reduced PS levels in the structural components of MAMs. This reduction in MAM-associated PS may further compromise MAM integrity, creating a vicious cycle that exacerbates lipid metabolism disorders and impairs both ER and mitochondrial function. This may represent a key mechanism by which C5aR2 deficiency induces ER and mitochondrial dysfunction in DKD.

Our study revealed that C5aR2 deficiency suppressed *c-Fos* transcription and nuclear translocation, whereas C5aR2 activation by P59 markedly upregulated *c-Fos* transcription. ERK1/2 activation is a major canonical pathway for c-FOS induction^29^. However, we observed an inverse pattern of ERK1/2 phosphorylation, with increased levels in C5aR2-deficient mice but decreased levels upon P59 treatment. This dissociation between ERK1/2 activity and c-FOS expression suggests that C5aR2 may regulate c-FOS through a non-canonical pathway. Recent studies have shown that C5aR2 can recruit β-arrestin to form signaling complexes independent of its role as a decoy receptor^12^. Furthermore, activation of the GPCR delta-opioid receptor (DOR) has been shown to induce β-arrestin nuclear translocation and stimulate β-arrestin-dependent transcription of *c-Fos*^45^. Taken together, these findings suggest that C5aR2 may promote *c-Fos* transcription through the recruitment of β-arrestin. Upon activation, c-FOS directly enhances *Pss* transcription, which is consistent with its established role in regulating glycerophospholipid biosynthesis^28^. Collectively, our data suggest a C5aR2-β-arrestin-c-FOS-PSS signaling axis that modulates phospholipid metabolism in TECs.

To further evaluate the therapeutic potential of selective C5aR2 agonism, we treated *db/db* mice with P59 and observed significant protection against DKD, as evidenced by a markedly reduced uACR, attenuated tubulointerstitial injury, and improved ER and mitochondrial function, along with enhanced MAM formation. Furthermore, simultaneous knockdown of both *Pss1* and *Pss2* abolished the protective effects of P59 in HG/HF-treated TCMK-1 cells, suggesting that P59 exerts its protective effects, at least in part, through PSS. These findings support C5aR2 agonism as a potential therapeutic strategy for DKD. Our study identified a C5aR2-PSS-MAM pathway that regulates metabolic homeostasis in TECs, providing mechanistic insight for C5aR2-targeted therapy. In contrast to broad complement inhibition, selective C5aR2 activation may protect the kidney while preserving essential host defense functions, an important consideration for chronic conditions such as DKD that require long-term treatment.

In conclusion, our study demonstrates that C5aR2 activation promotes c-FOS-mediated PSS transcription, thereby enhancing the PSS–MFN2 interaction and MAM formation. This pathway improves phospholipid metabolism and alleviates ER and mitochondrial dysfunction in TECs. These findings support C5aR2 agonism as a promising therapeutic strategy for DKD.

## Materials and methods

### Ethics

All medical research involving human participants was reviewed and approved by the Biomedical Research Ethics Committee of Peking University First Hospital (Ethics License No. 2025-036). All animal experiments were approved by the Ethics Committee of Peking University Health Science Center under license number J202199.

### Patients and tissue samples

Patients with type 2 diabetes mellitus (T2DM) and renal biopsy-confirmed DKD (*n* = 39) diagnosed at Peking University First Hospital from January 1, 2009, to December 31, 2019, were recruited for this study. T2DM and DKD were defined as previously described^46^. Patients with other known renal diseases were excluded. Clinical and pathological data were obtained from the electronic medical records of the patients at the hospital. The Chronic Kidney Disease Epidemiology Collaboration (CKD-EPI) equation was used to calculate the estimated glomerular filtration rate (eGFR)^47^. Renal biopsy samples were collected to detect C5aR2 protein expression in the kidney. Healthy control kidney samples (*n* = 9) were obtained from the non-tumorous poles of kidneys resected during tumor nephrectomy in individuals with no history of diabetes or other kidney diseases and were pathologically confirmed to be normal renal tissue. All renal biopsy samples were independently scored by two experienced pathologists who were blinded to the clinical and demographic data. Any discrepancies in scoring were rereviewed to reach a consensus.

The kidney endpoint was defined as a 40% reduction in the eGFR or ESKD. The definition of ESKD was consistent with that in previous studies^48^. Patients who did not reach the endpoint at the last visit were censored during the analysis. All medical research involving human participants was reviewed and approved by the Biomedical Research Ethics Committee of Peking University First Hospital (Ethics License No. 2025-036).

### Experimental animals

*C5ar2*^−/−^ mice were purchased from GemPharmatech Co., Ltd., and generated by Nanjing BioMedical Research Institute of Nanjing University (NBRI) via clustered regularly interspaced short palindromic repeats (CRISPR)-Cas9 genome engineering. To generate these mice, we transcribed sgRNA in vitro and microinjected Cas9 or sgRNA into the fertilized eggs of C57BL/6J mice. These correctly targeted mice verified by PCR and sequencing were mated with C57BL/6J mice to obtain a mouse model that could be stably inherited. The coding region of exon 2 of the *C5ar2-201* (ENSMUST00000098792.9) transcript, spanning approximately 1.1 kb in length, was selected as the knockout region, and genotyping was performed by PCR of tail DNA using gene-specific primers (Supplementary Fig. S1a, b). qRT-PCR analysis confirmed the absence of *C5ar2* expression in *C5ar2*^−/−^ mice (Supplementary Fig. S1c). For the experiments involving *C5ar2*^−/−^ mice, littermate WT mice with normal gene expression at 6–8 weeks of age were used. Male diabetic *db/db* (C57BL/6J-LepR^*db/db*^) mice and their nondiabetic *m/m* (C57BL/6J-LepR^+/+^) littermates were purchased from GemPharmatech Co., Ltd.

All the mice were maintained under specific pathogen-free conditions at the Laboratory Animal Center of Peking University First Hospital. All animal experiments were approved by the Ethics Committee of Peking University Health Science Center under license number J202199.

### Animal experiments

Four-week-old WT mice and *C5ar2*^−/−^ mice were fed a high-fat diet (HFD) (60% fat; Beijing Keao Xieli Feed Co., Ltd., China) for 4 weeks, followed by intraperitoneal injections of STZ (S0130; Sigma‒Aldrich) dissolved in 50 mM sodium citrate buffer (pH 4.5; Solarbio) at a dose of 60 mg/kg for 5 consecutive days. Afterward, the mice were fed an HFD for a total of 20 weeks. Mice fed a standard-fat diet (SFD, 10% fat) served as nondiabetic controls. One week after the STZ injections, fasting blood glucose levels were measured, and only mice with sustained hyperglycemia (fasting blood glucose concentration >16.7 mmol/L) were included in the diabetic group for subsequent studies.

In the PTEC-specific PSS2 overexpression study, DKD was induced in four groups of *C5ar2*^−/−^ and WT mice via the STZ/HFD protocol described above. One week after the STZ injections, one group of diabetic *C5ar2*^−/−^ mice and one group of diabetic WT mice received tail vein injections of AAV9 vectors carrying *Pss2* under the Ksp-cadherin promoter (AAV9-Ksp-*Pss2*). The other two groups were injected with control AAV9 vectors (AAV9-Ksp-Ctrl). Three weeks after virus infection, kidney tissues were collected to confirm PSS2 overexpression via western blotting and qRT-PCR.

For treatment studies in *db/db* mice, 8-week-old *db/db* mice were subcutaneously administered P59 (1 mg/kg, 3 mg/kg, or 5 mg/kg in PBS containing 0.05% (v/v) DMSO) or vehicle (PBS containing 0.05% (v/v) DMSO) every 24 h for 10 weeks. Age-matched *m/m* mice from the same litter treated with vehicle served as nondiabetic controls. For subsequent MAM fraction extraction, one group of *m/m* mice and one group of *db/db* mice were treated with 3 mg/kg P59, whereas the other two groups received vehicle subcutaneously every 24 h for 10 weeks.

### P59 synthesis

P59 was synthesized according to Croker et al. by SciLight Biotechnology, LLC (Beijing, China), with an acetylated N-terminus and a purity of > 98%^37^. The C-terminus of the peptide was left unmodified. Purity was confirmed by analytical high-performance liquid chromatography (HPLC) and liquid chromatography‒mass spectrometry (LC‒MS) analysis.

### Laboratory data assessments

The body weights of the mice were measured to an accuracy of 0.01 g. Fasting blood glucose, total cholesterol, and triglyceride levels were quantified via a GM3000 Chemistry Analyzer (Promega Biotech Co., Ltd.). The urinary albumin concentration was determined with a mouse ALB ELISA kit (E99-134; Bethyl Laboratories). Urinary creatinine levels were determined with a creatinine assay kit (DICT-500; BioAssay Systems). The uACR was calculated as the ratio of the urinary albumin concentration (μg) to the urinary creatinine concentration (mg).

### Cell culture and treatment

The TCMK-1 cell line was purchased from Fu Heng Biotechnology (China) and cultured in basic essential medium (PM150411, Procell). HK-2 human renal proximal tubule cells (ATCC, Rockville, MD) were cultured in DMEM/F-12 medium (CM-0109; Procell, Wuhan, China). HEK293T cells (ATCC) were cultured in complete cell medium (CM-0005; Procell, Wuhan, China). All cells were maintained at 37 °C in a humidified atmosphere containing 5% CO_2_.

PRTECs were isolated from 3- to 4-week-old male mice. Bilateral kidneys were harvested, and the renal cortex was minced thoroughly with surgical scissors, followed by centrifugation at 1500 rpm for 5 min. After the supernatant was discarded, type II collagenase solution (Worthington Chemical, USA) was added. The suspension was filtered through 70-μm and 40-μm cell strainers, and digestion was terminated by the addition of DMEM/F-12 (Gibco, USA) supplemented with 10% FBS (Gibco, USA). The cell suspension was centrifuged at 150 × *g* for 10 min, and the cell pellet was resuspended in fresh medium and cultured at 37 °C with 5% CO_2_.

On the day before transfection, the TCMK-1 cells were cultured to approximately 60% confluence. Targeted mouse *C5ar2*, *Pss1*, and *Pss2* siRNAs (RiboBio) were transfected into cells via Dona Transfection Reagent (DN001; D-Nano Therapeutics, China) according to the manufacturer’s instructions. A nontargeting siRNA (RiboBio) was used as the negative control in parallel cultures. At 24 h post-transfection, the cells were treated with HG/HF (final concentration of 30 mM glucose and 300 μM saturated free fatty acid palmitate [16:0]) for 48 h and then harvested^49^. A blank group (medium only) was used as the normal control. Mannitol and bovine serum albumin (M/B) were used as osmotic controls, with the M/B group maintained at a physiological glucose concentration of 5.5 mM and supplemented with 24.5 mM mannitol to ensure osmotic balance.

To investigate the effect of P59 on HG/HF-induced damage in TCMK-1 cells, the cells were treated with HG/HF and 100 μM P59 for 48 h. To overexpress PSS2, TCMK-1 cells were infected with lentivirus (Shanghai Genechem Co., Ltd.). Puromycin selection was applied to establish a stable cell line. The overexpression of PSS2 was confirmed by western blotting analysis (Supplementary Fig. S6k).

### Cell Counting Kit-8 (CCK-8) assay

TCMK-1 cells were seeded in 96-well plates at a density of 1.5 × 10³ cells/mL and subsequently treated as described above. Cell culture medium (100 μL per well) and CCK-8 reagent (CK001; LABLEAD Trading Co., Ltd., Beijing, China) were added, and the mixture was incubated at 37 °C for 2 h. Absorbance was measured at 450 nm via a microplate reader (GM3000; Promega Biotech Co., Ltd.). Each condition was performed in triplicate. The formula for determining cell viability was as follows: cell viability (%) = (experimental group/control group) × 100%. The experiment was independently repeated three times.

### Histological analysis

In the kidney tissues of patients, the scoring of glomerular and tubulointerstitial lesions was consistent with the definitions used in a previous study^50^.

In mouse kidney tissues, twenty glomeruli from each renal section were analyzed at 200× magnification. The glomerular area was measured by tracing the periphery of the glomerular tuft. Mesangial matrix expansion was defined as the area with positive PAS staining devoid of nuclei and is expressed as a ratio of the total glomerular area. Positive staining signals were quantified via Image-Pro Plus V6.0 (Media Cybernetics, Bethesda, MD). The tubulointerstitial injury index was assessed by evaluating tubular dilatation, atrophy, and tubular cell loss. The results of the quantitative assessment of interstitial damage are consistent with the definitions used in a previous study^51^.

### TEM and MAM analysis

Briefly, renal cortical tissues from mice were sliced into three sections and immediately immersed in precooled 3% glutaraldehyde at 4 °C for fixation. Further processing was performed at the Electron Microscopy Laboratory of Peking University First Hospital. Ultrathin sections were randomly obtained from three tissue slices from each mouse, and 15 representative nonoverlapping electron micrographs of the glomeruli were captured via a Hitachi 7700 transmission electron microscope at a magnification of ×12,000. The GBM thickness and foot process width were measured via Image-Pro Plus software V6.0^52–54^.

For MAM analysis, 15 additional high-magnification (20,000×) TEM images per mouse were acquired from renal cortical regions to assess mitochondria‒ER interactions. Mitochondria in clear proximity to the ER were identified and analyzed using Amira software (Thermo Fisher Scientific). The length of the ER membrane running parallel to the mitochondrial outer membrane at a distance of less than 30 nm was measured and defined as the MAM length. The MAM contact ratio was calculated by normalizing the MAM length to the mitochondrial perimeter. All analyses were performed in a blinded manner, with at least 50 mitochondria evaluated per group.

### IHC and IF

For immunohistochemical staining, paraffin-embedded kidney sections were stained with antibodies against C5aR2 (1:100; sc-515734; Santa Cruz Biotechnology), α-SMA (1:1000; ab32575; Abcam), F4/80 (1:100; MCA497; Bio-Rad), ADRP (1:200; ab52356; Abcam), TGF-β (1:200; ab170874; Abcam), PSS1 (1:200; ab157222; Abcam), and PSS2 (1:200; ARP49961_P050; Aviva Systems Biology Corporation). Ten nonoverlapping fields per kidney section were captured at 200× magnification for systematic quantification. Protein expression levels were determined by measuring either the integrated optical density (IOD) or the mean optical density (MOD = IOD/area) using Image-Pro Plus 6.0 software. For immunofluorescence colocalization studies, kidney sections were simultaneously probed with: (1) anti-C5aR2 (1:100; sc-515734; Santa Cruz Biotechnology), (2) the proximal tubule marker AQP-1 (1:200; ab168387; Abcam; a marker for PTECs), and (3) the distal tubule marker calbindin-D28K (1:100; 14479-1-AP; Proteintech; a marker for distal renal tubular epithelial cells). The samples were subsequently incubated with the species-matched fluorescent secondary antibodies Alexa Fluor 488-conjugated goat anti-mouse IgG (1:200; A32723; Invitrogen) and Alexa Fluor 594-conjugated donkey anti-rabbit IgG (1:200; A21207; Invitrogen). The nuclei were counterstained with 4’,6-diamidino-2-phenylindole (DAPI) before imaging.

### Live-cell confocal microscopy and analysis

To visualize and quantify MAM formation in live cells, we performed high-resolution confocal microscopy followed by three-dimensional analysis. In brief, TCMK-1 cells were transfected with a sec61β-GFP plasmid to fluorescently label the ER. At 24 h post-transfection, the mitochondria were stained with Mito Orange (PKMO-1; Genvivo Biotechnology Co., Ltd., Nanjing, China) for 15 min. Live-cell imaging was immediately carried out using a Leica STELLARIS confocal microscope (Leica Microsystems, Germany) equipped with an environmental chamber maintained at 37 °C and 5% CO₂.

Z-stack images were acquired at 0.35-μm intervals throughout the entire cell volume. Three-dimensional visualization, colocalization analysis, and orthogonal projections were performed using Imaris 10.2 software (Oxford Instruments). The extent of ER–mitochondria colocalization was quantitatively assessed by calculating Pearson’s correlation coefficient between the green (ER) and red (mitochondria) channels to enable direct morphological assessment of ER–mitochondria contact sites.

### Western blotting assay

Total protein was extracted from cultured TCMK-1 cells or mouse kidney tissues via radioimmunoprecipitation assay (RIPA) buffer (32010A; Bestbio, Shanghai, Co., Ltd.) or by isolation of organelles. The samples were separated on a 10% SDS-PAGE gel and transferred to PVDF membranes. The membranes were blocked with 5% skim milk and then incubated with primary antibodies against C5aR2 (sc-515734; Santa Cruz Biotechnology), PSS1 (ab157222; Abcam), PSS2 (ARP49961_P050; Aviva Systems Biology Corporation), MFN2 (12186-1-AP; Proteintech), XBP-1s (143F; BioLegend), p-EIF2α (28740-1-AP; Proteintech), EIF2α (11170-1-AP; Proteintech), CHOP (15204-1-AP; Proteintech), COX IV (11242-1-AP; Proteintech), calnexin (10427-2-AP; Proteintech), ERK1/2 (4695; Cell Signaling Technology), p-ERK1/2 (4370; Cell Signaling Technology), HA (ab9110, Abcam), FLAG (ab213519, Abcam), α-tubulin (HRP-80762; Proteintech), and β-actin (Ac028; ABclonal). The membranes were subsequently incubated with HRP-conjugated secondary antibodies (SA00001; Proteintech). Proteins were visualized via an ECL Chemiluminescent Substrate Kit (WBKLS0500, Millipore) and quantified via ImageJ software with the Fiji plugin (NIH, Bethesda, MD, USA).

### qRT-PCR analysis

Total RNA was extracted from cells or mouse kidney tissues via the SteadyPure Quick RNA Extraction Kit (AG21023; Accurate Biotechnology, Hunan, Co., Ltd.). Two micrograms of RNA were reverse transcribed into cDNA via the PrimeScript RT Reagent Kit (RR037A; Takara Biomedical Technology, Beijing, Co., Ltd.). qRT-PCR analysis was performed using SYBR Green Master Mix (A25742; Applied Biosystems; Thermo Fisher Scientific) with 2 μL of cDNA and forward and reverse primers. Relative gene expression was calculated after normalization to 18S rRNA and compared to that of control samples. The primers used for the qRT-PCR assays are listed in Supplementary Table S7.

### Protein docking analysis

Protein docking analysis was performed using the ZDOCK 3.0 program. All docking analyses were based on experimentally determined structural fragments retrieved from the Protein Data Bank (PDB) database: MFN2 (PDB ID: 6JFK, GTPase domain fragment), PSS1 (PDB ID: 9B4F, catalytic core region fragment), and PSS2 (PDB ID: 9N0X, catalytic core region fragment).

### Co-IP

Co-IP was performed using the Pierce Crosslinking Magnetic Co-IP Kit (88805; Thermo Fisher Scientific). For endogenous protein interaction analysis, TCMK-1 cells were lysed, and 500 μg of protein was incubated with anti-FLAG, anti-MFN2, or anti-IgG antibodies on magnetic beads overnight at 4 °C. For MFN2 truncation mutant binding assays, HEK293T cells were co-transfected with FLAG-tagged MFN2 (full-length or truncation mutants) and HA-tagged PSS1 or PSS2 plasmids. At 48 h post-transfection, the cells were lysed and immunoprecipitated using anti-FLAG or anti-HA antibodies. The beads were subsequently washed, eluted with low-pH buffer, and heated at 95 °C with 5× SDS-PAGE sample loading buffer. Protein samples were separated via 10% SDS-PAGE and analyzed by Western blotting assay.

### ChIP assay

The ChIP assay was performed via the SimpleChIP Enzymatic Chromatin IP Kit (9003S; Cell Signaling Technology). Briefly, TCMK-1 cells were harvested via a cell scraper after 20 min of incubation in cold lysis buffer. DNA was immunoprecipitated from sonicated cell lysates via an anti-c-FOS antibody (2250; Cell Signaling Technology). The promoter-specific primers for mouse *Pss2* used in subsequent PCR amplification are listed in Supplementary Table S7.

### Transcription factor prediction and dual-luciferase reporter assays

The prediction of c-FOS as a transcription factor of *Pss1* and *Pss2* was completed at the University of California, Santa Cruz (UCSC; https://genome.ucsc.edu/) Genome Browser Home. Mouse *Pss1* and *Pss2* promoters (spanning −2000 to +100 bp) or their variants were subsequently synthesized, cloned, and inserted into the GV238 vector (Shanghai Genechem Co., Ltd.). To detect the promoter activity of the *Pss1* and *Pss2* plasmids, the reporter plasmid and CV702-*c-Fos* plasmid were transfected into TCMK-1 cells in 12-well plates for 24 h via Lipofectamine 3000. Dual-luciferase reporter assays were performed via the Luc-Pair Duo-Luciferase HS Assay Kit (Promega Biotech Co., Ltd.) according to the manufacturer’s instructions. Firefly luciferase activity was normalized to Renilla luciferase activity for each sample.

### Analysis of lipid deposition

For Nile Red staining, the cells were processed as described above. The cells in the 24-well plates were fixed with 4% paraformaldehyde for 10 min, rinsed twice with PBS, and stained with Nile Red (0.1 μg/mL; HY-D0718; MedChemExpress) for 30 min. The coverslips were sealed with anti-fade mounting medium containing DAPI, and the samples were observed and photographed via a fluorescence inverted microscope. Image-Pro Plus software V.6.0 was used to evaluate the positive staining of lipids. Positive signals were normalized to the total number of nuclei (DAPI) counted per well. The Nile Red staining conditions for frozen mouse kidney samples were identical to those described above for cell staining. Positive signals were normalized to the total tissue area of each image.

The Oil Red O staining procedure followed the instructions of the Oil Red O Staining Kit (G1261; Beyotime Biotechnology). The cells were stained with Oil Red O working solution for 20 min, after which the nuclei were counterstained with hematoxylin staining solution. The samples were then observed and photographed under a microscope. Image-Pro Plus software V.6.0 was used to evaluate the positive staining of lipids. Positive signals were normalized to the total number of nuclei (stained with hematoxylin) counted per well. The Oil Red O staining conditions for frozen mouse kidney samples were identical to those described above for cell staining. Positive signals were normalized to the total tissue area of each image.

### Cell fraction isolation

Subcellular isolation was performed on freshly isolated mouse kidney tissues as previously described^55^. Briefly, the tissues were finely minced and homogenized in Buffer 1 (225 mM mannitol, 75 mM sucrose, 0.5% BSA, 0.5 mM EGTA, and 30 mM Tris-HCl, pH 7.4) via a Dounce homogenizer. The homogenate was subsequently centrifuged at 740× *g* for 5 min at 4 °C. The supernatant was further centrifuged at 9000× *g* for 10 min to obtain a crude mitochondrial pellet from the ER-enriched supernatant. The supernatant was reserved for ER isolation, while the crude mitochondrial pellet was resuspended in Buffer 2 (225 mM mannitol, 75 mM sucrose, 0.5% BSA, and 30 mM Tris-HCl; pH 7.4) and centrifuged at 10,000× *g* for 10 min at 4 °C.

The resulting pellet was resuspended in Buffer 3 (225 mM mannitol, 75 mM sucrose, and 30 mM Tris-HCl, pH 7.4) and centrifuged again at 10,000× *g* for 10 min at 4 °C. The crude mitochondrial pellet was resuspended in mitochondrial resuspension buffer (MRB; 250 mM mannitol, 5 mM HEPES, pH 7.4, and 0.5 mM EGTA).

For MAM isolation, the crude mitochondrial fraction was subjected to ultracentrifugation at 95,000× *g* for 30 min at 4 °C in Percoll medium (225 mM mannitol, 25 mM HEPES, pH 7.4, 1 mM EGTA, and 30% (vol/vol) Percoll). The MAM layer and mitochondrial pellet were carefully collected and resuspended in MRB.

The MAM and mitochondrial suspensions were centrifuged at 6300× *g* for 10 min at 4 °C. The supernatant was then centrifuged at 20,000× *g* for 30 min at 4 °C, followed by ultracentrifugation at 100,000× *g* for 1 h at 4 °C. The isolated subcellular fractions were resuspended in lysis buffer supplemented with protease and phosphatase inhibitors. Proteins were resolved by 10% SDS-PAGE and analyzed by immunoblotting.

### In situ PLA

The protein‒protein interactions were analyzed using Duolink PLA (Sigma-Aldrich; DUO92002/4) according to standard protocols. In brief, cells were seeded on 6-mm coverslips in 48-well plates, washed with PBS, and fixed with 4% paraformaldehyde. Following blocking, primary antibody incubation was carried out at 4 °C overnight. After washing, species-matched PLA probes were applied. Subsequent ligation and amplification reactions were conducted according to the manufacturer’s guidelines, with final mounting in Duolink medium with DAPI nuclear stain (DUO82040; Sigma-Aldrich). Fluorescence signals were captured using an inverted fluorescence microscope system.

The primary antibodies used included mouse anti-VDAC1 (1:100; 66345-1-Ig; Proteintech), rabbit monoclonal anti-IP3R1 (1:100; 19962-1-AP; Proteintech), mouse anti-FLAG (1:100; 66008-4-Ig; Proteintech), and rabbit anti-MFN2 (1:100; 12186-1-AP; Proteintech). The fluorescence signals were quantified via Image-Pro Plus software V.6.0.

### Quantification of PS

To quantify the PS levels in the isolated MAM fraction, we used a PS assay kit (ab273295; Abcam). This assay is based on an enzymatic reaction in which PS is hydrolyzed to produce phosphatidic acid and L-serine. The released L-serine reacts with a specific probe to generate a stable fluorophore. The assay was performed according to the manufacturer’s instructions.

### Mitochondrial OCR measurement

The mitochondrial OCR was measured via a Seahorse XF96 analyzer (Agilent Technologies). After the cells were seeded in XF96 microplates and cultured overnight, experimental treatments were applied. Before the OCR was measured, the cells were washed with PBS and equilibrated for 1 h in prewarmed (37 °C) XF assay medium (102353-100; Agilent Technologies) supplemented with 1× GlutaMAX (Gibco, 35050), 1 mM sodium pyruvate (Sigma‒Aldrich, S8636), and 25 mM glucose (Sigma‒Aldrich, G7528), with the pH adjusted to 7.4. The sensor cartridge, prehydrated in XF calibrant, was loaded with mitochondrial modulators at the following specified concentrations: 1 μM oligomycin (103595-100; Agilent Technologies), 1 μM FCCP (103595-100; Agilent Technologies), 0.5 μM rotenone (103595-100; Agilent Technologies), and 0.5 μM antimycin A (103595-100; Agilent Technologies). Post-assay normalization was performed using DAPI nuclear staining to correlate the OCR with the cell count. Data acquisition and analysis were conducted using Seahorse Wave software.

### RNA-seq analysis

Total RNA was extracted from mouse renal cortex tissue via the SteadyPure Quick RNA Extraction Kit (AG21023; Accurate Biotechnology, Hunan, China). RNA integrity was assessed via a 2100 Bioanalyzer (Agilent Technologies), and only samples with an RNA integrity number (RIN) ≥ 7 were selected for downstream analysis. Transcriptome sequencing and subsequent bioinformatic analysis were performed by OE Biotechnology, Inc. Libraries were constructed via the TruSeq Stranded mRNA LT Sample Prep Kit (Illumina) following the manufacturer’s protocol and sequenced on an Illumina HiSeq 2500 platform, generating paired-end reads of 125 bp or 150 bp.

The raw count matrix was normalized via DESeq2 (version 1.32.0) to identify DEGs. Significantly DEGs were defined as those with *P* < 0.05 and an absolute log2-transformed fold change > 1. GSEA was performed to identify significant pathways, with the normalized enrichment score (NES) and false discovery rate (FDR) used as key metrics.

### Lipidomic analysis

Total lipids were extracted from mouse renal cortex tissue via a previously modified method^5^. The samples were immediately sealed, flash-frozen, and stored at −80 °C until lipid extraction. Lipid profiling was performed by OE Biotech Co., Ltd., via liquid chromatography‒tandem mass spectrometry (LC‒MS/MS). Data acquisition was conducted on a Q Exactive Orbitrap mass spectrometer coupled with an UltiMate 3000 ultra-performance liquid chromatography system (Thermo Fisher Scientific). Lipid identification and quantification were performed via LipidSearch software (version 4.1.16; Thermo Fisher Scientific).

KEGG enrichment analysis was performed on the basis of the terms in the KEGG database, with significant results filtered according to the following criteria: *P* < 0.05 and an absolute log2-transformed fold change > 1. LION analysis was performed via the LINT-web platform (Fudan University, Shanghai, China). LION analysis was used to compare significantly altered lipids to the total lipid set to identify enriched LION terms, with significance thresholds set at *P* < 0.05 and an absolute log2-transformed fold change >1. The lipidome dataset was subjected to heatmap analysis for visualization of lipid species, and box-whisker plots were generated to summarize lipid class distributions.

### scRNA-seq analysis

Unique molecular identifier (UMI) count matrices were processed with the Seurat R package (version 4.0.0)^56^. Following the preliminary filtering by Cell Ranger, additional stringent quality control was applied to exclude potential low-quality or artifactual cells. Specifically, cells were removed if they met any of the following criteria: (1) < 114 detected genes, (2) < 500 UMIs, (3) mitochondrial UMI counts exceeding four times the median mitochondrial content across all cells, or (4) hemoglobin gene proportion > 5%. The DoubletFinder package (version 2.0.3) was employed to identify singlets for downstream analyses^57^. The gene expression values were subsequently normalized via the normalizeData function.

The top 2000 highly variable genes were calculated via the Seurat function FindVariableGenes, and data scaling was performed with ScaleData. Principal component analysis (PCA) was conducted to reduce dimensionality via the RunPCA function. The true dimensionality of the data was estimated via ElbowPlot, DimHeatmap, and JackStrawPlot. Cell clustering was then performed via FindNeighbors and FindClusters, followed by visualization via UMAP with the RunUMAP function. The full Seurat workflow used in this study is consistent with the official tutorial (https://satijalab.org/seurat/articles/pbmc3k_tutorial.html). The sequencing and bioinformatic analyses were performed by OE Biotech Co., Ltd. (Shanghai, China).

### Cell type annotation and cluster marker identification

Following nonlinear dimensionality reduction via UMAP, the cells were projected into a two-dimensional space and grouped on the basis of shared transcriptional profiles. To identify characteristic genes for each cluster, the FindAllMarkers function from Seurat was employed. Cluster annotation was then performed by evaluating the expression patterns of established canonical markers corresponding to known cell types. For PTEC subpopulation classification, we implemented a refined stratification system in which healthy S1/2 segments were defined by elevated expression of segment-specific markers, including solute carrier family 5 member 12 (*Slc5a12*) and solute carrier family 34 member 1 (*Slc34a1*), combined with minimal expression of injury-associated genes. Injured S1/2 segments maintained their segmental identity markers (*Slc5a12* and *Slc34a1*), with moderately increased injury-related gene expression. Injured S3 segments demonstrated characteristic solute carrier family 22 member 6 (*Slc22a6*) expression with elevated injury marker levels. The failed-repair PT cell population was characterized by diminished segment-specific transporter expression, accompanied by persistently high expression of injury response genes, including doublecortin domain-containing 2A (*Dcdc2a*) and proliferation markers such as the proliferation marker Ki-67 (*Mki67*)^58,59^. To assess gene expression patterns across clusters, average expression levels were calculated via the average expression function. DEGs were screened via the FindMarkers function (test.use = wilcox). A *P* value < 0.05 and a |log2fold change| > 0.58 were set as the thresholds for significant differential expression. GO enrichment analysis of the DEGs was performed via R software (version 4.0.3) on the basis of the hypergeometric distribution.

### Reagents, antibodies, and primers

Detailed information regarding all antibodies and primers used in this study is provided in Supplementary Tables S7 and S8.

### Statistical analysis

The statistical methods, tools, and significance thresholds for each analysis are specified in the corresponding figure legends. Normally distributed continuous variables are expressed as the mean ± standard deviation (SD), and nonnormally distributed continuous variables are presented as the median (interquartile range, IQR). Categorical variables are presented as absolute counts and percentages. Statistical comparisons included chi-square tests for categorical data, unpaired Student’s *t*-tests for normally distributed continuous variables in two-group comparisons, and Mann‒Whitney U tests for nonnormally distributed continuous variables. Multiple group comparisons were analyzed via one-way analysis of variance (ANOVA) with Tukey’s post hoc tests. Statistical significance was defined as *P* < 0.05 (*), *P* < 0.01 (**), *P* < 0.001 (***), and *P* < 0.0001 (****). All analyses were performed via SPSS (version 26.0; IBM Corp., Armonk, NY), R (version 4.4.3; R Development Core Team, Vienna, Austria), and GraphPad Prism (version 10.0; GraphPad Software, San Diego, CA).

## Supplementary information

Supplementary Information

## Data Availability

All the data and code generated in this study are available upon reasonable request by contacting the corresponding author, M.C.
