# Supplementary material for: Complement 5a receptor 2 attenuates diabetic kidney disease by promoting mitochondria-associated endoplasmic reticulum membrane formation mediated by PSS-MFN2 interaction
Source: Cell Discov. 2026 Mar 31;12:24. doi: 10.1038/s41421-026-00873-w (PMC13040050; doi:10.1038/s41421-026-00873-w)
Supplement: Supplementary file 1 — Supplementary Information [file 41421_2026_873_MOESM1_ESM.pdf]

Supplementary Information

Complement 5a receptor 2 attenuates diabetic kidney disease by promoting mitochondria-associated endoplasmic reticulum membrane formation mediated by PSS-MFN2 interaction

Authors: Yiyang Zhao[1,2], Yihui Wang[1,2,3], Zihan Li[1,2], Dongyuan Chang[1,2], Lin Nie[1,4], Minghui Zhao[1,2,3], Sydney Chi Wai Tang [5], *Min Chen[1,2,6]

Affiliations:

[1] Renal Division, Department of Medicine, Peking University First Hospital; Peking University Institute of Nephrology; Key Laboratory of Renal Disease, Ministry of Health of China; Key Laboratory of Chronic Kidney Disease Prevention and Treatment (Peking University), Ministry of Education, Beijing, China.

[2] Research Units of Diagnosis and Treatment of Immune-mediated Kidney Diseases, Chinese Academy of Medical Sciences, Beijing, China.

[3] Peking-Tsinghua Center for Life Sciences, Beijing, China.

[4] Laboratory of Electron Microscopy, Pathological Center, Peking University First Hospital, Beijing, China.

[5] Division of Nephrology, Department of Medicine, School of Clinical Medicine, The University of Hong Kong, Queen Mary Hospital, Hong Kong, China.

[6] State Key Laboratory of Vascular Homeostasis and Remodelling, Peking University, Beijing, China.

Running title: C5aR2 activation alleviates DKD

First author

Yiyang Zhao

Telephone: +86-15797896480 E-mail: ashelyzhaoyiyang@163.com

***Corresponding author**

Min Chen

Renal Division, Department of Medicine,
Peking University First Hospital; Beijing 100034, China
Fax: +86-10-66551055, Telephone: +86-10-66551736
E-mail: chenmin74@sina.com

Supplementary Table S1. Baseline clinical characteristics of the patients.

Variables	Overall (*N* = 39)	Low C5aR2 (*N* = 18)	High C5aR2 (*N* = 21)	*P*. Value
**Age (years)**	48.2 ± 1.8	52.1 ± 2.7	45.8 ± 2.4	0.086
**Male Sex (%)**	11 (28.2)	6 (33.3)	5 (23.8)	0.626
**BMI (kg/m^2)**	26.1 ± 5.3	25.9 ± 0.9	26.1 ± 0.8	0.844
**DR (%)**	28 (71.8)	13 (72.2)	15 (71.4)	0.967
**eGFR (mL/min/1.73 m^2)**	49.4 ± 5.2	57.5 ± 8.2	42.5 ± 6.4	0.155
**Scr (μmol/L)**	216.2 ± 30.5	161.8 ± 35.4	260.1 ± 45.6	0.169
**Proteinuria (g/24 hr)**	4.8 ± 0.6	3.4 ± 0.6	6.0 ± 0.8	**0.015**
**Serum albumin (g/L)**	33.7 (28.9, 38.0)	36.1 (31.0, 38.6)	31.8 (27.2, 34.4)	**0.040**
**Hb (g/L)**	116.5 ± 3.7	123.8 ± 6.0	110.7 ± 4.2	0.164
**HbA1c (%)**	7.4 (5.9, 8.3)	7.8 (6.3, 8.5)	6.8 (5.7, 8.2)	0.465
**LDL-cholesterol (mmol/L)**	2.5 (1.95, 3.87)	2.8 (1.7, 4.0)	2.2 (2.0, 3.8)	0.975
**HDL-cholesterol (mmol/L)**	1.0 (0.87, 1.35)	1.1 (0.9, 1.3)	1.0 (0.8, 1.4)	0.856
**TG (mmol/L)**	1.7 (1.2, 2.7)	1.5 (1.2, 2.6)	1.7 (1.3, 3.8)	0.541
**K-W nodules (grades 0-1) (%)**	30 (76.9)	13 (72.2)	17 (81.0)	0.646
**RPS class (class I-IV) (%) I/II/III/IV**	2 (5.1)/7 (17.9)/ 29 (74.4)/1 (2.6)	1 (5.6)/4 (22.2)/ 13 (72.2)/0 (0.0)	1 (4.8)/3(14.3)/ 16 (76.2)/1 (4.8)	0.646
**Interstitial inflammation (grades 0-2) (%) 0/1/2**	0 (0.0)/14 (35.9)/ 25 (64.1)	0 (0.0)/9 (50.0)/ 9 (50.0)	0 (0.0)/5 (23.8)/ 16 (76.2)	0.165
**IFTA (grades 0-3) (%)0/1/2/3**	0 (0.0)/14(35.9)/ 11 (28.2)/14 (35.9)	0 (0.0)/10 (55.6)/ 6 (33.3)/2 (11.1)	0 (0.0)/4 (19.0)/ 5 (23.8)/12 (57.1)	**0.004**
**Arteriosclerosis (grades 0-2) (%) 0/1/2**	1 (2.6)/24 (61.5)/ 14 (35.9)	1 (5.6)/12 (66.7)/ 5 (27.8)	0 (0.0)/12 (57.1)/ 9 (42.9)	0.335
**Arteriolar hyalinosis (grades 0-2) (%) 0/1/2**	0 (0.0)/8 (20.5)/ 31 (79.5)	0 (0.0)/5 (27.8)/ 13 (72.2)	0 (0.0)/3 (14.3)/ 18 (85.7)	0.477
**Follow-up time (years)**	40.0 (12.0, 48.0)	44.5 (23.0, 51.3)	24 (4.0, 41.5)	**0.007**
**Kidney endpoint (%)**	21 (53.8)	4 (22.2)	17 (81.0)	**0.001**

The data are presented as the means ± SDs, medians (IQRs), and proportions. The Mann–Whitney U test and the unpaired Student's t test were used to compare nonnormally and normally distributed continuous data, respectively. Categorical data were assessed by the chi-square test.

BMI, body mass index; DBP, diastolic blood pressure; DR, diabetic retinopathy; eGFR, estimated glomerular filtration rate; HbA1c, hemoglobin A1C; Hb, hemoglobin; HDL-cholesterol, high-density lipoprotein-cholesterol; LDL-cholesterol, low-density lipoprotein-cholesterol; SBP, systolic blood pressure; Scr, serum creatinine; TG, triglyceride.

Supplementary Table S2. Laboratory data of *C5ar2*$^{-/-}$ mice.

Variables	WT mice		*C5ar2*$^{-/-}$ mice	
	SFD	STZ/HFD	SFD	STZ/HFD
**BW (g)**	28.81 ± 1.23	21.6 ± 1.38****	32.14 ± 1.05****	26.60 ± 1.15$^{####}$
**FBG (mmol/l)**	8.20 ± 0.85	23.15 ± 3.60****	8.08 ± 0.76	21.90 ± 3.14
**uACR (μg/mg)**	20.56 ± 3.56	92.68 ± 16.32***	18.90 ± 5.27	160.10 ± 63.92$^{##}$
**TG (mmol/l)**	1.03 ± 0.24	1.81 ± 0.25**	1.01 ± 0.27	2.57 ± 0.72$^{##}$
**TCHO (mmol/l)**	4.70 ± 0.54	8.51 ± 1.30****	3.81 ± 0.44	8.53 ± 1.76

BW, body weight; FBG, fasting blood glucose; uACR, urine albumin-to-creatinine ratio; TG, triglyceride; TCHO, total cholesterol. The continuous data are expressed as the means ± SDs. ** $P < 0.01$, *** $P < 0.001$, **** $P < 0.0001$ vs. WT-SFD; $^{##}$ $P < 0.01$; $^{####}$ $P < 0.0001$ vs. WT-STZ/HFD. The data were analysed by two-sided one-way ANOVA with Tukey's test.

Supplementary Table S3. Lipidomic profiling of individual lipid species in the renal cortex of diabetic *C5ar2*$^{-/-}$ mice and diabetic WT mice.

subclass	STZ/HFD							
	C5ar2$^{-/-}$ mice-1	*C5ar2*$^{-/-}$ mice-2	*C5ar2*$^{-/-}$ mice-3	*C5ar2*$^{-/-}$ mice-4	WT mice-1	WT mice-2	WT mice-3	WT mice-4
CL (18:1)	0.192	0.125	0.012	0.191	0.302	0.174	0.299	0.185
CL (18:2)	0.384	0.249	0.024	0.381	0.603	0.347	0.599	0.369
CL (22:6)	0.192	0.125	0.012	0.191	0.302	0.174	0.299	0.185
ChE (20:4)	0.000	0.022	0.045	0.008	0.005	0.068	0.179	0.326
DG (16:0)	5.590	5.186	4.130	6.039	3.226	3.197	2.896	4.087
DG (18:0)	1.587	1.973	1.610	2.001	1.179	1.214	1.156	1.738
DG (18:1)	4.673	3.825	2.897	5.922	2.374	2.390	2.043	2.817
DG (18:2)	6.543	4.060	3.291	7.743	2.592	3.097	2.206	2.857
DG (20:1)	0.067	0.061	0.048	0.088	0.041	0.044	0.039	0.056
DG (20:3)	0.435	0.418	0.295	0.531	0.205	0.283	0.195	0.361
DG (20:4)	2.490	2.618	1.920	2.604	1.312	1.827	1.592	1.802
DG (22:5)	0.169	0.330	0.188	0.313	0.134	0.173	0.147	0.259
DG (22:6)	1.951	2.211	2.386	2.459	1.729	1.880	1.886	2.669
DG (36:3)	0.486	0.457	0.167	0.323	0.013	0.135	0.028	0.148
FA (20:4)	22.337	39.336	93.482	38.781	36.672	67.017	56.468	38.031
FA (20:5)	4.004	4.313	16.798	6.050	3.993	7.756	6.102	4.454
LPA (16:0)	1.017	0.602	2.086	0.715	1.133	0.852	0.671	0.663
LPA (18:0)	1.363	0.501	1.081	0.475	1.203	1.188	0.551	0.526
LPA (20:4)	0.179	0.067	0.616	0.146	0.201	0.290	0.186	0.087
LPA (22:6)	0.055	0.051	0.215	0.045	0.095	0.083	0.063	0.043
LPC (14:0)	0.724	0.411	0.331	0.382	0.282	0.466	0.165	0.305
LPC (16:0)	100.735	93.661	85.890	84.294	71.718	92.877	73.830	85.698
LPC (16:1)	0.855	0.503	0.648	0.675	0.537	0.787	0.621	0.561
LPC (18:0)	73.026	69.155	70.289	65.551	53.624	65.675	55.882	71.971
LPC (18:1)	10.373	8.904	9.417	9.181	8.314	6.222	8.087	9.135
LPC (18:2)	12.033	8.281	10.391	9.700	8.159	7.754	5.503	6.373
LPC (18:3)	7.768	7.654	6.838	7.487	6.405	7.127	6.722	7.772
LPC (20:0)	0.635	0.642	0.638	0.578	0.596	0.863	0.647	0.730
LPC (20:1)	0.311	0.262	0.229	0.259	0.242	0.392	0.267	0.296

LPC (20:3)	0.698	0.387	0.515	0.613	0.428	0.563	0.323	0.487
LPC (20:4)	1.813	1.040	1.279	1.356	1.631	1.466	1.604	1.429
LPC (20:5)	0.375	0.091	0.250	0.298	0.044	0.053	0.040	0.208
LPC (22:0)	0.375	0.334	0.334	0.250	0.274	0.377	0.298	0.395
LPC (22:3)	0.040	0.064	0.052	0.070	0.039	0.063	0.043	0.068
LPC (22:4)	0.198	0.186	0.140	0.149	0.151	0.222	0.098	0.161
LPC (22:6)	1.926	1.120	1.412	1.299	1.810	1.269	1.547	1.541
LPC (24:0)	0.486	0.556	0.496	0.409	0.465	0.597	0.530	0.581
LPC (24:1)	0.040	0.110	0.064	0.066	0.065	0.076	0.078	0.091
LPC (26:3)	0.021	0.036	0.014	0.020	0.020	0.037	0.024	0.038
LPC (28:0)	0.442	0.408	0.187	0.355	0.056	0.212	0.082	0.124
LPC (32:0)	2.968	2.568	2.160	2.135	2.030	2.280	1.879	1.814
LPC (34:1)	2.438	1.983	1.584	1.970	1.229	1.631	1.191	1.223
LPC (34:3)	3.317	2.211	2.761	2.863	2.100	2.149	1.879	2.132
LPC (34:4)	0.125	0.229	0.107	0.082	0.082	0.129	0.076	0.114
LPC (36:4)	2.127	2.743	1.822	1.636	1.981	2.254	2.203	2.406
LPC (36:5)	13.412	15.693	19.338	14.133	24.913	25.337	31.342	26.119
LPC (36:6)	3.055	3.133	2.656	2.036	1.791	2.004	1.227	2.139
LPC (38:4)	0.463	0.727	0.525	0.379	0.417	0.668	0.445	0.389
LPC (38:5)	2.060	2.066	1.593	1.750	1.728	3.101	1.806	1.639
LPC (38:6)	9.892	9.428	10.241	9.562	11.246	9.627	10.771	10.204
LPE (14:0)	0.212	0.139	0.558	0.109	0.054	0.092	0.064	0.063
LPE (16:0)	51.957	50.207	133.532	41.546	44.682	79.744	47.402	39.124
LPE (16:1)	0.317	0.189	1.422	0.289	0.259	0.444	0.346	0.223
LPE (18:0)	43.941	33.320	92.058	37.769	39.676	64.028	36.077	32.566
LPE (18:1)	9.358	10.618	16.988	7.014	8.611	16.046	9.490	6.843
LPE (18:2)	6.176	4.763	24.581	6.401	7.072	9.061	6.733	4.118
LPE (18:3)	0.084	0.033	0.288	0.094	0.061	0.084	0.055	0.035
LPE (20:0)	0.152	0.091	0.407	0.117	0.152	0.342	0.307	0.182
LPE (20:1)	0.069	0.133	0.201	0.107	0.126	0.177	0.199	0.115
LPE (20:4)	35.275	25.584	177.473	43.434	63.080	70.950	85.172	39.529
LPE (20:5)	0.362	0.224	1.652	0.436	0.464	0.588	0.600	0.304
LPE (22:4)	1.453	1.517	5.362	1.356	1.890	3.131	2.447	1.669
LPE (22:5)	0.639	0.652	2.895	0.978	0.779	1.196	1.195	0.553

LPE (22:6)	36.903	31.601	214.486	44.244	63.456	58.364	69.173	36.309
LPE (34:2)	1.138	1.416	0.881	1.037	1.076	1.235	1.304	1.074
LPG (16:0)	1.668	1.072	6.501	2.651	3.129	3.901	2.384	1.655
LPG (16:1)	0.164	0.070	0.767	0.187	0.156	0.299	0.145	0.076
LPG (18:0)	0.120	0.125	0.446	0.198	0.142	0.223	0.116	0.143
LPG (18:1)	4.272	4.399	19.598	5.065	3.943	9.486	3.984	3.002
LPG (18:2)	5.126	4.379	24.011	6.441	3.742	9.958	3.921	3.204
LPG (18:3)	0.236	0.088	1.152	0.285	0.122	0.420	0.095	0.063
LPG (20:2)	0.119	0.089	0.606	0.107	0.128	0.179	0.091	0.081
LPG (20:3)	0.383	0.413	1.523	0.496	0.291	0.948	0.277	0.280
LPG (20:4)	2.002	2.260	15.137	2.907	2.904	8.243	3.921	2.358
LPG (20:5)	0.141	0.089	0.795	0.159	0.112	0.297	0.124	0.061
LPG (22:4)	0.234	0.462	1.653	0.280	0.422	1.413	0.645	0.350
LPG (22:5)	0.492	0.829	3.876	0.971	0.876	1.730	1.540	0.943
LPG (22:6)	8.585	7.180	75.924	9.172	14.268	20.608	19.921	7.936
LPI (16:0)	13.785	6.644	12.777	7.797	8.266	9.931	4.891	4.319
LPI (16:1)	0.346	0.145	1.563	0.282	0.501	0.652	0.372	0.188
LPI (18:0)	85.108	45.863	57.077	50.424	66.466	92.539	38.460	43.372
LPI (18:1)	2.160	1.239	2.594	1.774	1.665	2.611	1.381	1.123
LPI (18:2)	1.603	0.652	2.246	1.288	0.783	1.546	0.616	0.461
LPI (20:3)	2.018	0.789	2.876	1.868	0.909	2.564	0.632	0.973
LPI (20:4)	17.983	5.916	50.300	11.870	17.898	25.485	13.850	6.431
LPI (20:5)	0.022	0.003	0.090	0.014	0.024	0.074	0.020	0.009
LPI (22:4)	0.077	0.049	0.147	0.052	0.095	0.091	0.034	0.057
LPI (22:6)	1.538	0.112	4.491	1.133	1.527	1.935	1.396	0.649
LPI (36:4)	16.234	14.916	2.309	6.947	9.575	8.448	9.396	6.940
LPI (38:6)	2.067	1.876	0.383	1.356	1.527	1.289	1.282	0.966
LPS (16:0)	6.298	2.155	10.801	3.126	5.298	5.509	2.602	2.238
LPS (18:0)	37.676	13.955	35.542	17.131	28.787	36.497	15.042	14.673
LPS (18:2)	0.594	0.207	1.453	0.432	0.566	0.874	0.350	0.273
LPS (18:3)	0.361	0.158	0.655	0.176	0.406	0.344	0.136	0.128
LPS (20:1)	0.046	0.018	0.018	0.044	0.054	0.032	0.053	0.011
LPS (20:3)	3.283	1.648	6.169	2.178	4.518	5.207	3.074	2.081
LPS (20:4)	8.259	3.064	32.078	6.171	10.075	16.361	8.925	4.454

LPS (20:5)	0.057	0.029	0.304	0.041	0.073	0.157	0.057	0.032
LPS (22:6)	0.785	0.354	4.630	0.715	1.677	2.155	1.209	0.606
MG (18:2)	0.155	0.126	0.113	0.155	0.119	0.151	0.128	0.081
MG (36:4)	0.788	1.002	0.811	1.242	0.544	0.478	0.400	0.298
MG (36:5)	1.018	0.733	0.592	1.350	0.469	0.507	0.417	0.530
MG (36:6)	0.416	0.244	0.201	0.496	0.104	0.204	0.250	0.138
MG (38:4)	0.084	0.183	0.062	0.084	0.035	0.080	0.021	0.097
MG (38:5)	0.658	0.748	0.347	0.523	0.134	0.489	0.186	0.419
PA (16:0)	0.507	0.430	0.409	0.435	0.738	0.522	0.618	0.636
PA (18:1)	0.107	0.085	0.000	0.137	0.026	0.128	0.016	0.033
PA (18:2)	0.107	0.085	0.000	0.137	0.026	0.128	0.016	0.033
PA (22:6)	0.507	0.430	0.409	0.435	0.738	0.522	0.618	0.636
PC (14:0)	1.112	1.351	0.932	0.856	0.672	0.884	0.645	0.852
PC (16:0)	151.256	149.589	135.006	133.577	135.110	141.678	129.591	125.568
PC (16:1)	7.283	7.230	7.582	6.749	7.958	8.177	7.987	7.462
PC (16:2)	0.067	0.068	0.049	0.076	0.029	0.063	0.036	0.019
PC (18:0)	104.328	104.039	96.762	97.625	97.219	102.122	98.486	95.125
PC (18:1)	16.970	18.263	13.839	13.406	16.126	16.327	13.537	16.332
PC (18:2)	28.004	27.176	29.029	31.056	31.982	34.865	30.201	31.104
PC (18:3)	0.623	0.466	0.717	0.442	0.803	0.823	0.643	0.504
PC (18:4)	7.448	7.640	8.110	8.179	9.440	10.150	10.569	8.768
PC (20:0)	0.530	0.548	0.541	0.539	0.711	0.772	0.830	0.789
PC (20:1)	0.343	0.483	0.413	0.483	0.449	0.528	0.597	0.610
PC (20:2)	0.972	1.082	0.981	0.791	0.991	1.127	1.101	1.002
PC (20:3)	1.418	1.358	1.534	1.693	1.942	1.861	1.722	1.986
PC (20:4)	23.546	23.920	28.053	23.842	34.519	33.856	42.084	32.393
PC (20:5)	9.023	8.515	12.140	9.449	13.456	10.655	14.630	11.290
PC (22:0)	0.045	0.098	0.086	0.053	0.065	0.113	0.104	0.097
PC (22:1)	0.037	0.092	0.010	0.032	0.016	0.029	0.069	0.059
PC (22:3)	1.036	1.079	0.797	0.863	0.864	1.550	0.903	0.955
PC (22:4)	6.089	6.137	6.667	6.003	7.073	6.745	7.054	6.870
PC (22:5)	0.893	0.758	1.249	0.796	1.187	0.450	1.151	1.009
PC (22:6)	5.408	4.277	7.969	5.981	8.390	5.530	6.966	5.916
PC (24:0)	0.021	0.047	0.025	0.035	0.047	0.036	0.055	0.060

PC (24:1)	0.005	0.011	0.007	0.005	0.006	0.008	0.011	0.013
PC (24:7)	1.155	1.435	1.172	1.022	1.039	1.039	1.087	1.049
PE (14:0)	0.189	0.155	0.165	0.167	0.160	0.157	0.167	0.147
PE (16:0)	415.989	460.973	161.422	431.327	416.373	340.419	348.550	434.809
PE (16:1)	6.693	4.226	2.624	4.904	3.280	3.317	3.744	3.487
PE (18:0)	602.457	576.332	169.993	590.895	616.780	477.919	600.176	567.502
PE (18:1)	515.370	499.824	130.845	533.178	484.852	414.166	378.405	532.322
PE (18:2)	97.994	105.330	31.109	100.067	71.830	73.998	71.046	83.781
PE (18:3)	0.060	0.045	0.045	0.049	0.039	0.042	0.042	0.043
PE (20:0)	9.670	5.874	2.388	6.339	6.534	7.057	8.028	6.726
PE (20:1)	6.823	8.954	11.367	6.970	7.348	6.864	6.824	8.093
PE (20:2)	2.581	3.006	7.487	3.613	2.637	3.577	2.489	2.892
PE (20:3)	97.719	51.299	25.657	54.874	59.123	43.860	60.880	58.147
PE (20:4)	903.869	890.826	274.820	933.449	927.532	728.680	840.267	923.383
PE (20:5)	1.232	2.074	0.295	1.576	1.120	1.092	1.045	1.511
PE (22:4)	12.602	17.017	6.538	12.271	12.983	16.541	15.672	13.206
PE (22:5)	14.898	16.003	10.115	16.884	17.947	15.611	17.169	18.049
PE (22:6)	378.080	419.067	110.584	421.365	384.752	307.728	279.290	400.573
PG (14:0)	0.186	0.110	0.161	0.341	0.070	0.073	0.111	0.080
PG (16:0)	26.616	25.330	14.035	28.106	16.538	55.399	40.631	22.432
PG (16:1)	1.412	1.274	1.370	1.530	0.794	1.153	0.738	0.870
PG (16:2)	0.142	0.133	0.220	0.138	0.046	0.079	0.034	0.063
PG (18:0)	7.265	8.550	8.420	7.628	7.911	42.817	33.236	7.138
PG (18:1)	4.858	6.363	3.495	3.694	2.462	3.522	2.000	3.829
PG (18:2)	1.204	1.603	1.105	1.120	0.480	0.983	0.487	0.758
PG (18:3)	4.906	5.234	6.587	5.074	6.869	6.919	6.710	6.172
PG (18:4)	0.015	0.011	0.013	0.018	0.004	0.008	0.003	0.006
PG (20:2)	17.821	15.194	2.610	18.446	6.946	10.698	5.262	13.476
PG (20:3)	1.826	2.238	1.730	1.804	1.128	1.534	1.234	1.398
PG (20:4)	2.882	3.166	2.002	2.536	1.755	2.272	1.925	2.316
PG (22:4)	0.476	1.067	0.773	0.316	0.327	0.873	0.539	0.573
PG (22:5)	3.890	4.475	5.381	4.070	6.199	6.078	6.180	5.728
PG (22:6)	2.355	3.186	2.818	1.747	1.767	2.198	2.181	2.767
PI (16:0)	51.129	37.724	14.520	35.164	40.739	32.767	38.799	27.901

PI (16:1)	3.174	3.236	2.634	2.981	5.620	4.444	4.784	3.586
PI (18:0)	496.955	226.556	24.213	167.982	291.459	264.185	279.339	152.848
PI (18:1)	20.218	17.245	6.225	17.709	17.729	20.158	19.085	13.917
PI (18:2)	5.888	4.642	1.035	5.549	4.296	4.840	4.182	2.915
PI (18:3)	0.170	0.120	0.041	0.171	0.125	0.148	0.164	0.111
PI (20:3)	0.415	0.310	0.367	0.404	0.299	0.383	0.163	0.450
PI (20:4)	546.317	262.361	31.355	200.860	325.884	293.969	313.459	175.947
PI (22:3)	0.246	0.281	1.216	0.238	0.552	0.975	0.431	0.361
PI (22:5)	3.003	3.024	0.161	2.388	4.036	2.735	4.439	2.545
PI (22:6)	2.436	2.153	1.602	1.809	2.042	1.720	1.785	1.861
PS (16:0)	15.559	14.507	16.094	15.042	20.430	17.505	17.035	18.178
PS (18:0)	22.270	22.451	25.681	22.617	30.892	34.592	30.012	29.155
PS (18:1)	3.638	4.178	5.668	4.035	4.906	6.595	4.925	5.147
PS (18:2)	6.700	6.055	4.624	8.289	9.669	9.296	9.985	7.026
PS (18:3)	1.578	1.321	0.930	1.530	1.582	1.713	1.547	1.182
PS (20:0)	0.056	0.134	0.224	0.164	0.180	0.222	0.300	0.405
PS (20:2)	19.259	19.628	8.217	24.048	25.980	23.952	29.915	21.137
PS (20:3)	9.765	10.541	8.851	10.927	15.957	15.809	15.491	12.766
PS (20:4)	46.189	44.395	31.378	52.867	60.250	60.102	63.511	51.645
PS (20:5)	0.863	0.999	1.915	0.972	0.800	1.058	0.869	1.161
PS (22:0)	0.125	0.180	0.161	0.112	0.152	0.474	0.322	0.165
PS (22:4)	1.116	1.439	1.409	1.033	1.451	2.223	2.000	1.358
PS (22:5)	0.302	0.339	0.268	0.242	0.359	0.488	0.318	0.362
PS (22:6)	8.759	9.541	9.555	8.468	16.807	14.197	15.345	13.276
PS (24:0)	0.041	0.125	0.029	0.035	0.045	0.154	0.093	0.066
PS (24:5)	0.628	0.483	0.490	0.507	0.432	0.345	0.294	0.407
PS (24:6)	0.010	0.004	0.006	0.004	0.002	0.005	0.003	0.003
PS (24:7)	0.038	0.042	0.059	0.045	0.072	0.072	0.076	0.060
TG (10:0)	0.074	0.042	0.021	0.039	0.019	0.011	0.002	0.011
TG (12:0)	3.545	2.344	1.918	3.316	1.801	1.075	0.647	1.064
TG (14:0)	31.831	24.752	15.565	28.720	7.796	10.650	3.923	9.682
TG (14:1)	1.388	0.547	0.373	0.985	0.180	0.186	0.063	0.169
TG (16:0)	261.767	229.891	161.956	264.105	141.584	106.889	72.380	128.258
TG (16:1)	25.301	19.723	14.177	23.372	9.191	9.242	4.953	9.717

TG (16:2)	4.207	2.148	1.909	3.464	0.914	1.421	0.809	1.302
TG (18:0)	34.127	43.988	36.410	33.834	45.161	23.085	20.159	31.659
TG (18:1)	188.113	218.632	185.490	204.064	243.387	115.457	106.251	142.968
TG (18:2)	194.143	150.960	117.544	184.873	101.344	79.988	48.317	84.332
TG (18:3)	29.677	18.989	14.288	31.524	11.416	9.578	4.404	9.973
TG (18:4)	3.378	0.770	0.609	1.428	0.487	0.656	0.370	0.349
TG (20:0)	3.628	3.754	1.704	3.027	0.564	1.601	0.611	2.530
TG (20:1)	13.423	13.426	8.502	13.818	5.306	7.850	5.302	9.982
TG (20:2)	3.646	3.896	2.321	3.310	0.850	2.115	1.140	2.708
TG (20:3)	4.381	5.999	3.195	3.308	1.610	3.258	2.296	3.648
TG (20:4)	8.147	7.317	6.441	8.407	6.403	4.715	4.005	5.810
TG (20:5)	4.950	4.013	3.197	5.044	2.561	2.165	1.344	2.237
TG (22:0)	0.118	0.159	0.143	0.309	0.080	0.084	0.079	0.190
TG (22:1)	1.554	1.965	1.206	1.558	0.494	0.994	0.824	1.406
TG (22:4)	6.388	6.422	5.390	7.053	3.896	4.760	2.643	5.476
TG (22:5)	7.081	8.206	4.821	6.399	2.764	3.892	2.015	4.602
TG (22:6)	42.206	43.648	27.113	32.162	9.419	24.062	11.957	26.531
TG (24:0)	0.340	0.349	0.330	0.565	0.138	0.162	0.236	0.527
TG (24:1)	0.206	0.299	0.192	0.302	0.059	0.163	0.156	0.267
TG (24:6)	0.177	0.161	0.127	0.116	0.039	0.090	0.124	0.104
TG (26:0)	0.027	0.032	0.021	0.053	0.005	0.008	0.019	0.044
TG (26:1)	0.026	0.046	0.039	0.044	0.005	0.005	0.007	0.059
TG (4:0)	0.009	0.088	0.072	0.021	0.146	0.018	0.004	0.011
TG (6:0)	0.024	0.048	0.024	0.024	0.057	0.006	0.002	0.005
TG (8:0)	4.963	3.820	2.800	5.862	2.340	2.519	1.962	2.724

Data are presented as relative abundance (normalized peak area) of individual lipid species in the renal cortex of diabetic *C5ar2*$^{-/-}$ mice (n = 4) and diabetic WT mice (n = 4). Abbreviations: CL, cardiolipin; ChE, cholesteryl ester; DG, diacylglycerol; FA, fatty acid; LPA, lysophosphatidic acid; LPC, lysophosphatidylcholine; LPE, lysophosphatidylethanolamine; LPG, lysophosphatidylglycerol; LPI, lysophosphatidylinositol; LPS, lysophosphatidylserine; MG, monoacylglycerol; PA, phosphatidic acid; PC, phosphatidylcholine; PE, phosphatidylethanolamine; PG, phosphatidylglycerol; PI, phosphatidylinositol; PS, phosphatidylserine; TG, triacylglycerol.

Supplementary Table S4. Laboratory data of PSS2-specific overexpression in proximal tubular epithelial cells from *C5ar2*$^{-/-}$ mice.

Variables	STZ/HFD diabetic mice			
	WT mice		*C5ar2*$^{-/-}$ mice	
	OE Ctrl	OE PSS2	OE Ctrl	OE PSS2
**BW (g)**	21.70 ± 1.60	26.04 ± 1.02***	27.36 ± 2.03**	30.53 ± 2.22$^{\#\#}$
**FBG (mmol/l)**	26.00 ± 2.51	24.53 ± 2.84	25.51 ± 2.14*	25.68 ± 2.60
**uACR (μg/mg)**	154.90 ± 22.07	93.33 ± 19.15**	248.40 ± 37.31***	128.2 ± 29.36$^{\#\#\#\#}$
**TG (mmol/l)**	2.08 ± 0.45	2.67 ± 0.52	3.80 ± 0.89**	3.42 ± 0.91
**TCHO (mmol/l)**	4.2 ± 0.39	4.06 ± 1.15	4.06 ± 1.15	4.34 ± 0.49

BW, body weight; FBG, fasting blood glucose; uACR, urine albumin-to-creatinine ratio; TG, triglyceride; TCHO, total cholesterol. The continuous data are expressed as the means ± SDs. The data were analysed by two-sided one-way ANOVA with Tukey's test. * $P < 0.05$ vs. STZ/HFD-WT-OE Ctrl; ** $P < 0.01$ vs. STZ/HFD-WT-OE Ctrl; *** $P < 0.001$ vs. STZ/HFD-WT-OE Ctrl; $^{\#\#}$ $P < 0.01$ vs. STZ/HFD-*C5ar2*$^{-/-}$-OE Ctrl; $^{\#\#\#\#}$ $P < 0.0001$ vs. STZ/HFD-*C5ar2*$^{-/-}$-OE Ctrl.

Supplementary Table S5. Laboratory data for *db/db* mice treated with P59 dose gradients.

Variables	*m/m* mice	*db/db* mice			
	vehicle	vehicle	P59-1 mg/kg	P59-3 mg/kg	P59-5 mg/kg
**BW (g)**	25.78 ± 3.65	47.70 ± 10.44^{****}	45.37 ± 4.59	38.48 ± 3.95	31.25 ± $4.59^{\#\#\#}$
**FBG (mmol/l)**	7.37 ± 1.45	27.23 ± 6.04^{****}	28.72 ± 3.91	26.78 ± 4.64	28.75 ± 3.54
**uACR (μg/mg)**	41.09 ± 12.51	1008.00 ± 523.50^{***}	360.50 ± $335.60^{\#\#}$	254.10 ± $176.40^{\#\#}$	207.20 ± $165.0^{\#\#\#}$
**TG (mmol/l)**	1.88 ± 0.31	10.36 ± 4.78^{****}	5.57 ± $1.26^{\#}$	5.04 ± $1.20^{\#\#}$	4.79 ± $1.90^{\#\#}$
**TCHO (mmol/l)**	3.06 ± 0.50	4.21 ± 1.04	3.86 ± 0.32	4.37 ± 1.08	3.90 ± 0.44

BW, body weight; FBG, fasting blood glucose; uACR, urine albumin-to-creatinine ratio; TG, triglyceride; TCHO, total cholesterol. The continuous data are expressed as the means ± SDs. The data were analysed by two-sided one-way ANOVA with Tukey's test. *** $P < 0.001$ vs. *m/m*-vehicle; **** $P < 0.0001$ vs. *m/m*-vehicle; $^{\#}$ $P < 0.05$ vs. *db/db*-vehicle; $^{\#\#}$ $P < 0.01$ vs. *db/db*-vehicle; $^{\#\#\#}$ $P < 0.001$ vs. *db/db*-vehicle.

Supplementary Table S6. Laboratory data of P59 (3 mg/kg)-treated *db/db* mice.

Variables	STZ/HFD diabetic mice			
	***m/m* mice**		***db/db* mice**	
	**vehicle**	**P59-3 mg/kg**	**vehicle**	**P59-3 mg/kg**
**BW (g)**	26.68 ± 1.81	23.31 ± 2.43	46.94 ± 10.97****	33.13 ± 5.74$^{\#\#\#}$
**FBG (mmol/l)**	7.17 ± 1.27	7.86 ± 0.94	26.57 ± 3.56****	28.06 ± 2.00
**uACR (µg/mg)**	208.70 ± 77.44	201.60 ± 64.55	2340.00 ± 973.30****	1154.00 ± 258.10$^{\#\#\#}$
**TG (mmol/l)**	0.75 ± 0.11	0.74 ± 0.14	1.54 ± 0.34****	1.12 ± 0.20$^{\#\#}$
**TCHO (mmol/l)**	3.51 ± 0.58	3.27 ± 0.55	5.84 ± 1.72***	5.64 ± 0.78

BW, body weight; FBG, fasting blood glucose; uACR, urine albumin-to-creatinine ratio; TG, triglyceride; TCHO, total cholesterol. The continuous data are expressed as the means ± SDs. *** $P < 0.001$ vs. *m/m*-vehicle; **** $P < 0.0001$ vs. *m/m*-vehicle; $^{\#\#}$ $P < 0.01$ vs. *db/db*-vehicle; $^{\#\#\#}$ $P < 0.001$ vs. *db/db*-vehicle.

Supplementary Table S7. Primers used in this study.

Name	Sequence
**Genotyping primers**	
C5ar2	F: 5’-GCCTGCTTTGTGTCTTGTGTGTG-3’<br>R: 5’-AAAGTTATCACCACCGTGCCTGG-3’
**siRNA targets**	
C5ar2	5’-CACGAAATGGTGTCTGAGA-3’
Pss1	5’-CTGAACTTCGAGCAGGTTA-3’
Pss2	5’-CTACCTGAAGTTTGTGCTA-3’
**qRT-PCR primers**	
C5ar2	F: 5’-CACACCACCAGCGAGTATTATG-3’<br>R: 5’-AGCACAAGCAGGACTATCAGG-3’
Pss1	F: 5’-GCAGGACTCTGAGCAAGGATG-3’<br>R: 5’-GGCGAAGTACATGAGGCTGAT-3’
Pss2	F: 5’-GGATTGCCTTTCAGTTCACGC-3’<br>R: 5’-AGGTAGAAGGTGTTCAGCTCTG-3’
Tgfb1	F: 5’-CTCCCGTGGCTTCTAGTGC-3’<br>R: 5’-GCCTTAGTTTGGACAGGATCTG-3’
F4/80	F: 5’-TGACTCACCTTGTGGTCCTAA-3’<br>R: 5’-CTTCCCAGAATCCAGTCTTTCC-3’
Acta1	F: 5’-AACGCTTCCGCTGCCC-3’<br>R: 5’-CGTGGATGCCCGCTGA-3’
Lcn2	F: 5’-TGGCCCTGAGTGTCATGTG-3’<br>R: 5’-CTCTTGTAGCTCATAGATGGTGC-3’
c-Fos	F: 5’-CGGGTTTCAACGCCGACTA-3’<br>R: 5’-TTGGCACTAGAGACGGACAGA-3’
18 s	F: 5’-GTAACCCGTTGAACCCCATT-3’<br>R: 5’-CCATCCAATCGGTAGTAGCG-3’

Supplementary Table S8. Antibodies used in this study.

Name	Source	Identifier
Rabbit monoclonal to alpha smooth muscle Actin (acetyl E3) + ACTG2 (acetyl E3) antibody [E184]	Abcam	ab32575<br>RRID: AB_722538
Rat monoclonal to F4/80 (Cl: A3-1)	Bio-Rad	MCA497<br>RRID: AB_2098196
Rabbit monoclonal to TGFBI antibody	Abcam	ab170874<br>RRID: AB_2895231
Rabbit polyclonal to Perilipin2	Abcam	ab52356<br>RRID: AB_2223599
Rabbit monoclonal [EPR8118(2)] to PTDSS1	Abcam	ab157222<br>RRID: AB_3105777
Rabbit polyclonal PTDSS2 Antibody-middle region	Aviva Systems Biology Corporation	ARP49961_P050<br>RRID: AB_10713141
Mouse monoclonal [EPR8118(2)] to C5aR2	Santa Cruz Biotechnology	sc-515734
Rabbit polyclonal to C5aR2 antibody	ABclonal	A10588<br>RRID: AB_2758129
Rabbit monoclonal [EPR11588(B)] to Aquaporin 1	Abcam	ab168387<br>RRID: AB_2810992
Rabbit polyclonal to Calbindin-D28k Antibody	Proteintech	14479-1-AP<br>RRID: AB_2228318
Rabbit polyclonal to Synaptopodin (D-9) Antibody	Santa Cruz Biotechnology	sc-515842<br>RRID: AB_2921309
Rabbit anti MFN2 polyclonal antibody	Proteintech	12186-1-AP<br>RRID: AB_2266320
HRP-conjugated Alpha Tubulin Recombinant antibody	Proteintech	HRP-80762<br>RRID: AB_3673982
Rabbit polyclonal to EIF2α antibody	Proteintech	11170-1-AP<br>RRID: AB_2096489
Rabbit polyclonal to CHOP antibody	Proteintech	15204-1-AP<br>RRID: AB_2292610

Mouse polyclonal to XBP-1 s antibody	Biolegend	143F
Rabbit polyclonal to p-EIF2α antibody	Proteintech	28740-1-AP<br>RRID: AB_2881204
Mouse monoclonal to DYKDDDDK tag antibody	Proteintech	66008-4-Ig<br>RRID: AB_2918475
Rat monoclonal to DYKDDDDK tag antibody	Abcam	ab213519
Normal mouse IgG	Santa Cruz Biotechnology	sc-2025<br>RRID: AB_737182
Normal rabbit IgG	Cell Signaling Technology	2729<br>RRID: AB_1031062
Mouse polyclonal to VDAC1/Porin antibody	Proteintech	66345-1-Ig<br>RRID: AB_2881725
Rabbit polyclonal to ITPR1-specific antibody	Proteintech	19962-1-AP<br>RRID: AB_10638786
Rabbit monoclonal to c-Fos (9F6) antibody	Cell Signaling Technology	2250<br>RRID: AB_2247211
Rabbit monoclonal to Histone H3 (D1H2) antibody	Cell Signaling Technology	4499<br>RRID: AB_10544537
Rabbit polyclonal to Calnexin antibody	Abcam	ab22595<br>RRID: AB_2069006
Rabbit polyclonal to COXIV antibody	Proteintech	11242-1-AP<br>RRID: AB_2085278
HRP-conjugated Goat anti-Rabbit IgG (H+L)	Proteintech	SA00001-2<br>RRID: AB_2722564
HRP-conjugated Goat anti-Mouse IgG (H+L)	Proteintech	SA00001-1<br>RRID: AB_2722565
Alexa Fluor 594-conjugated donkey anti-rabbit IgG	Invitrogen	A21207<br>RRID: AB_141637
Alexa Fluor 488-conjugated goat anti-mouse IgG	Invitrogen	A32723<br>RRID: AB_2534069

Rabbit monoclonal to p44/42 MAPK (Erk1/2) (137F5) Antibody	Cell Signaling Technology	4695<br>RRID: AB_390779
Rabbit monoclonal to Phospho-p44/42 MAPK (Erk1/2) (Thr202/Tyr204) Antibody	Cell Signaling Technology	4370<br>RRID: AB_2315112
Rabbit polyclonal antibody to HA tag	Abcam	ab9110<br>RRID: AB_307019
HRP-conjugated β-Actin Rabbit monoclonal antibody	Abclonal	AC028<br>RRID: AB_2769861

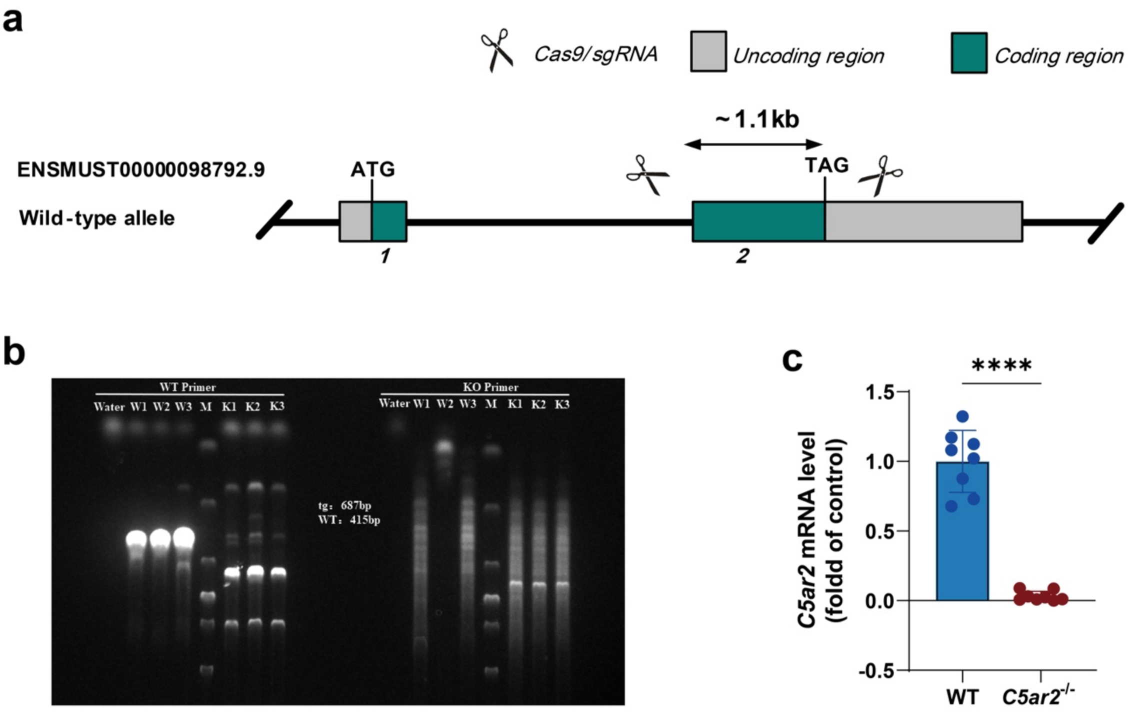

Supplementary Figure S1. Identification of *C5ar2*$^{-/-}$ mice. (a) The coding region of the *C5ar2*-201 (ENSMUST00000098792.9) transcript exon 2, which was approximately 1.1 kb, was selected as the knockout region. (b) Agarose gel electrophoresis image of genotype identification for *C5ar2*$^{-/-}$ mice and wild-type (WT) mice. (c) Quantitative real-time PCR (qRT-PCR) analysis showing *C5ar2* mRNA levels in the kidneys of standard fat diet (SFD) *C5ar2*$^{-/-}$ and SFD WT mice ($n = 8$ per group). The data in the graphs are presented as the means ± SDs. The data were analysed via an unpaired two-tailed Student's t test. ****$P < 0.0001$.

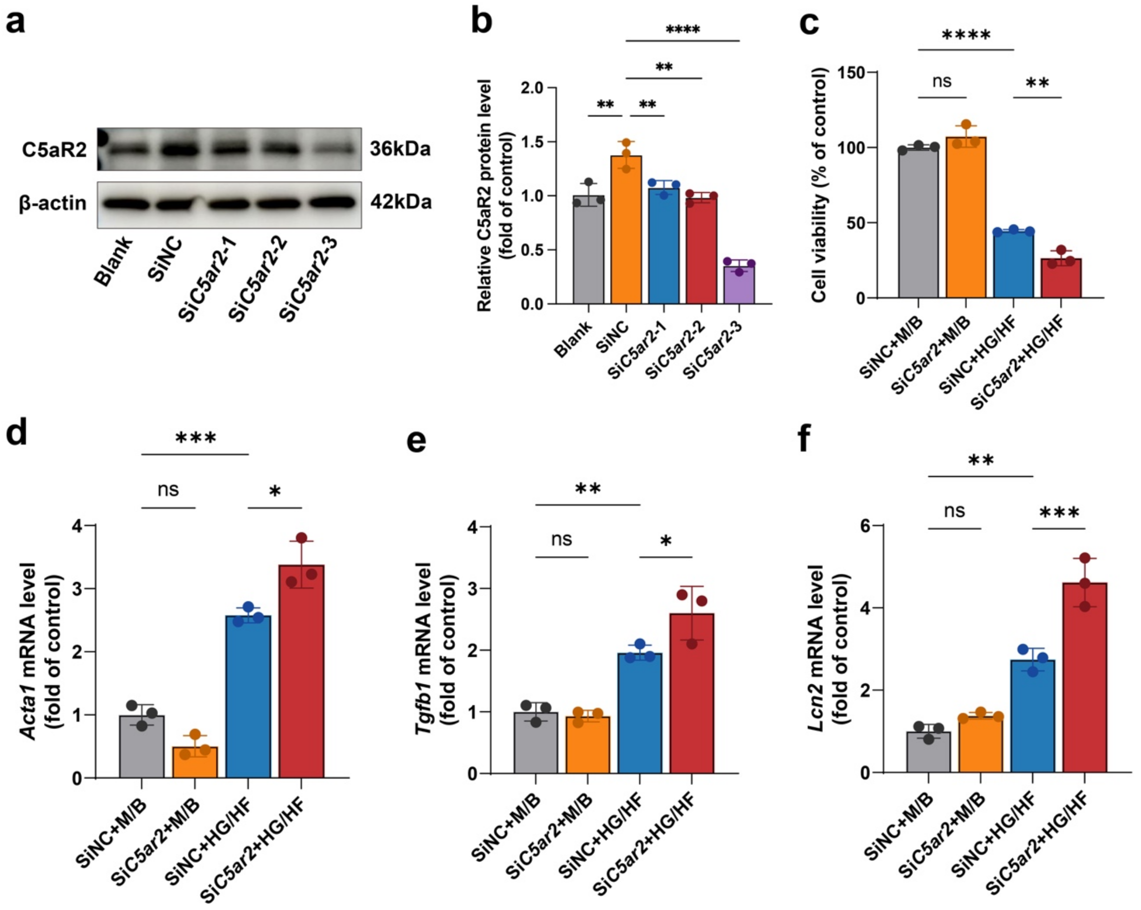

Supplementary Figure S2. *C5ar2* knockdown significantly increased the expression of injury and fibrosis markers in HG/HF-treated TCMK-1 cells. (a and b) Western blot images and quantitative analysis of the knockdown efficiency of three different siRNAs targeting *C5ar2* in TCMK-1 cells (n = 3 independent replicates). (c) CCK8 assays revealed that *C5ar2* knockdown significantly reduced the viability of HG/HF-treated TCMK-1 cells (n = 3 independent replicates). (d-f) qRT-PCR analysis showing the relative *Acta1*, *Tgfb1 and Lcn2* mRNA levels in TCMK-1 cells subjected to different treatments (n = 3 independent replicates). The data in the graphs are presented as the means ± SDs. The data were analysed by two-sided one-way ANOVA with Tukey's test. ns, not significant; *$P < 0.05$; **$P < 0.01$; ***$P < 0.001$; ****$P < 0.0001$.

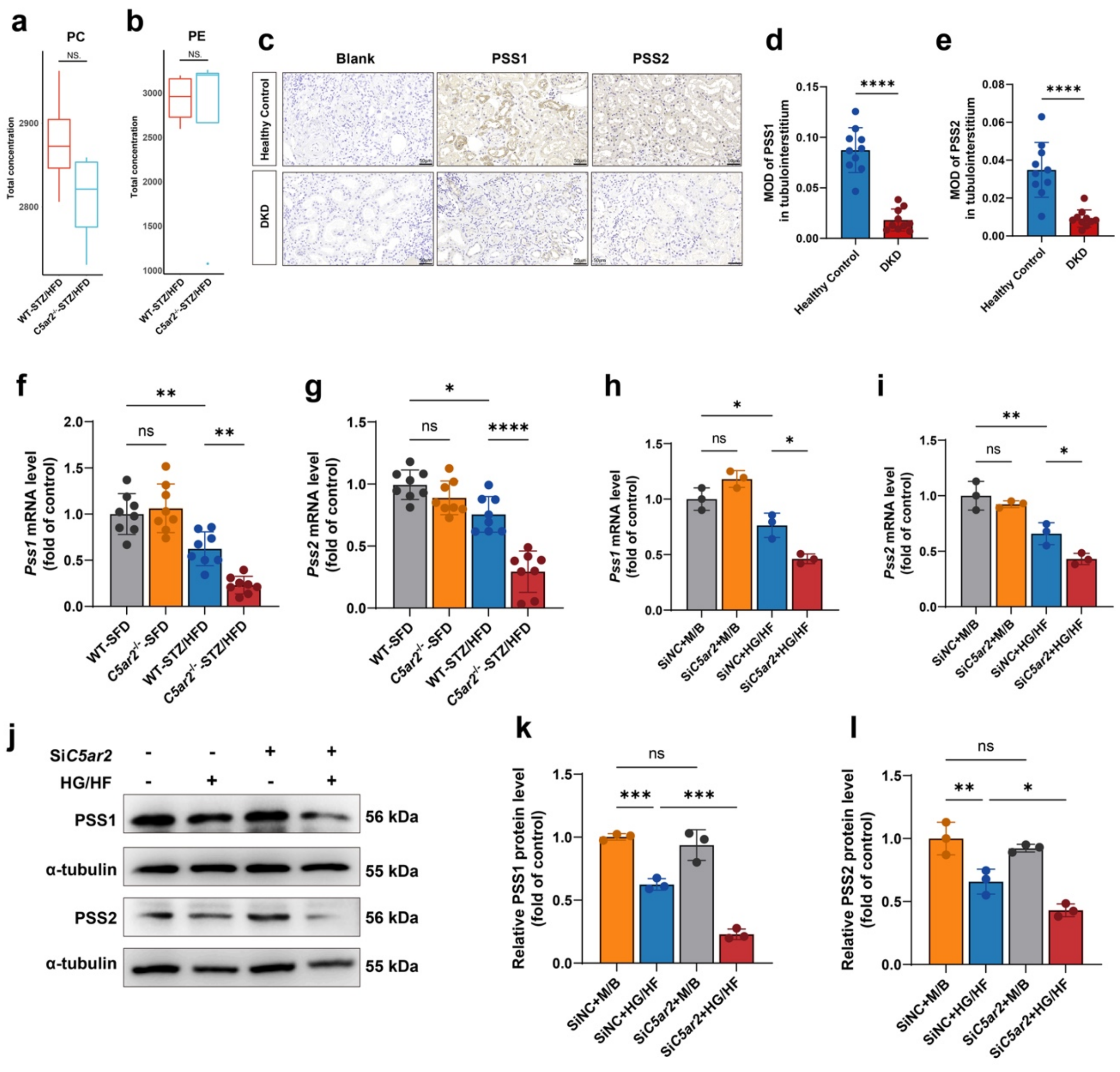

Supplementary Figure S3. C5aR2 is required for phospholipid biosynthesis and PSS expression in TECs in DKD. (a) Total phosphatidylcholine (PC) levels in the renal cortex of diabetic *C5ar2*$^{-/-}$ mice and diabetic WT mice (n = 4 per group). (b) Total phosphatidylethanolamine (PE) levels in the renal cortex of diabetic *C5ar2*$^{-/-}$ mice and diabetic WT mice (n = 4 per group). (c-e) Representative IHC images and quantitative analysis of PSS1 and PSS2 expression levels in kidney tissues from DKD patients (n = 10 each) and healthy control kidney tissues (n = 10 each) (Scale bars = 50 μm). MOD: mean optical density. (f and g) qRT-PCR analysis showing relative *Pss1* and *Pss2* mRNA levels in different groups of mice (n = 8 per group). (h and i) qRT-PCR analysis showing relative

Pss1 and *Pss2* mRNA levels in TCMK-1 cells subjected to different treatments (n = 3 independent replicates). (j-l) Representative Western blot images and quantitative analysis of PSS1 and PSS2 expression in TCMK-1 cells subjected to different treatments (n = 3 independent replicates). The data in the graphs are presented as the means ± SDs. The data were analysed via an unpaired two-tailed Student's t test (a, b, d and e) and two-sided one-way ANOVA with Tukey's test (f-i, k and l). ns, not significant; $*P < 0.05$; $**P < 0.01$; $***P < 0.001$; $****P < 0.0001$.

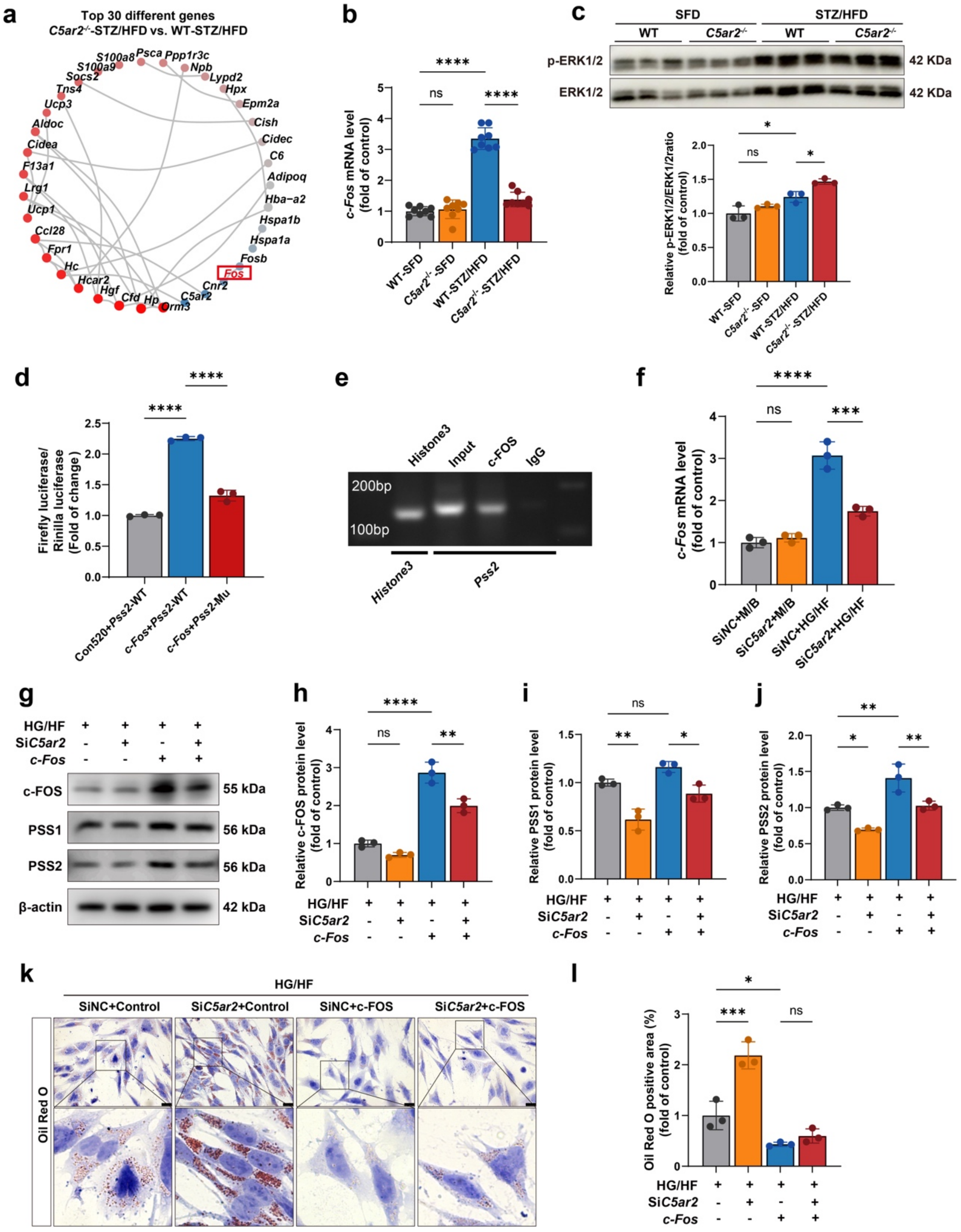

Supplementary Figure S4. C5aR2 regulates PSS expression through c-FOS in TECs in DKD.

(a) Top 30 differentially expressed genes (DEGs) in the renal cortex tissue transcriptomes of diabetic *C5ar2*$^{-/-}$ mice and diabetic WT mice (n = 4 per group). The second significantly downregulated gene,

c-Fos, is highlighted with red boxes. (b) qRT-PCR analysis showing the relative *c-Fos* mRNA levels in different groups of mice (n = 8 per group). (c) Representative Western blot images and quantitative analysis of ERK1/2 phosphorylation (p-ERK1/2/ERK1/2) in the renal cortex of diabetic *C5ar2*$^{-/-}$ mice and diabetic WT mice (n = 3 per group). (d) Luciferase reporter assays of *c-Fos*-overexpressing TCMK-1 cells transfected with a reporter plasmid containing the PSS2 promoter with or without mutation at the c-FOS binding site (n = 3 independent replicates). (e) Total protein lysates from cells were subjected to immunoprecipitation using an anti-c-FOS antibody, followed by chromatin immunoprecipitation (ChIP) analysis of TCMK-1 cells via primers targeting the putative regulatory region of the *Pss2* promoter. (f) qRT-PCR analysis showing *c-Fos* mRNA levels in TCMK-1 cells under different treatments (n = 3 independent replicates). (g and h) Western blot images and quantitative analysis of c-FOS protein expression in TCMK-1 cells under different treatments (n = 3 independent replicates). (i and j) Western blot images and quantitative analysis of PSS1 and PSS2 expression in c-FOS-overexpressing TCMK-1 cells with *C5ar2* knockdown under HG/HF conditions (n = 3 independent replicates). (k and l) Representative images and quantitative analysis of Oil Red O staining in c-FOS-overexpressing TCMK-1 cells with *C5ar2* knockdown under HG/HF conditions (n = 3 independent replicates) (Scale bars = 50 μm). The data in the graphs are presented as the means ± SDs. The data were analysed via two-sided one-way ANOVA with Tukey's test. ns, not significant; *$P < 0.05$; **$P < 0.01$; ***$P < 0.001$; ****$P < 0.0001$.

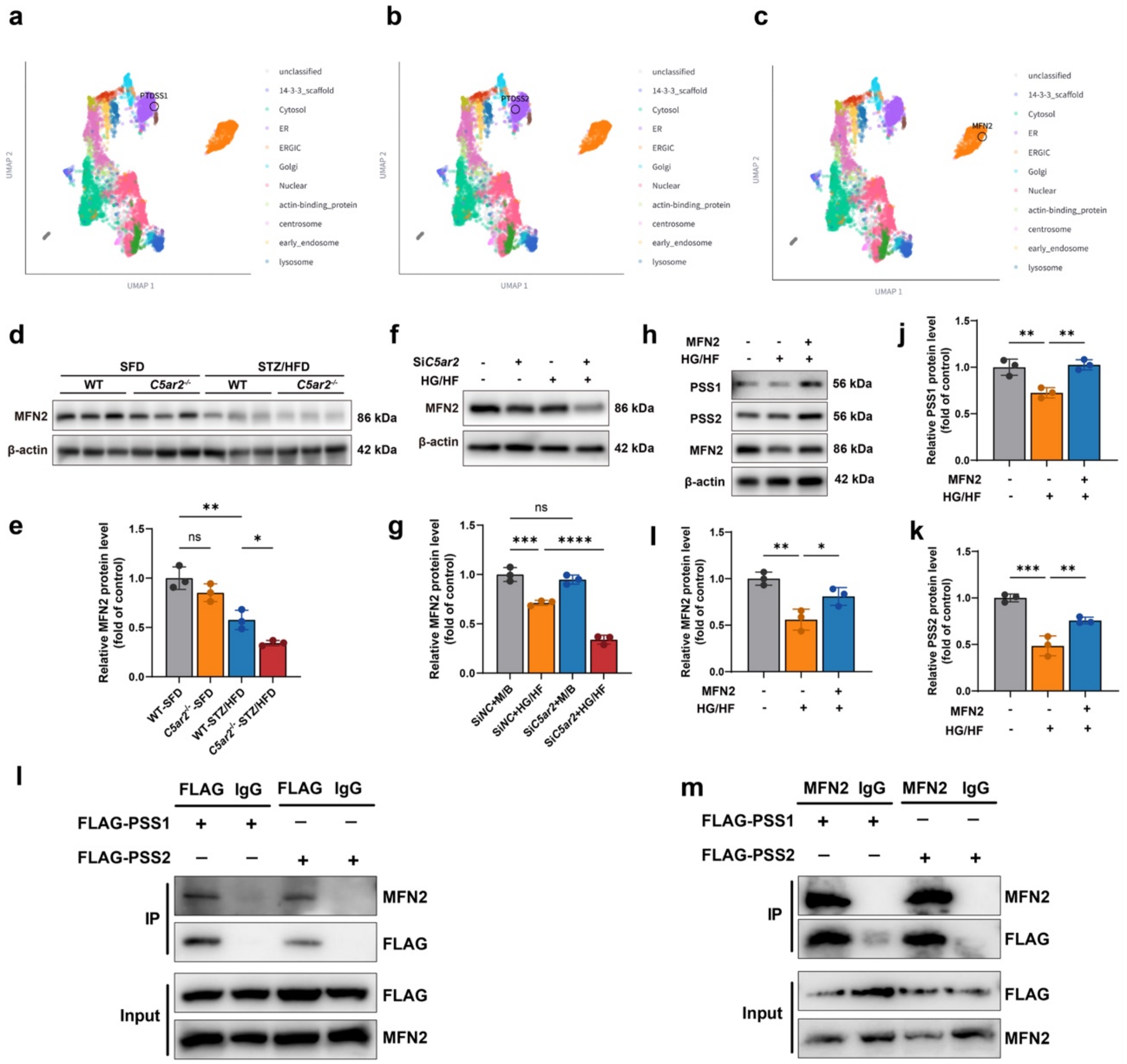

Supplementary Figure S5. Subcellular localization of PSS1/2 and MFN2 and functional regulation of MFN2 and PSS1/2 by C5aR2. (a and b) Subcellular proteomics of the organelle IP of HEK293T human cell lines revealed that the localization of PSS1 and PSS2 was mainly concentrated in the ER. (c) Subcellular proteomics of the organelle IPs of HEK293T human cell lines revealed that MFN2 was mainly concentrated in the mitochondria. (d and e) Representative Western blot images and quantitative analysis of MFN2 expression in the renal cortex of diabetic *C5ar2*$^{-/-}$ mice and diabetic WT mice (n = 3 per group). (f and g) Representative Western blot images and quantitative analysis of MFN2 expression in TCMK-1 cells with *C5ar2* knockdown under HG/HF conditions (n

= 3 independent replicates). (h-k) Representative Western blot images and quantitative analysis of PSS1, PSS2 and MFN2 expression in MFN2-overexpressing TCMK-1 cells under HG/HF conditions (n = 3 independent replicates). (l) Western blot images showing coimmunoprecipitation (Co-IP) of overexpressed FLAG-PSS1 and endogenous MFN2 in TCMK-1 cells. (m) Western blot images showing Co-IP of overexpressed FLAG-PSS2 and endogenous MFN2 in TCMK-1 cells. The data in the graphs are presented as the means ± SDs. The data were analysed via two-sided one-way ANOVA with Tukey's test. ns, not significant; $*P < 0.05$; $**P < 0.01$; $***P < 0.001$; $****P < 0.0001$.

159

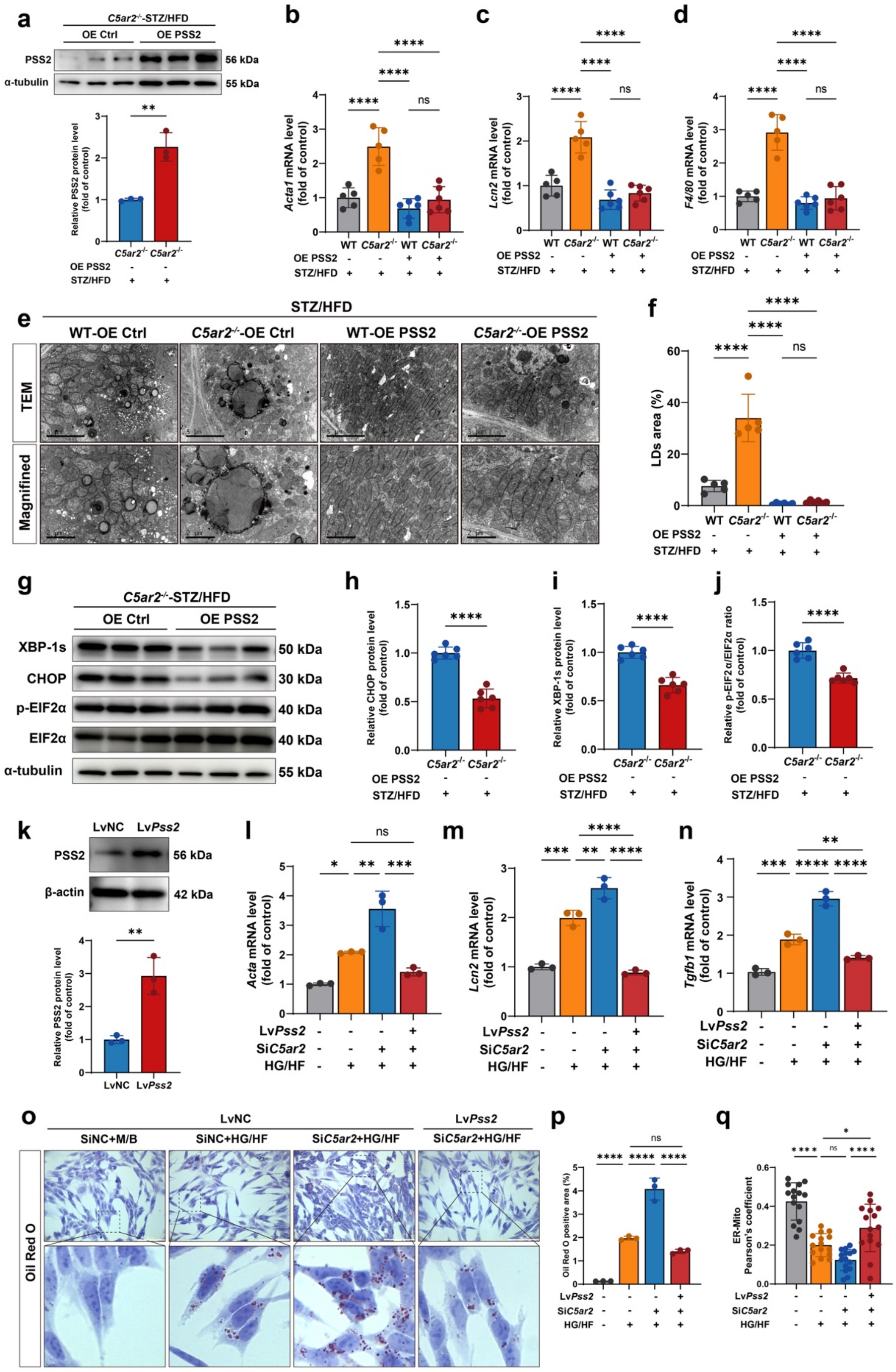

a
C5ar2-/--STZ/HFD
OE Ctrl
OE PSS2
PSS2
α-tubulin
56 kDa
55 kDa
Relative PSS2 protein level (fold of control)
b
Acta1 mRNA level (fold of control)
c
Lcn2 mRNA level (fold of control)
d
F4/80 mRNA level (fold of control)
WT C5ar2-/- WT C5ar2-/-
OE PSS2
STZ/HFD
e
STZ/HFD
WT-OE Ctrl
C5ar2-/--OE Ctrl
WT-OE PSS2
C5ar2-/--OE PSS2
TEM
Magnified
f
LDs area (%)
g
C5ar2-/--STZ/HFD
XBP-1s
CHOP
p-EIF2α
EIF2α
50 kDa
30 kDa
40 kDa
h
Relative CHOP protein level (fold of control)
i
Relative XBP-1s protein level (fold of control)
j
Relative p-EIF2α/EIF2α ratio (fold of control)
k
LvNC
LvPss2
β-actin
42 kDa
l
Acta mRNA level (fold of control)
m
Lcn2 mRNA level (fold of control)
n
Tgfb1 mRNA level (fold of control)
LvPss2
SiC5ar2
HG/HF
o
SiNC+M/B
SiNC+HG/HF
SiC5ar2+HG/HF
Oil Red O
p
Oil Red O positive area (%)
q
ER-Mito Pearson's coefficient

Supplementary Figure S6. PSS2 overexpression ameliorated lipid accumulation, ER stress, and impaired MAM formation induced by C5aR2 deficiency. (a) Representative Western blot images and quantitative analysis of PSS2 expression efficiency in renal cortex tissue from STZ/HFD-WT + OE Ctrl mice and STZ/HFD-WT + OE PSS2 mice. (b-d) qRT-PCR analysis of inflammatory and fibrosis gene expression in different groups of mice (*n* = 6 per group). (e and f) Representative TEM micrographs and quantitative analysis of lipid droplet areas in renal tubular epithelial cells from different groups of mice (*n* = 5 per group) (Scale bars = 2 μm). (g-j) Representative Western blot images and quantitative analysis of ER stress markers (XBP-1 s, p-EIF2α, EIF2α and CHOP) in different groups of mice (*n* = 6 per group). (k) Representative Western blot images and quantitative analysis of PSS2 expression efficiency in LvNC-TCMK-1 cells and Lv*Pss2*-TCMK-1 cells (*n* = 3 independent replicates). (l-n) qRT-PCR analysis of inflammatory and fibrosis gene expression in TCMK-1 cells subjected to different treatments (*n* = 3 independent replicates). (o and p) Representative images and quantitative analysis of the Oil Red O-stained areas of TCMK-1 cells subjected to different treatments (*n* = 3 independent replicates) (Scale bars = 100 μm). (q) Quantification of ER-mitochondria colocalization (Pearson's correlation coefficient) in PSS2-overexpressing TCMK-1 cells compared with control cells under different treatments (*n* = 15 microscopic fields from three independent experiments). The data in the graphs are presented as the means ± SDs. The data were analysed via an unpaired two-tailed Student's t test (a and h-k) and two-sided one-way ANOVA with Tukey's test (b-d, f, l-n, p and q). ns, not significant; *$P < 0.05$; **$P < 0.01$; ***$P < 0.001$; ****$P < 0.0001$.

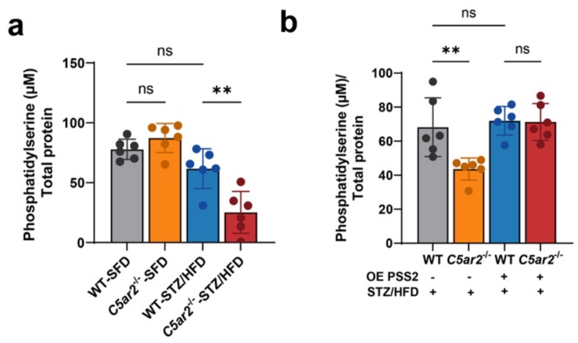

Supplementary Figure S7. PSS expression regulates PS content in the MAM.

(a) Enzyme-linked immunosorbent assay (ELISA) quantification of PS content in MAM fractions isolated from the renal cortex of diabetic *C5ar2*$^{-/-}$ mice and diabetic WT mice (*n* = 6 per group). (b) ELISA quantification of PS content in MAM fractions from STZ/HFD-treated mice with or without PSS2 overexpression (*n* = 6 per group). The data in the graphs are presented as the means ± SDs. The data were analysed by two-sided one-way ANOVA with Tukey's test. ns, not significant; **$P < 0.01$.

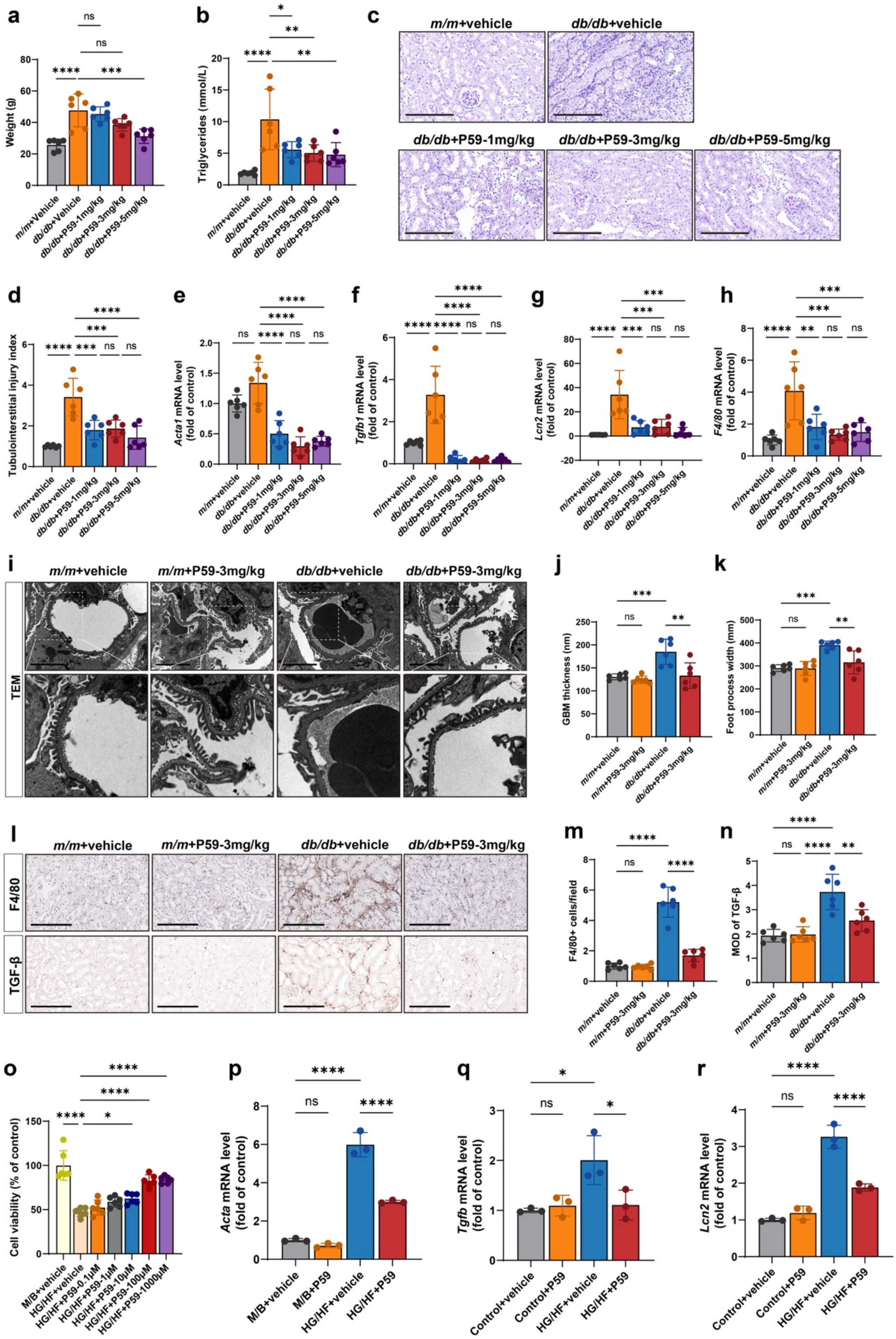
a
Weight (g)
m/m+Vehicle
db/db+Vehicle
db/db+P59-1mg/kg
db/db+P59-3mg/kg
db/db+P59-5mg/kg
b
Triglycerides (mmol/L)
c
m/m+vehicle
db/db+vehicle
db/db+P59-1mg/kg
db/db+P59-3mg/kg
db/db+P59-5mg/kg
d
Tubulointerstitial injury index
e
Acta1 mRNA level (fold of control)
f
Tgfb1 mRNA level (fold of control)
g
Lcn2 mRNA level (fold of control)
h
F4/80 mRNA level (fold of control)
i
TEM
m/m+vehicle
m/m+P59-3mg/kg
db/db+vehicle
db/db+P59-3mg/kg
j
GBM thickness (nm)
k
Foot process width (nm)
l
F4/80
TGF-β
m
F4/80+ cells/field
n
MOD of TGF-β
o
Cell viability (% of control)
M/B+vehicle
HG/HF+vehicle
HG/HF+P59-0.1μM
HG/HF+P59-1μM
HG/HF+P59-10μM
HG/HF+P59-100μM
HG/HF+P59-1000μM
p
Acta mRNA level (fold of control)
M/B+vehicle
M/B+P59
HG/HF+vehicle
HG/HF+P59
q
Tgfb mRNA level (fold of control)
Control+vehicle
Control+P59
HG/HF+vehicle
HG/HF+P59
r
Lcn2 mRNA level (fold of control)

Supplementary Figure S8. The C5aR2 agonist P59 alleviated tubulointerstitial injury in DKD. (a) Comparison of the weights of the different groups of mice (*n* = 6 per group). (b) Comparison of blood triglyceride levels in different groups of mice (*n* = 6 per group). (c) Representative images of PAS staining in different groups of mice (Scale bars = 200 μm). (d) Quantitative analysis of the tubulointerstitial injury index in different groups of mice. (e-h) qRT-PCR analysis of inflammatory and fibrosis gene expression in different groups of mice (*n* = 6 per group). (i) Representative TEM micrographs (Scale bars = 5 μm). Quantification of (j) mean GBM thickness and (k) mean foot process width in the 4 groups of mice. (l) Representative IHC images of F4/80 and TGF-β staining (Scale bars = 200 μm). Quantitative analysis of (m) F4/80 and (n) TGF-β staining in different groups of mice (*n* = 6 per group). (o) CCK8 assay showing the effect of P59 at all concentrations on the viability of HG/HF-treated TCMK-1 cells (*n* = 3 independent replicates). (p-r) qRT-PCR analysis of inflammatory and fibrosis gene expression in TCMK-1 cells subjected to different treatments. The data were analysed by two-sided one-way ANOVA with Tukey's test. ns, not significant; *$P < 0.05$; **$P < 0.01$; ***$P < 0.001$; ****$P < 0.0001$.

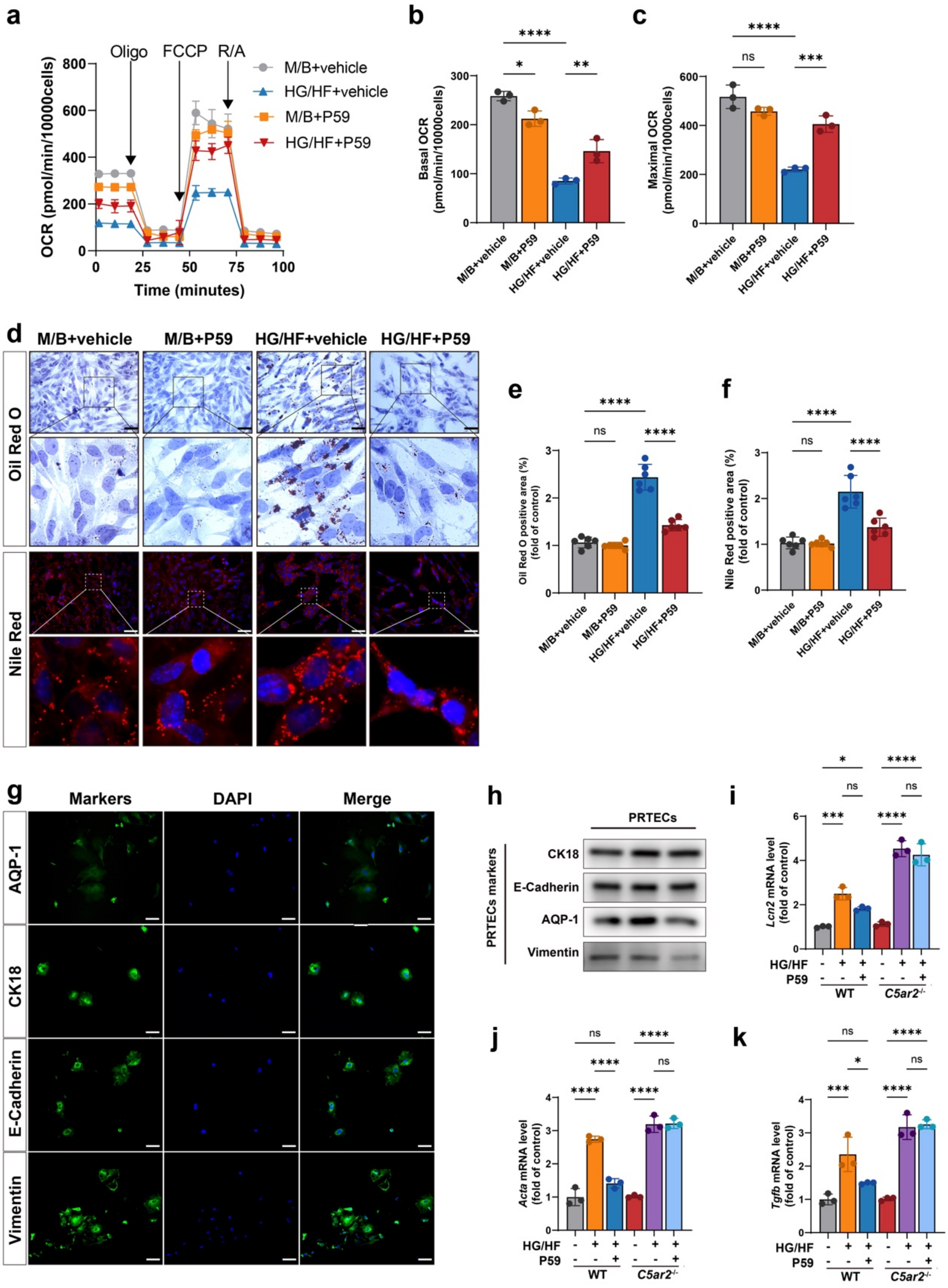
a
Oligo
FCCP
R/A
M/B+vehicle
HG/HF+vehicle
M/B+P59
HG/HF+P59
OCR (pmol/min/10000cells)
Time (minutes)
b
Basal OCR (pmol/min/10000cells)
c
Maximal OCR (pmol/min/10000cells)
d
M/B+vehicle
M/B+P59
HG/HF+vehicle
HG/HF+P59
Oil Red O
Nile Red
e
Oil Red O positive area (%) (fold of control)
f
Nile Red positive area (%) (fold of control)
g
Markers
DAPI
Merge
AQP-1
CK18
E-Cadherin
Vimentin
h
PRTECs
PRTECs markers
CK18
E-Cadherin
AQP-1
Vimentin
i
Lcn2 mRNA level (fold of control)
HG/HF
P59
WT
C5ar2-/-
j
Acta mRNA level (fold of control)
k
Tgfb mRNA level (fold of control)

Supplementary Figure S9. The C5aR2 agonist P59 alleviated metabolic disorders in DKD. (a-c) Mitochondrial oxygen consumption rate (OCR) analysis of TCMK-1 cells in the presence or absence of HG/HF or P59 treatment. (a) Representative OCR curves. (b and c) Quantification of basal and maximal OCR (n = 3 independent experiments). (d-f) Representative images and quantitative analysis of Oil Red O staining and Nile Red staining of TCMK-1 cells subjected to different treatments (n = 6 independent replicates). (g) Representative immunofluorescence images of PRTECs (Cytokeratin 18 [CK18], Vimentin, AQP-1, and E-Cadherin). (h) Western blot analysis of PRTEC markers (CK18, Vimentin, AQP-1, and E-Cadherin) confirming cell identity. (i-k) qRT-PCR analysis showing *Lcn2*, *Acta1*, and *Tgfb1* mRNA levels in PRTECs isolated from WT and *C5ar2*$^{-/-}$ mice treated with P59 under HG/HF conditions (n = 3 independent replicates). The data in the graphs are presented as the means ± SDs. The data were analysed by two-sided one-way ANOVA with Tukey's test. ns, not significant; *$P < 0.05$; **$P < 0.01$; ***$P < 0.001$; ****$P < 0.0001$.

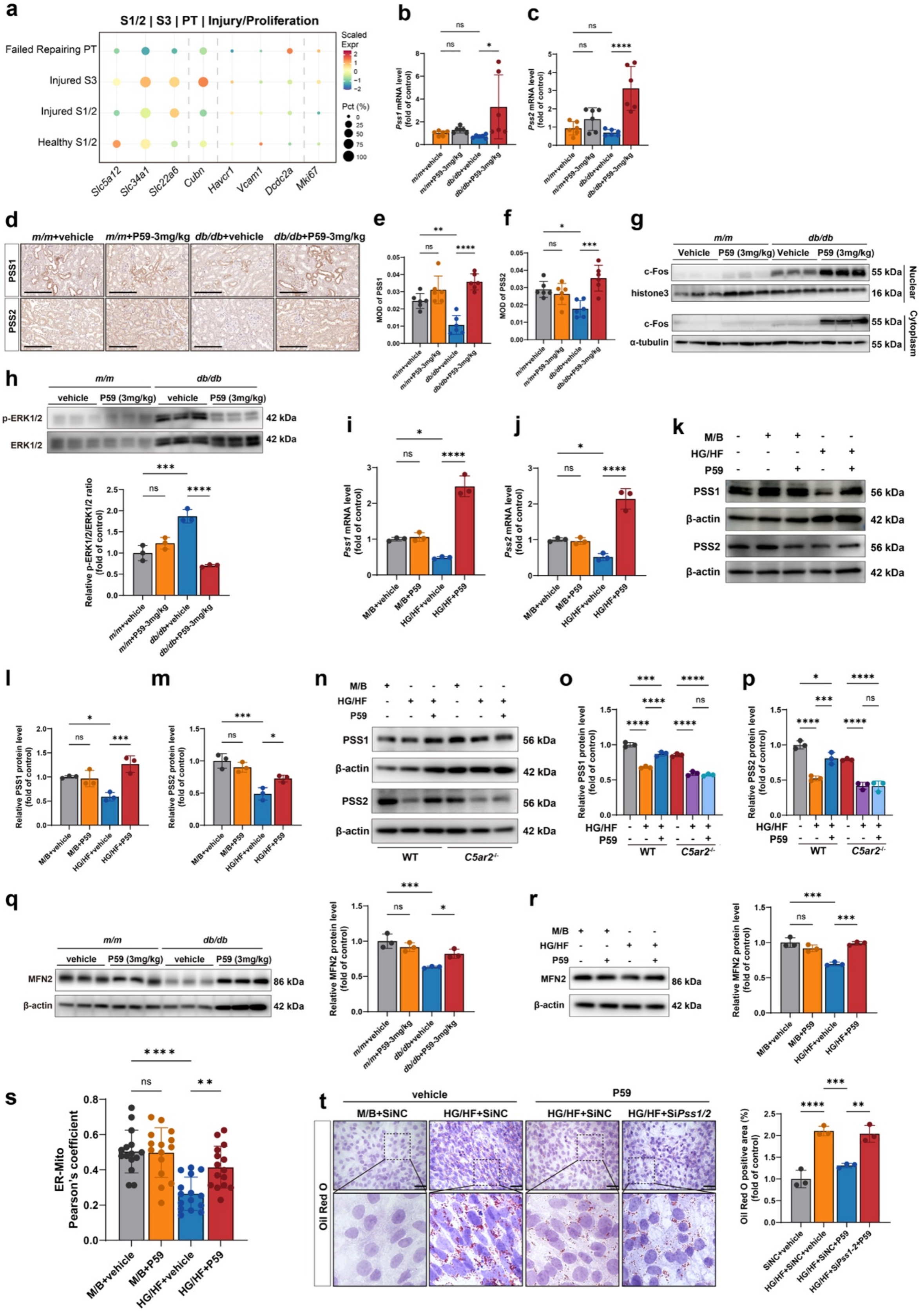
a
S1/2 | S3 | PT | Injury/Proliferation
Failed Repairing PT
Injured S3
Injured S1/2
Healthy S1/2
Slc5a12
Slc34a1
Slc22a6
Cubn
Havcr1
Vcam1
Dcdc2a
Mki67
Scaled Expr
Pct (%)
b
Pss1 mRNA level (fold of control)
c
Pss2 mRNA level (fold of control)
m/m+vehicle
m/m+P59-3mg/kg
db/db+vehicle
db/db+P59-3mg/kg
d
PSS1
PSS2
e
MOD of PSS1
f
MOD of PSS2
g
m/m
db/db
Vehicle
P59 (3mg/kg)
c-Fos
histone3
α-tubulin
55 kDa
16 kDa
Nuclear
Cytoplasm
h
p-ERK1/2
ERK1/2
42 kDa
Relative p-ERK1/2/ERK1/2 ratio (fold of control)
i
j
M/B+vehicle
M/B+P59
HG/HF+vehicle
HG/HF+P59
k
M/B
HG/HF
P59
PSS1
β-actin
PSS2
56 kDa
42 kDa
l
Relative PSS1 protein level (fold of control)
m
Relative PSS2 protein level (fold of control)
n
WT
C5ar2-/-
o
p
q
MFN2
86 kDa
Relative MFN2 protein level (fold of control)
r
s
ER-Mito Pearson's coefficient
t
vehicle
P59
M/B+SiNC
HG/HF+SiNC
HG/HF+SiPss1/2
Oil Red O
Oil Red O positive area (%) (fold of control)
SiNC+vehicle
HG/HF+SiNC+vehicle
HG/HF+SiNC+P59
HG/HF+SiPss1-2+P59

Supplementary Figure S10. P59 regulates PSS1/2 and MFN2 expression and improves lipid metabolism. (a) Schematic representation of marker gene expression profiles used to define PT subpopulations: healthy PTS1/2, injured PTS1/2, injured PTS3, and failed repairing PT cells. (b and c) qRT-PCR analysis of *Pss1* and *Pss2* expression in the renal cortex of different groups of mice (n = 6 per group). (d-f) Representative IHC images and quantitative analysis of PSS1 and PSS2 expression in the renal cortex (n = 6 per group) (Scale bars = 200 μm). (g) Nucleocytoplasmic fractionation and Western blot analysis of c-FOS in TCMK-1 cells under different treatments. Histone 3 and α-tubulin served as internal controls for the nucleus and cytoplasm, respectively. Representative results of three independent biological experiments are shown. (h) Representative Western blot images and quantitative analysis of ERK 1/2 phosphorylation (p-ERK1/2/ERK1/2) in the renal cortex of vehicle-treated and P59-treated *db/db* mice (n = 3 per group). (i and j) qRT-PCR analysis of *Pss1* and *Pss2* gene expression in TCMK-1 cells subjected to different treatments (n = 3 independent replicates). (k-m) Representative Western blot images and quantitative analysis of PSS1 and PSS2 expression in TCMK-1 cells subjected to different treatments (n = 3 independent replicates). (n-p) Representative Western blot images and quantitative analysis of PSS1 and PSS2 protein expression in PRTECs isolated from WT and *C5ar2*$^{-/-}$ mice treated with P59 under HG/HF conditions (n = 3 per group). (q) Representative Western blot images and quantitative analysis of MFN2 expression in the renal cortex of vehicle-treated and P59-treated *db/db* mice (n = 3 per group). (r) Representative Western blot images and quantitative analysis of MFN2 expression in vehicle-treated and P59-treated TCMK-1 cells under HG/HF conditions (n = 3 independent replicates). (s) Quantification of ER-mitochondria colocalization (Pearson's correlation coefficient) in P59 treated-TCMK-1 cells compared with control cells under different treatments (n = 15 microscopic fields from three independent experiments). (t) Representative images and quantitative analysis of Oil Red O-stained area of TCMK-1 cells subjected to different treatments (n = 3 independent replicates) (Scale bars = 50 μm). The data in the graphs are presented as the means ± SDs. The data were

analysed by two-sided one-way ANOVA with Tukey's test. ns, not significant; $*P < 0.05$; $**P < 0.01$; $***P < 0.001$; $****P < 0.0001$.
